# Supplementary material for: Reelin-LRP8 signaling mediates brain dissemination of breast cancer cells via abluminal migration
Source: EMBO Mol Med. 2025 Jun 12;17(8):1983–2010. doi: 10.1038/s44321-025-00260-0 (PMC12339728; doi:10.1038/s44321-025-00260-0)
Supplement: Supplementary file 6 — Movie EV1 [file 44321_2025_260_MOESM6_ESM.zip › Movie EV1.docx]

**Movie EV1.** Three-dimensional reconstruction was performed in lateral view to visualize the interaction between transplanted MDA-MB-231 cells and the PCeV in zebrafish. The MDA-MB-231 cells are shown in green, and the PceV is depicted in red. Scale bar: 20 μm.
