# Supplementary material for: Reelin-LRP8 signaling mediates brain dissemination of breast cancer cells via abluminal migration
Source: EMBO Mol Med. 2025 Jun 12;17(8):1983–2010. doi: 10.1038/s44321-025-00260-0 (PMC12339728; doi:10.1038/s44321-025-00260-0)
Supplement: Supplementary file 7 — Movie EV2 [file 44321_2025_260_MOESM7_ESM.zip › Movie EV2.docx]

**Movie EV2.** Three-dimensional reconstruction was performed in lateral view to visualize the position of MDA-MB-231 cells in zebrafish brain. The MDA-MB-231 cells are shown in green and blood vessels are shown in pink. Skin stained by Alexa Fluor™ 647 cadaverine, is shown in purple. Scale bar: 50 μm.
