# Supplementary material for: Reelin-LRP8 signaling mediates brain dissemination of breast cancer cells via abluminal migration
Source: EMBO Mol Med. 2025 Jun 12;17(8):1983–2010. doi: 10.1038/s44321-025-00260-0 (PMC12339728; doi:10.1038/s44321-025-00260-0)
Supplement: Supplementary file 10 — Movie EV5 [file 44321_2025_260_MOESM10_ESM.zip › Movie EV5.docx]

**Movie EV5.** Three-dimensional reconstruction was performed to visualize the mouse brain of sham operation group. Scale bar: 1000 μm.
