# Supplementary material for: Reelin-LRP8 signaling mediates brain dissemination of breast cancer cells via abluminal migration
Source: EMBO Mol Med. 2025 Jun 12;17(8):1983–2010. doi: 10.1038/s44321-025-00260-0 (PMC12339728; doi:10.1038/s44321-025-00260-0)
Supplement: Supplementary file 12 — Movie EV7 [file 44321_2025_260_MOESM12_ESM.zip › Movie EV7.docx]

**Movie EV7.** Three-dimensional reconstruction was performed to visualize the brain metastatic tumor treated with MEN 10207 in nude mice xenograft model. The transplanted MDA-MB-231 cells in brain are shown in green. Scale bar: 1000 μm.
