# Supplementary figures and images for: Reelin-LRP8 signaling mediates brain dissemination of breast cancer cells via abluminal migration
Source: EMBO Mol Med. 2025 Jun 12;17(8):1983–2010. doi: 10.1038/s44321-025-00260-0 (PMC12339728; doi:10.1038/s44321-025-00260-0)

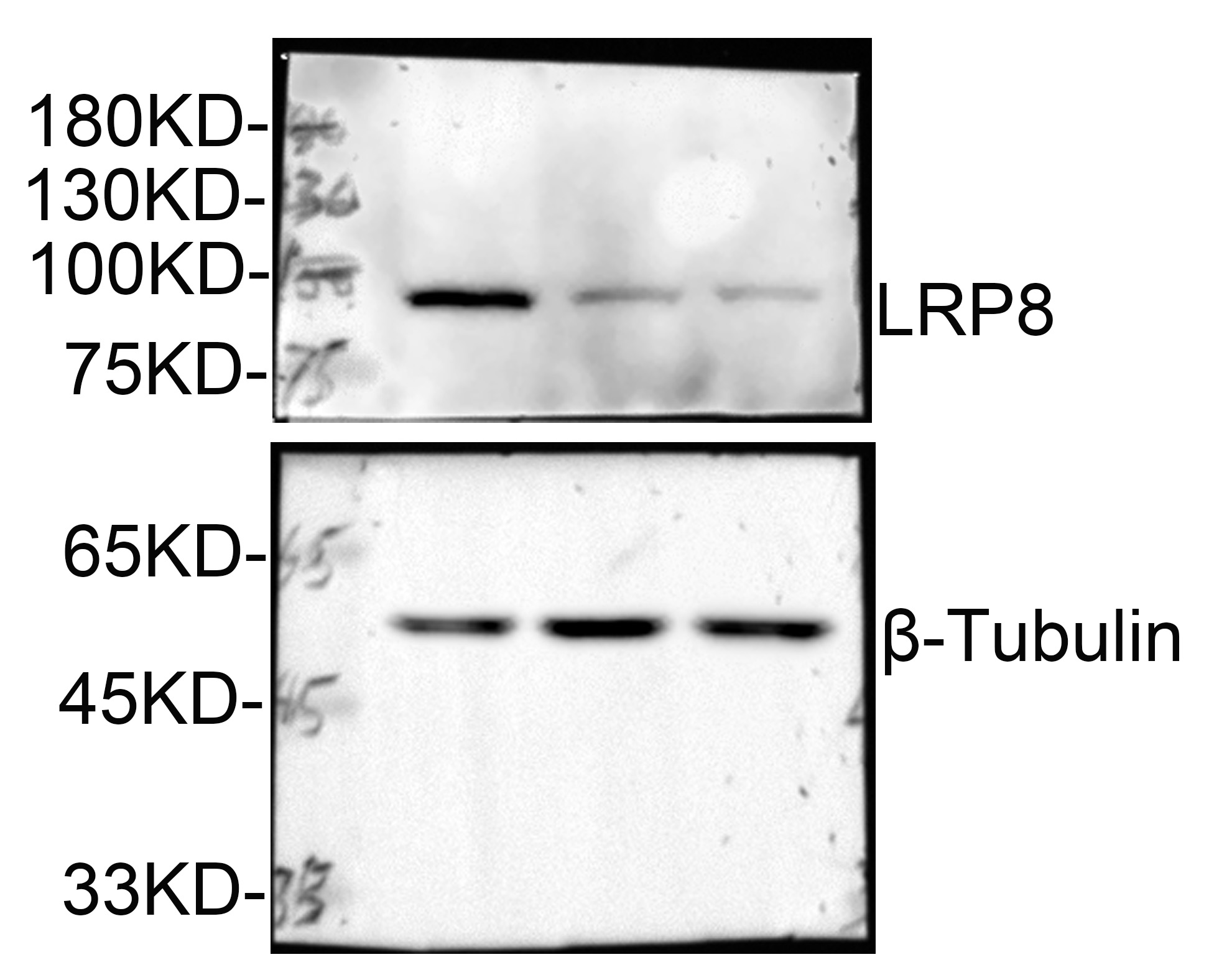

Supplement: Supplementary file 19 — Figure EV2 Source Data [file 44321_2025_260_MOESM19_ESM.zip › Figure EV2/EV2A/EV2A Blot.jpg]

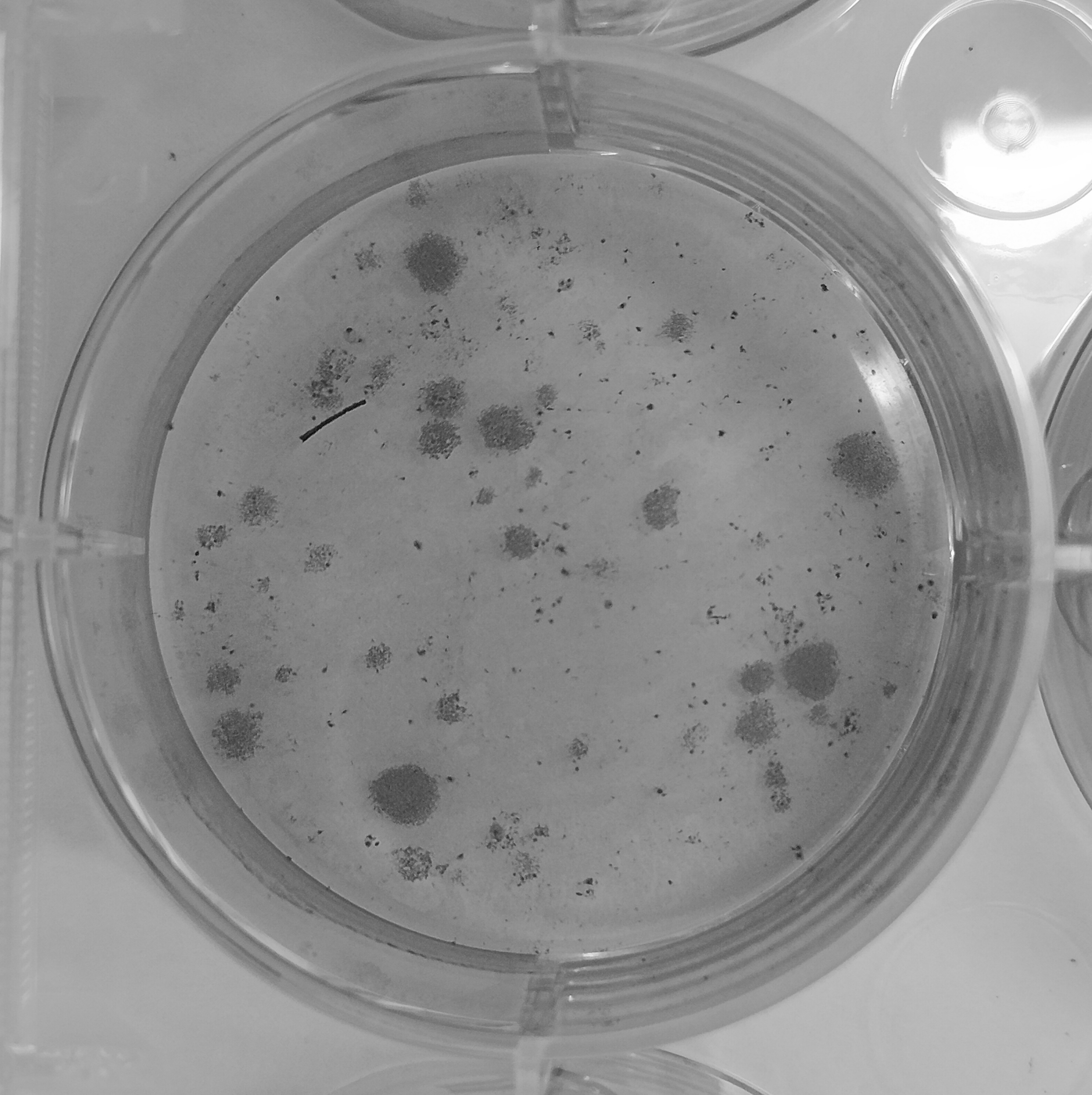

Supplement: Supplementary file 19 — Figure EV2 Source Data [file 44321_2025_260_MOESM19_ESM.zip › Figure EV2/EV2D/sh-ctrl-.png]

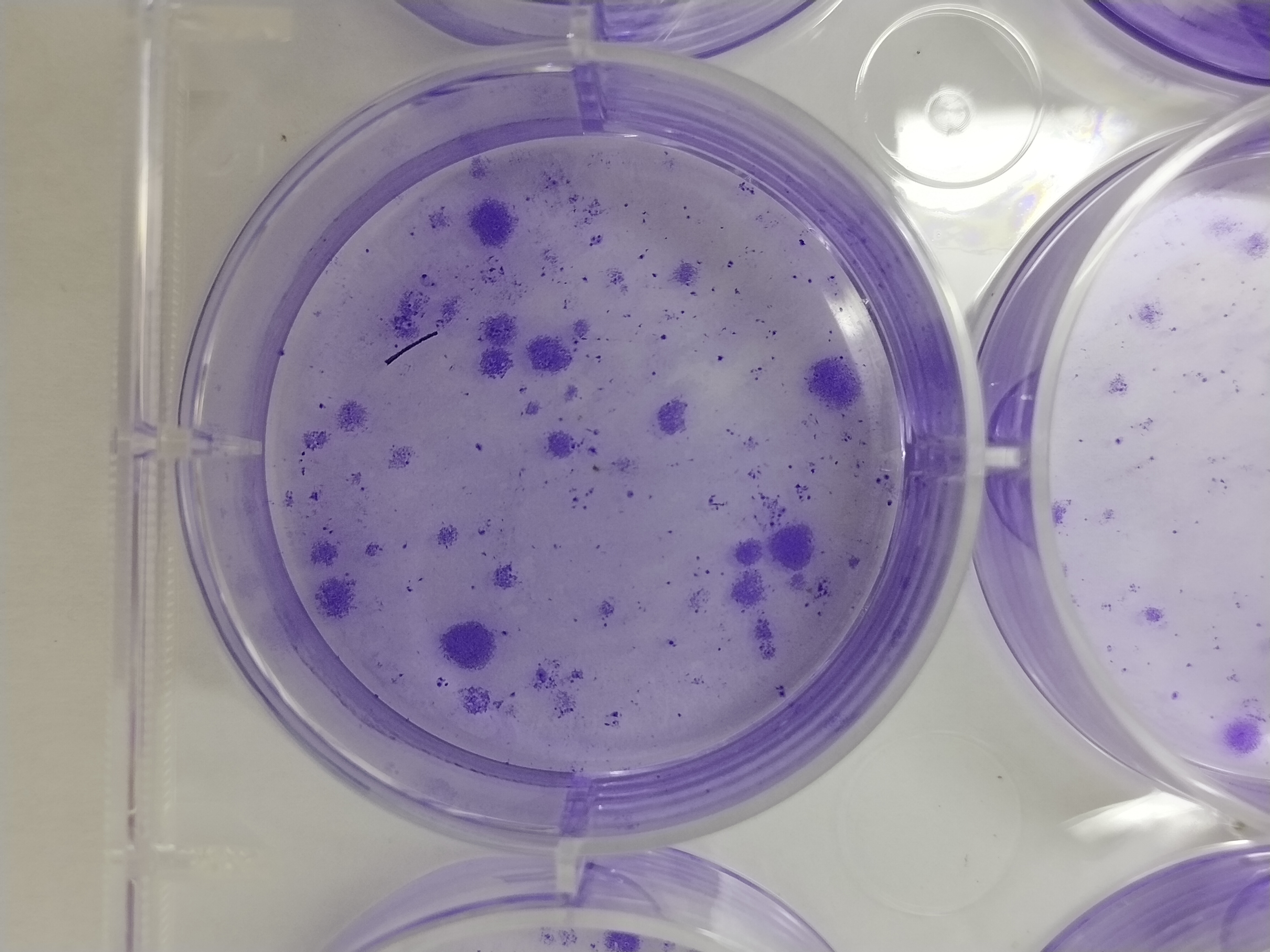

Supplement: Supplementary file 19 — Figure EV2 Source Data [file 44321_2025_260_MOESM19_ESM.zip › Figure EV2/EV2D/sh-ctrl.jpg]

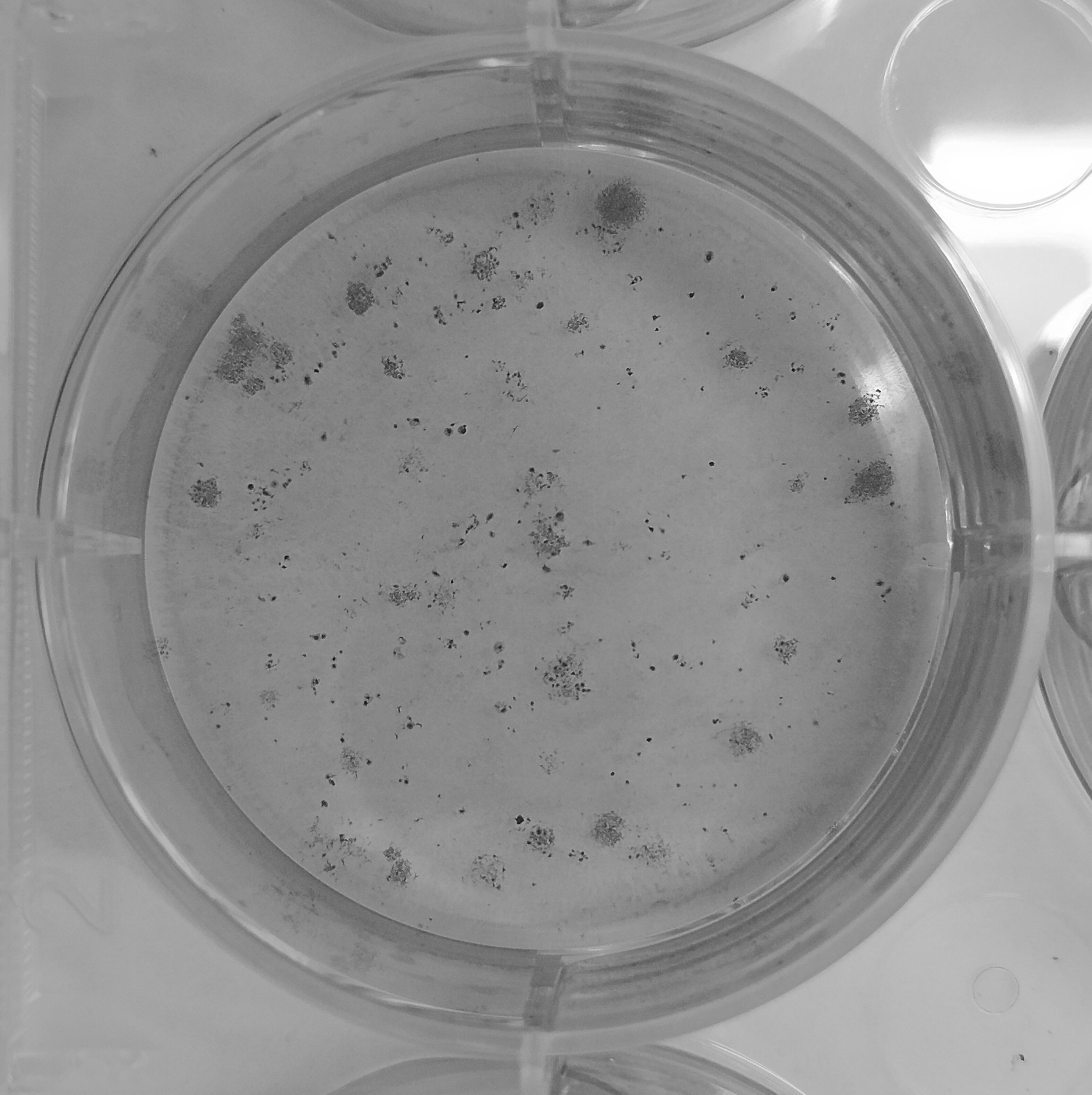

Supplement: Supplementary file 19 — Figure EV2 Source Data [file 44321_2025_260_MOESM19_ESM.zip › Figure EV2/EV2D/sh-LRP8-1#-.png]

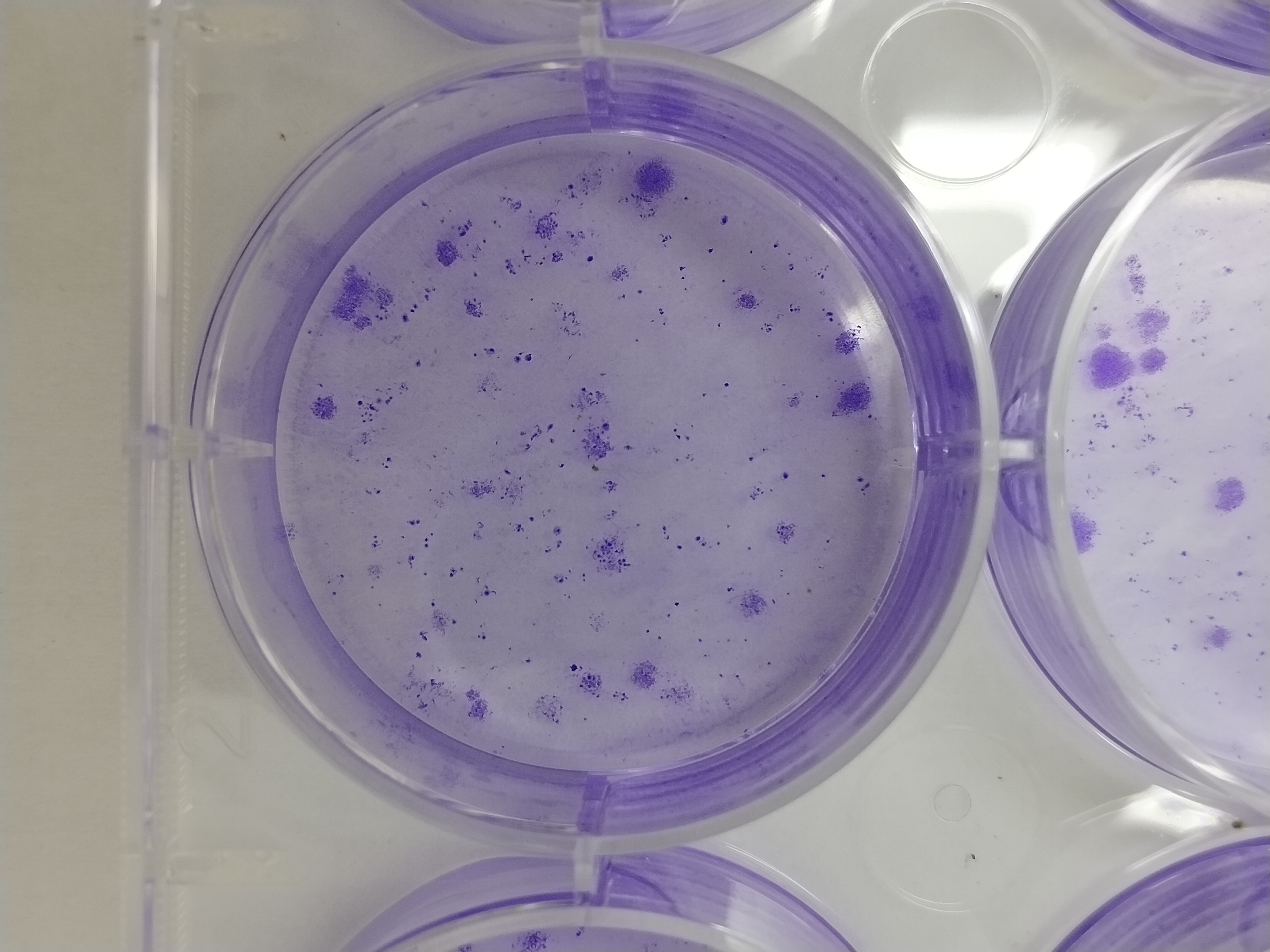

Supplement: Supplementary file 19 — Figure EV2 Source Data [file 44321_2025_260_MOESM19_ESM.zip › Figure EV2/EV2D/sh-LRP8-1#.jpg]

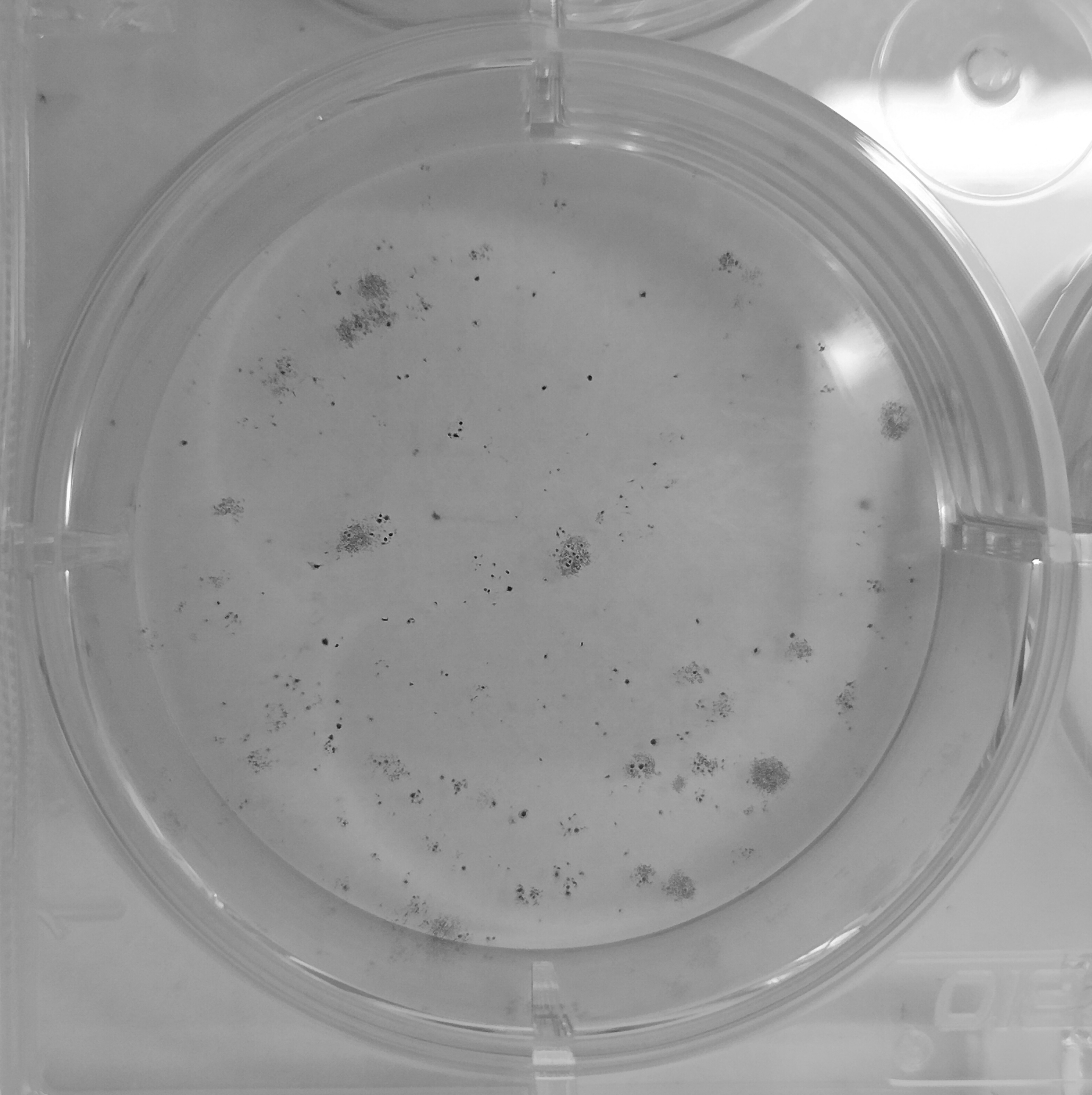

Supplement: Supplementary file 19 — Figure EV2 Source Data [file 44321_2025_260_MOESM19_ESM.zip › Figure EV2/EV2D/sh-LRP8-2#-.png]

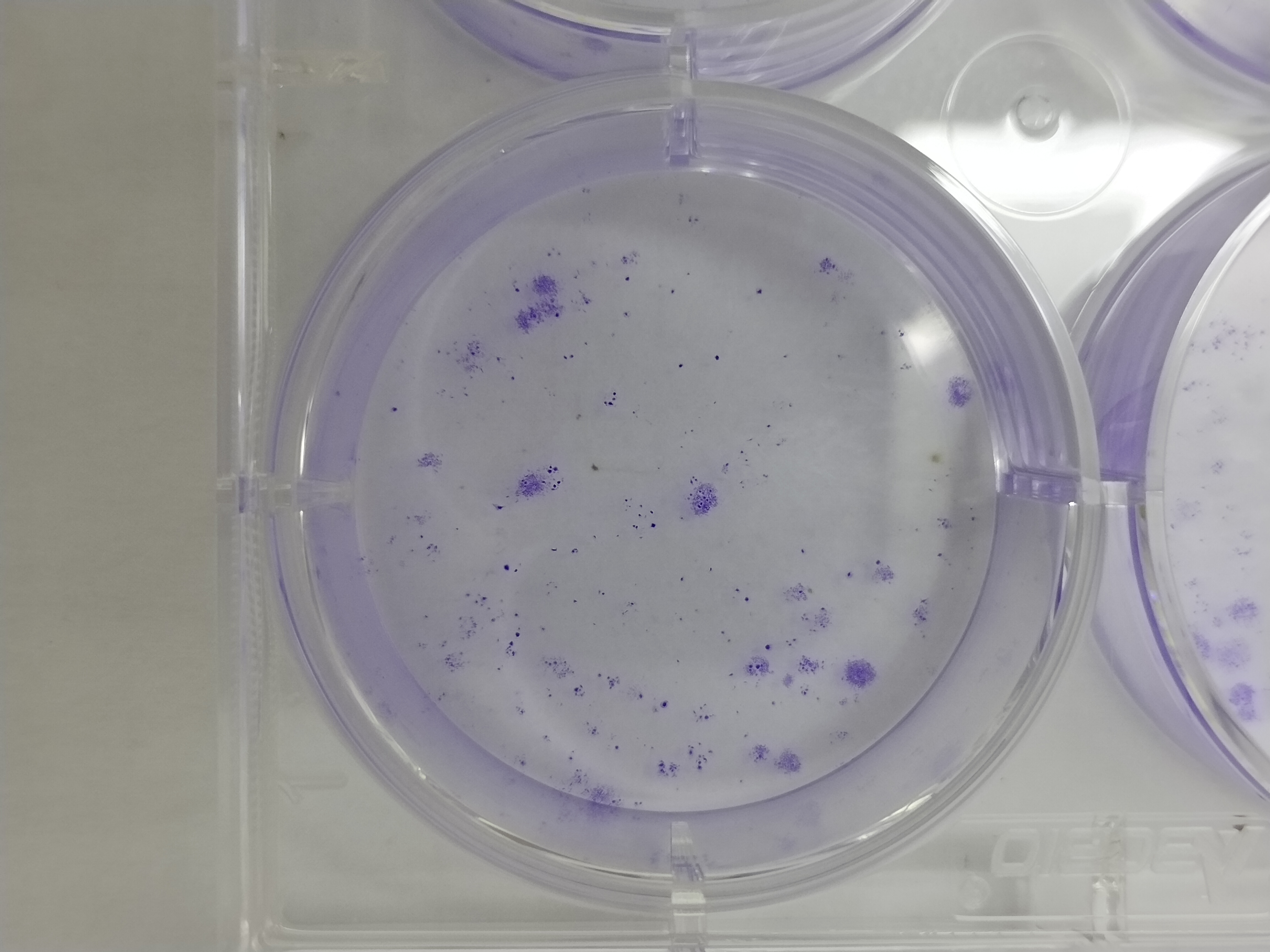

Supplement: Supplementary file 19 — Figure EV2 Source Data [file 44321_2025_260_MOESM19_ESM.zip › Figure EV2/EV2D/sh-LRP8-2#.jpg]

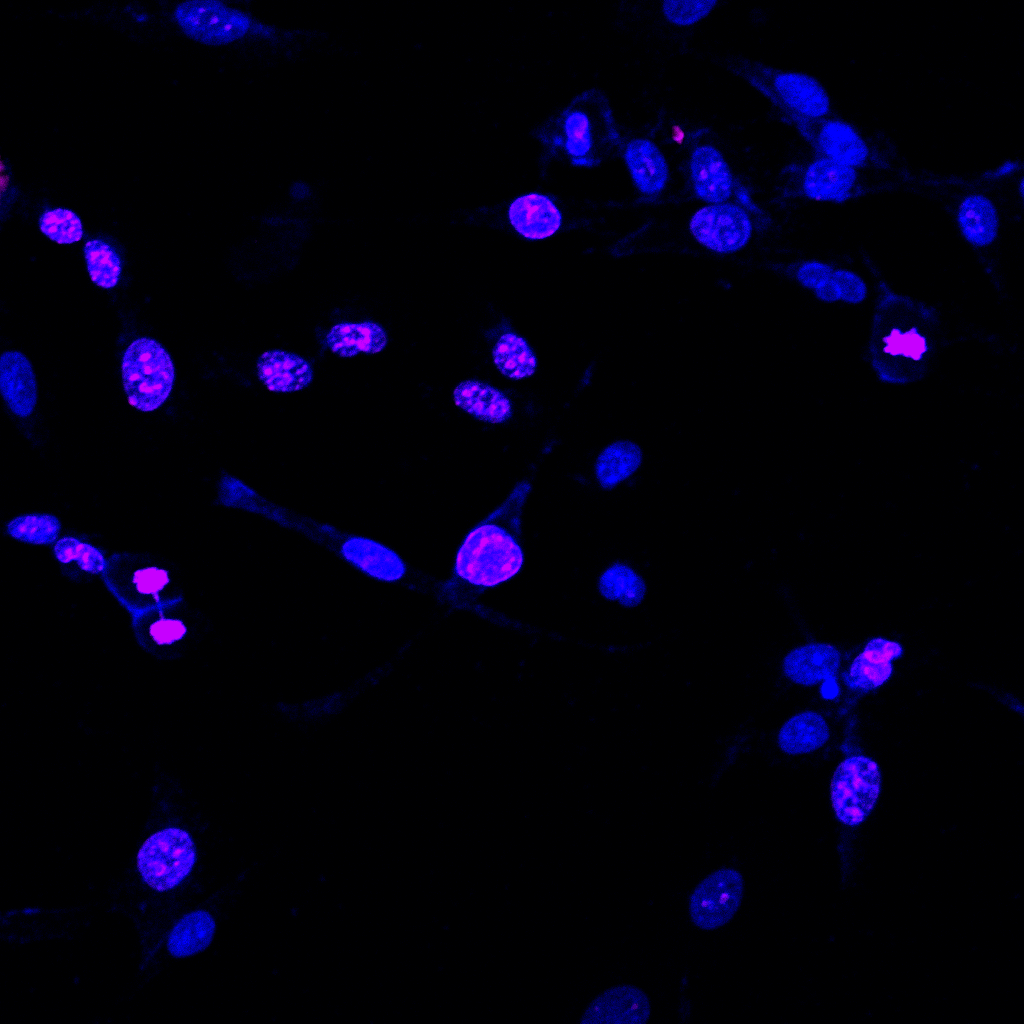

Supplement: Supplementary file 19 — Figure EV2 Source Data [file 44321_2025_260_MOESM19_ESM.zip › Figure EV2/EV2F/sh-ctrl X6.png]

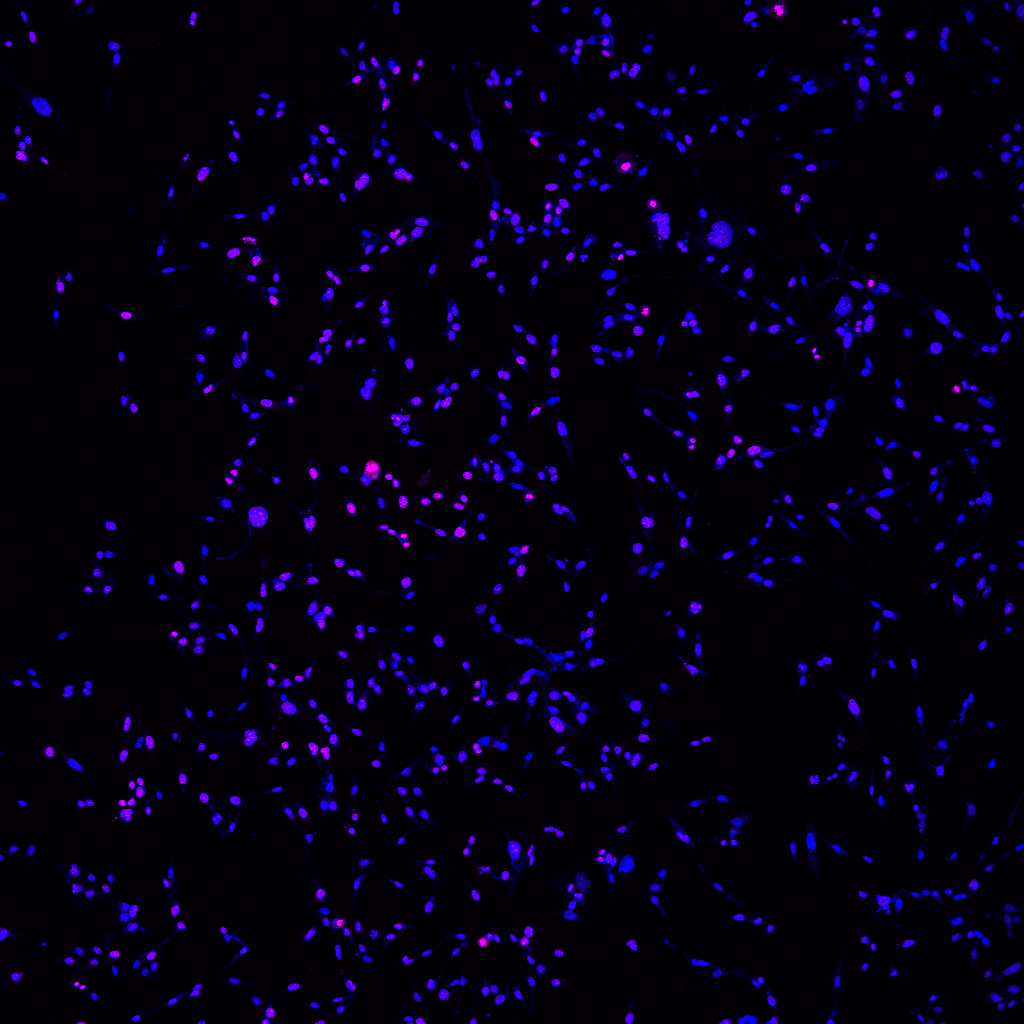

Supplement: Supplementary file 19 — Figure EV2 Source Data [file 44321_2025_260_MOESM19_ESM.zip › Figure EV2/EV2F/sh-ctrl.png]

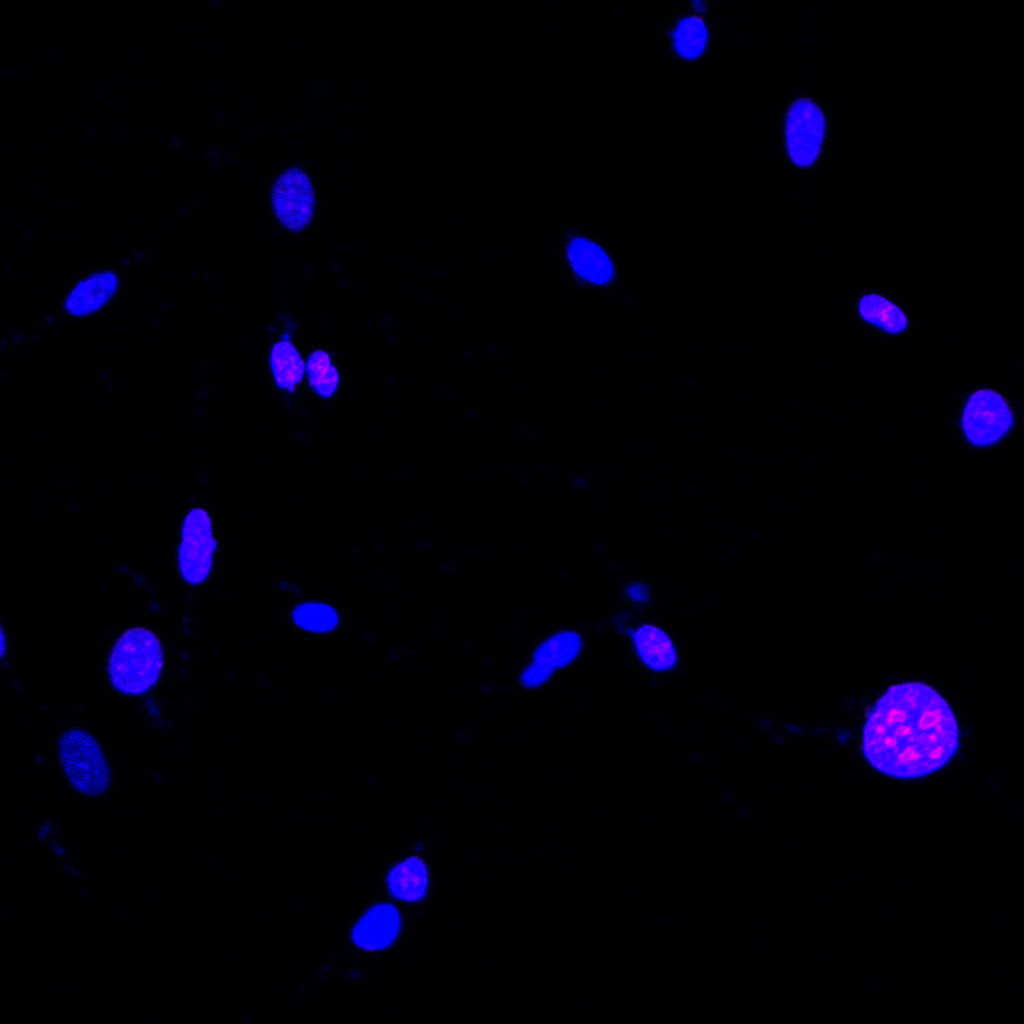

Supplement: Supplementary file 19 — Figure EV2 Source Data [file 44321_2025_260_MOESM19_ESM.zip › Figure EV2/EV2F/sh-LRP8-1# X6.png]

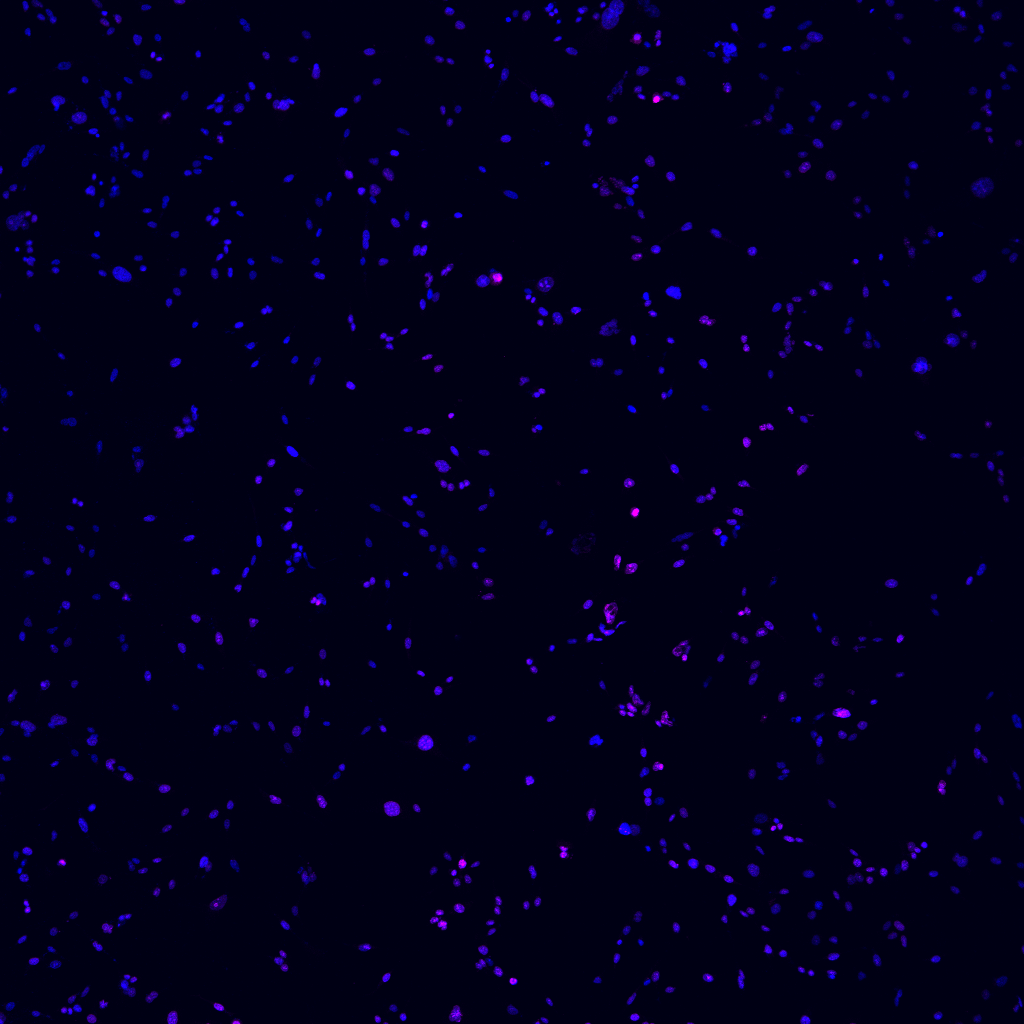

Supplement: Supplementary file 19 — Figure EV2 Source Data [file 44321_2025_260_MOESM19_ESM.zip › Figure EV2/EV2F/sh-LRP8-1#.png]

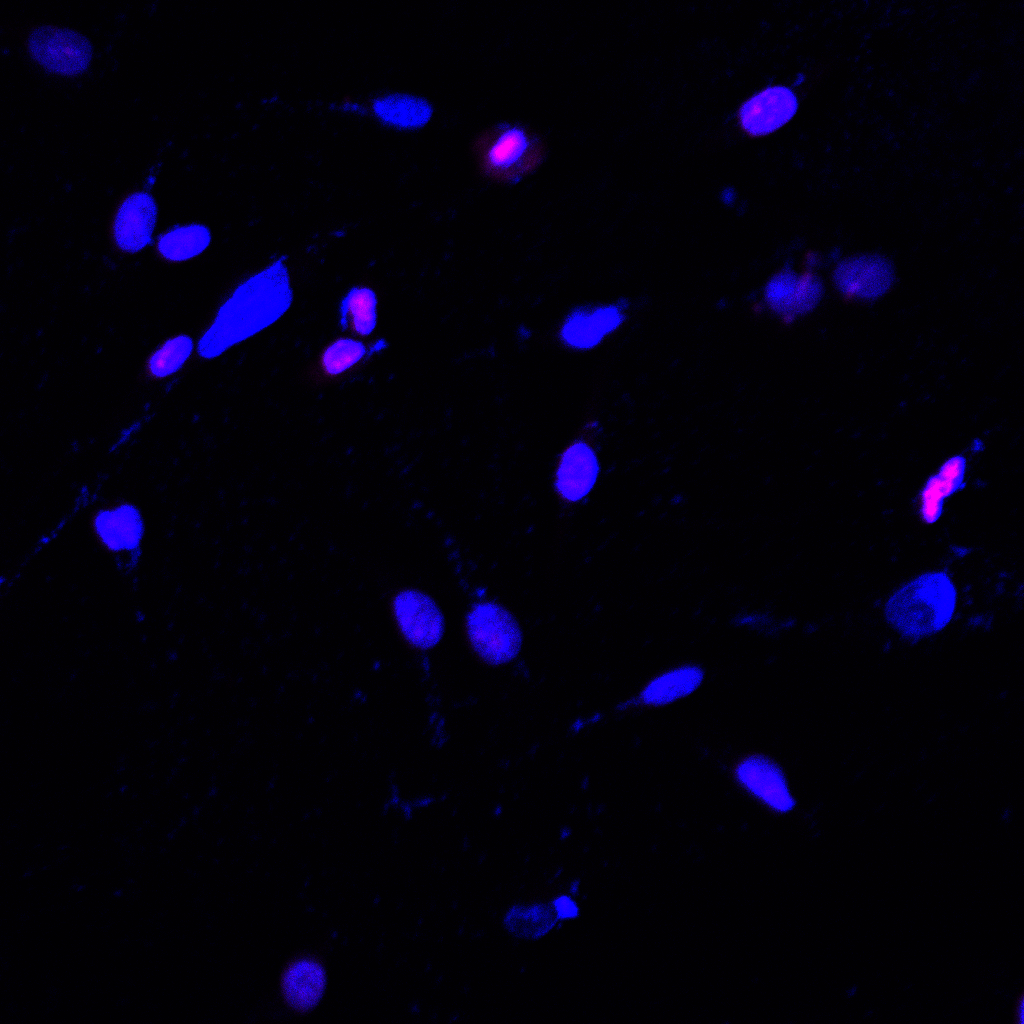

Supplement: Supplementary file 19 — Figure EV2 Source Data [file 44321_2025_260_MOESM19_ESM.zip › Figure EV2/EV2F/sh-LRP8-2# X6.png]

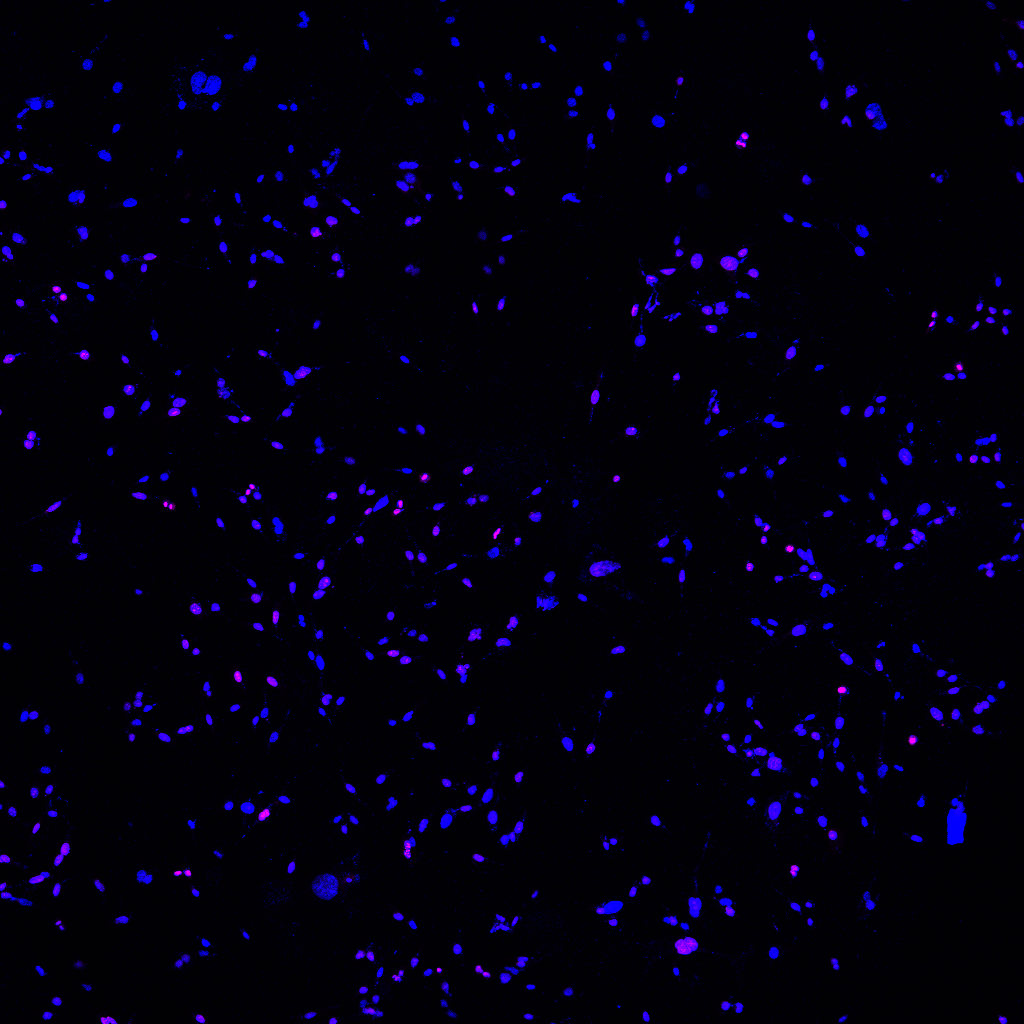

Supplement: Supplementary file 19 — Figure EV2 Source Data [file 44321_2025_260_MOESM19_ESM.zip › Figure EV2/EV2F/sh-LRP8-2#.png]

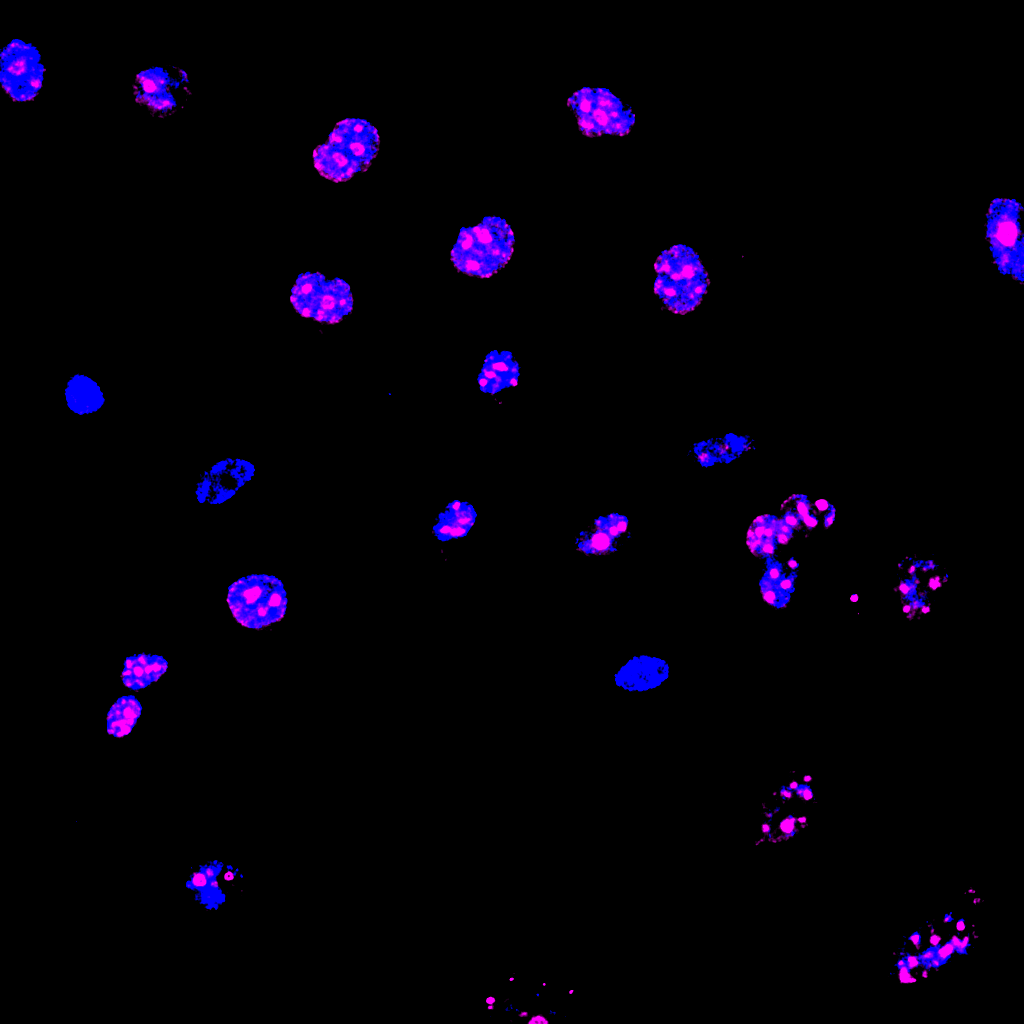

Supplement: Supplementary file 19 — Figure EV2 Source Data [file 44321_2025_260_MOESM19_ESM.zip › Figure EV2/EV2H/sh-ctrl 6X.tif]

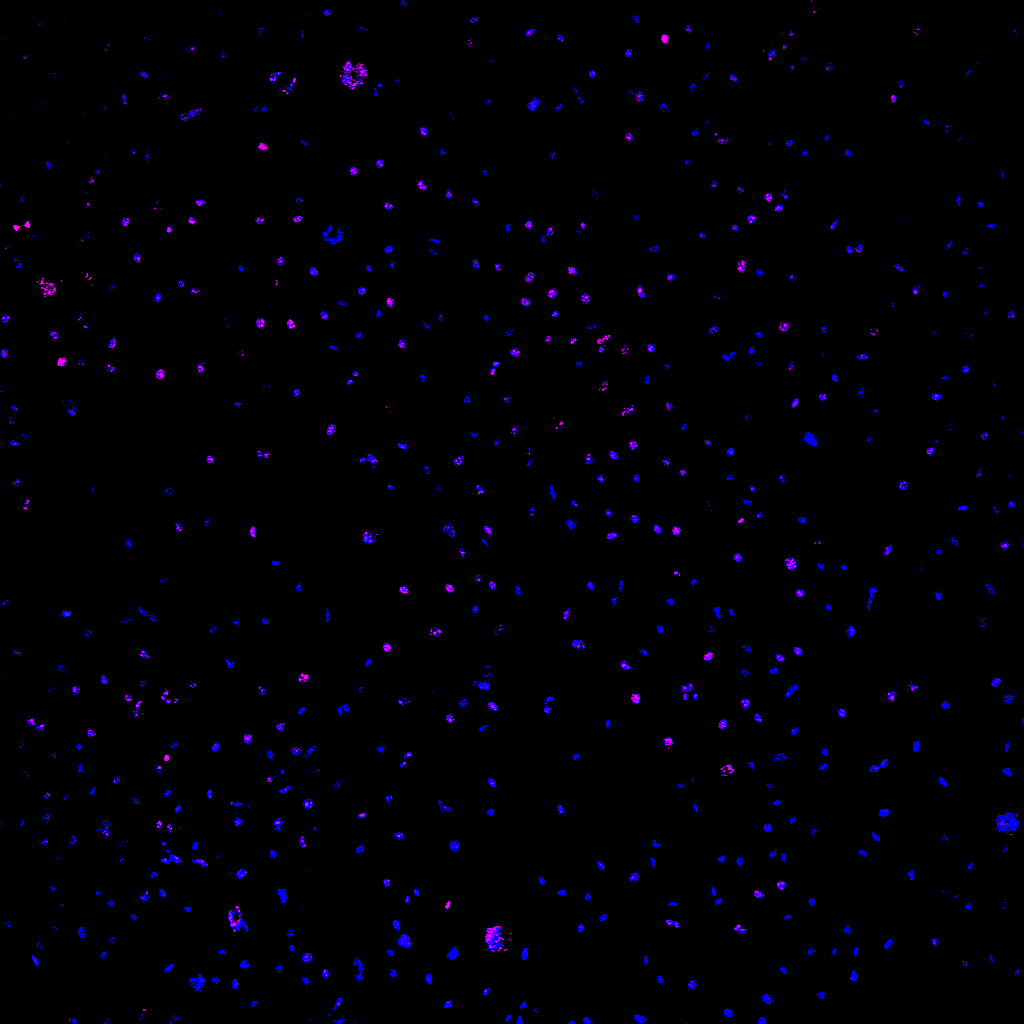

Supplement: Supplementary file 19 — Figure EV2 Source Data [file 44321_2025_260_MOESM19_ESM.zip › Figure EV2/EV2H/sh-ctrl.tif]

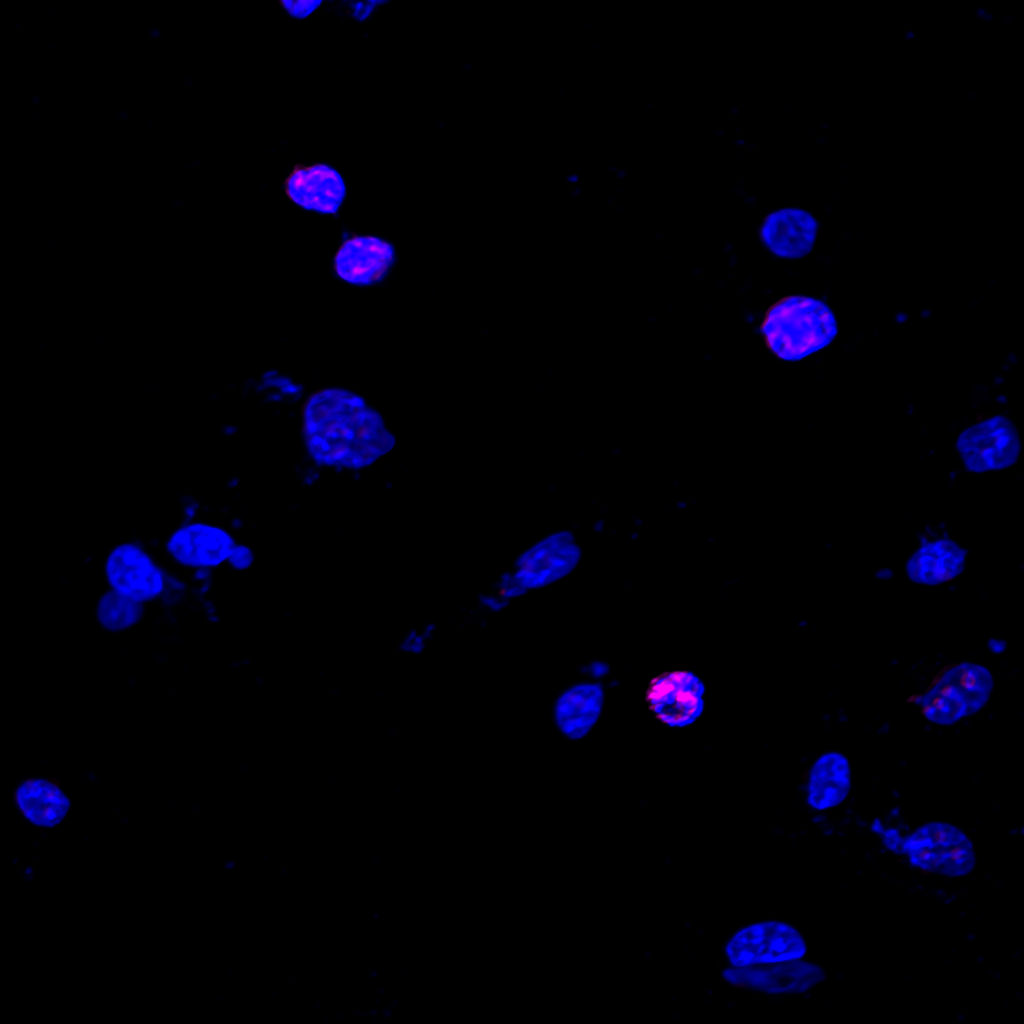

Supplement: Supplementary file 19 — Figure EV2 Source Data [file 44321_2025_260_MOESM19_ESM.zip › Figure EV2/EV2H/sh-LRP8-1# X6.png]

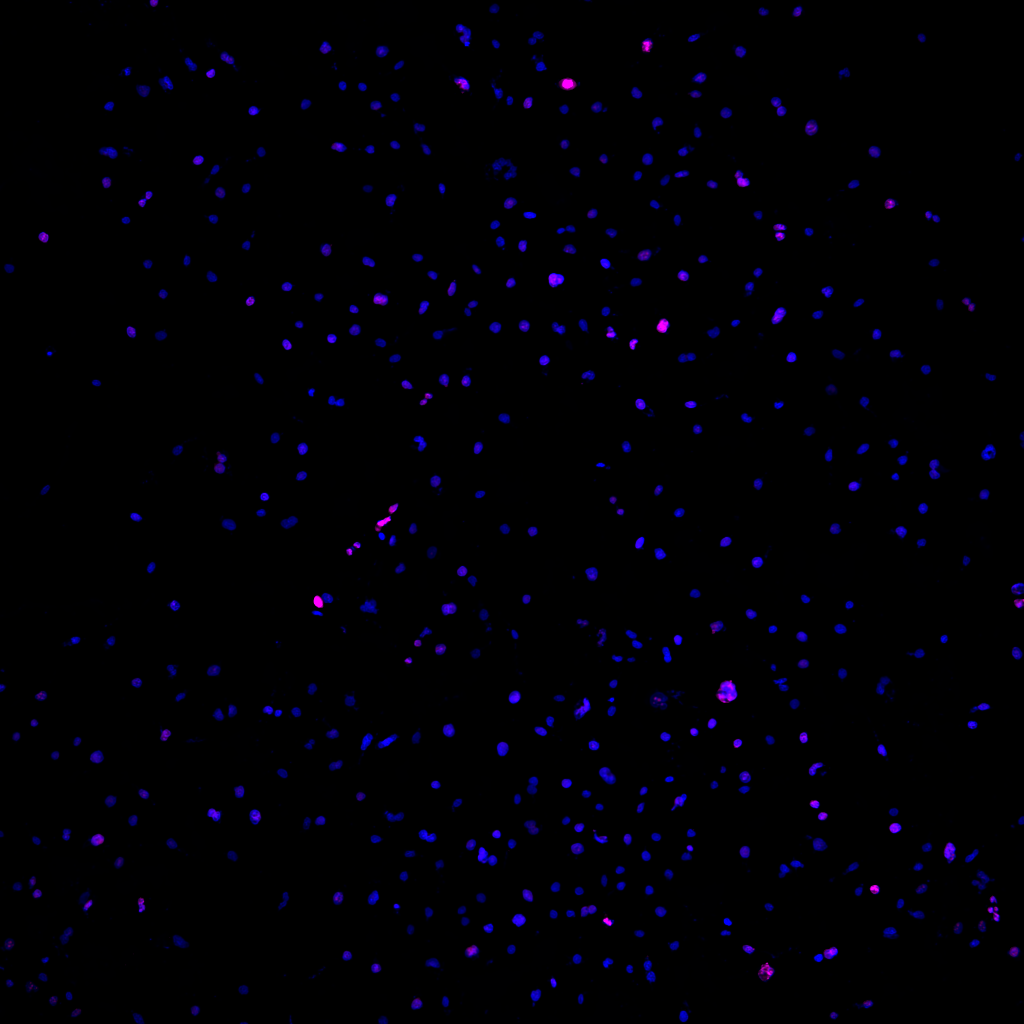

Supplement: Supplementary file 19 — Figure EV2 Source Data [file 44321_2025_260_MOESM19_ESM.zip › Figure EV2/EV2H/sh-LRP8-1#.png]

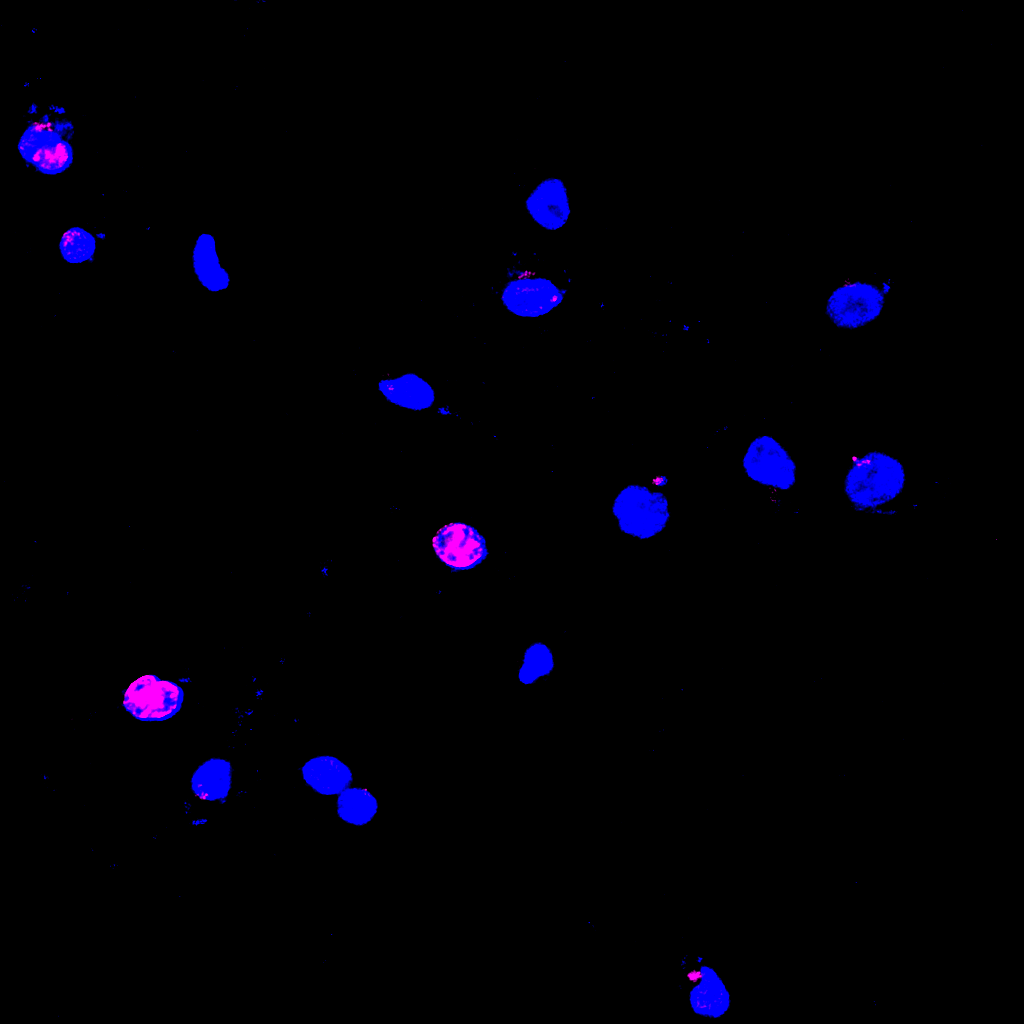

Supplement: Supplementary file 19 — Figure EV2 Source Data [file 44321_2025_260_MOESM19_ESM.zip › Figure EV2/EV2H/sh-LRP8-2# 6X.tif]

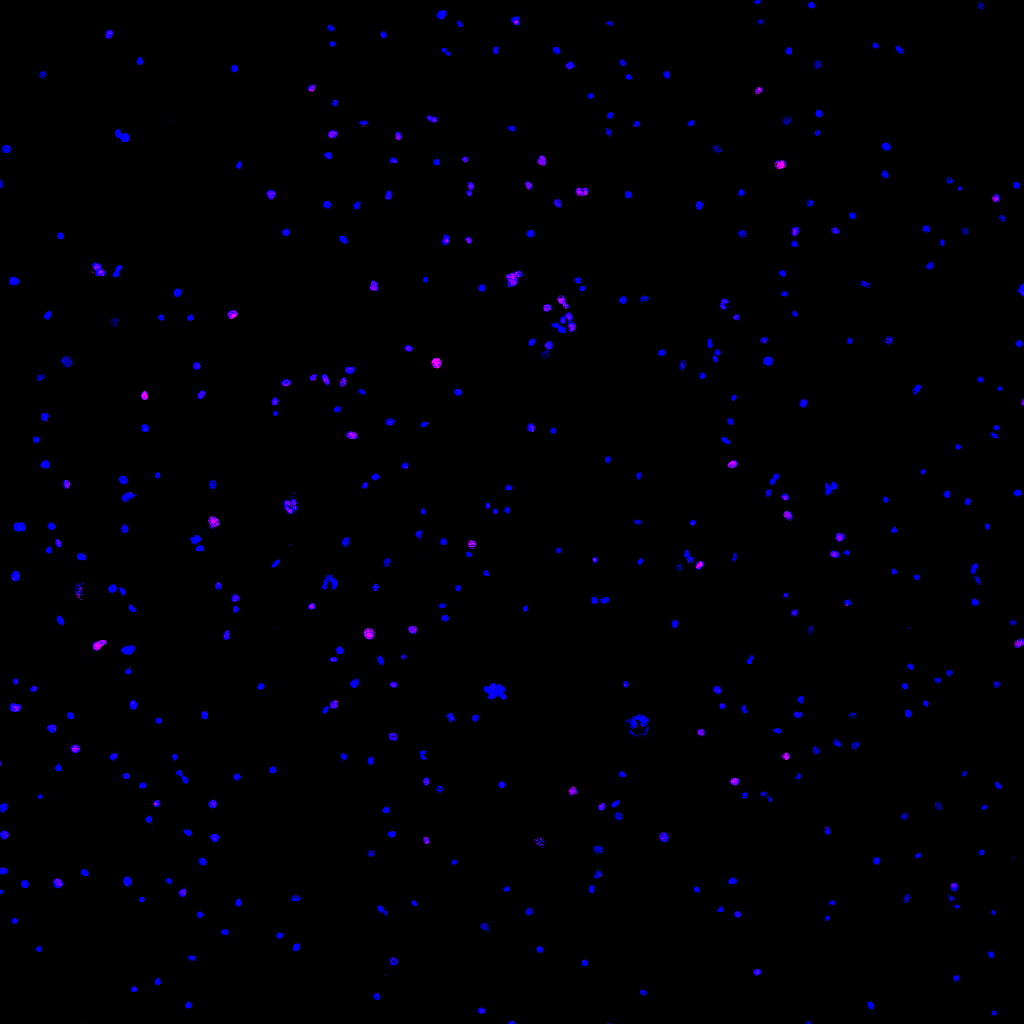

Supplement: Supplementary file 19 — Figure EV2 Source Data [file 44321_2025_260_MOESM19_ESM.zip › Figure EV2/EV2H/sh-LRP8-2#.tif]

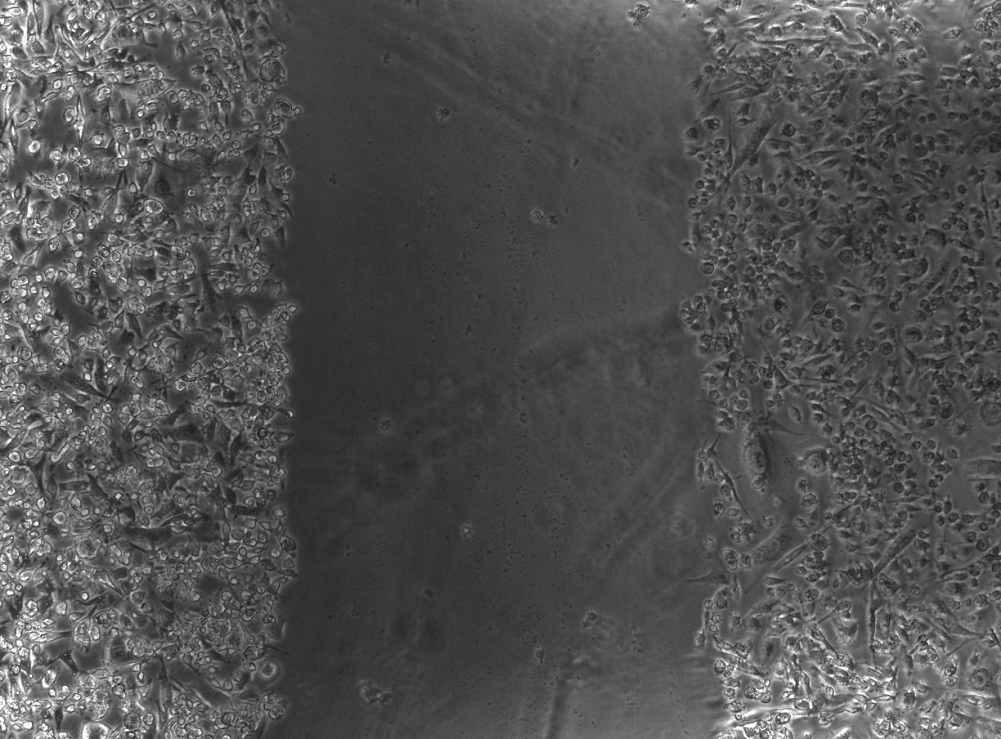

Supplement: Supplementary file 20 — Figure EV3 Source Data [file 44321_2025_260_MOESM20_ESM.zip › Figure EV3/EV3A/sh-ctrl 0h.png]

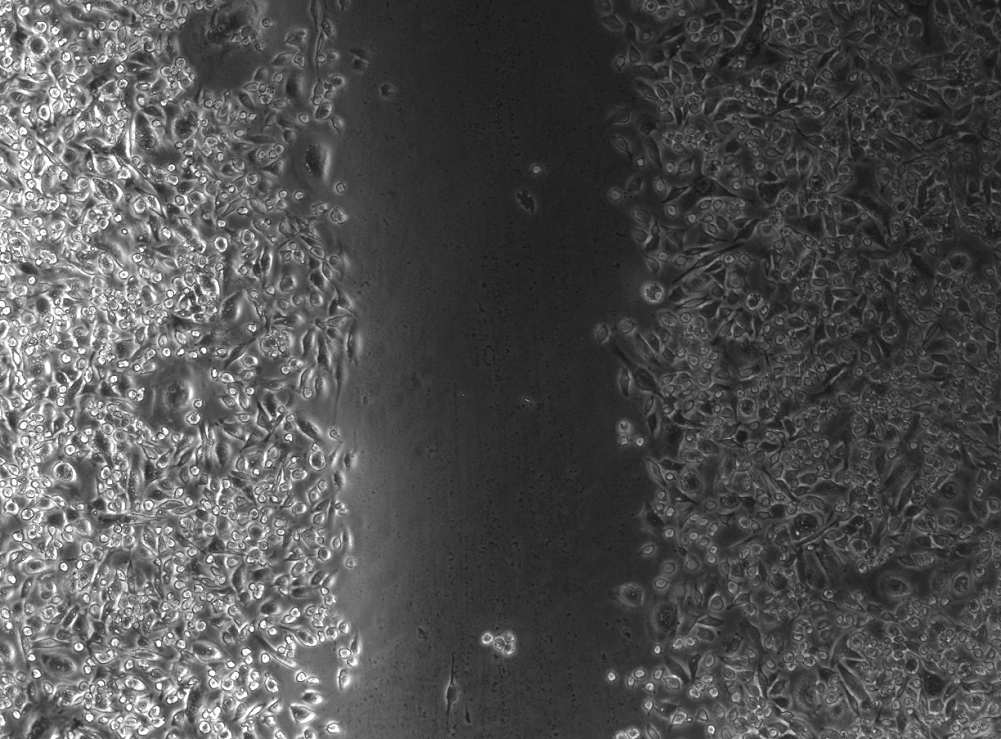

Supplement: Supplementary file 20 — Figure EV3 Source Data [file 44321_2025_260_MOESM20_ESM.zip › Figure EV3/EV3A/sh-ctrl 24h.png]

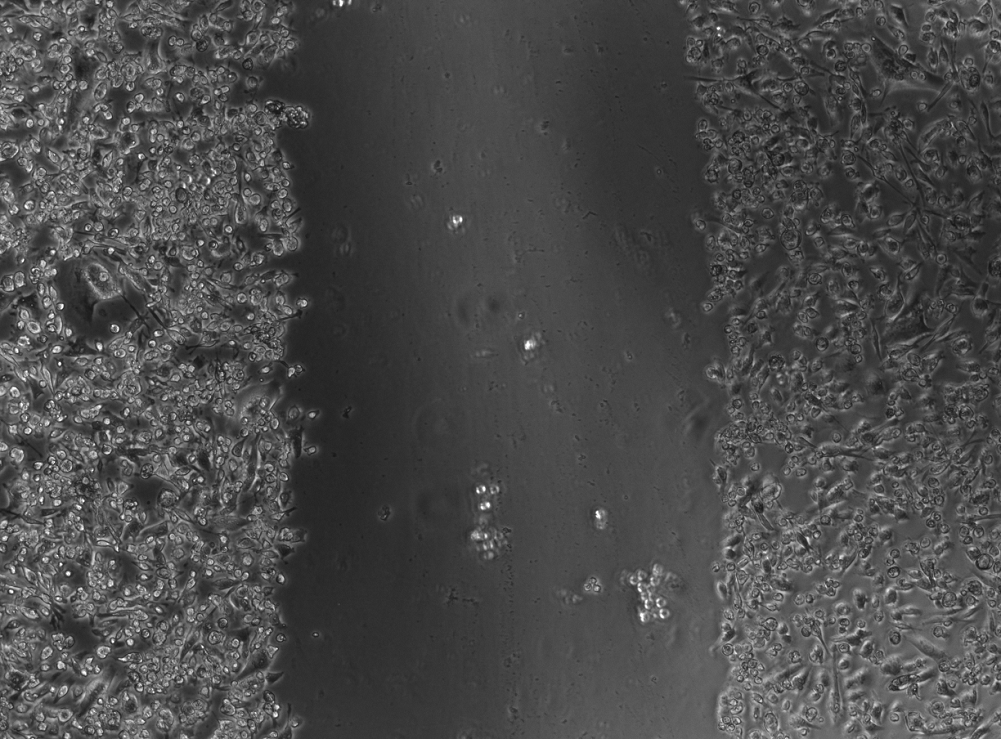

Supplement: Supplementary file 20 — Figure EV3 Source Data [file 44321_2025_260_MOESM20_ESM.zip › Figure EV3/EV3A/sh-LRP8-1# 0h.png]

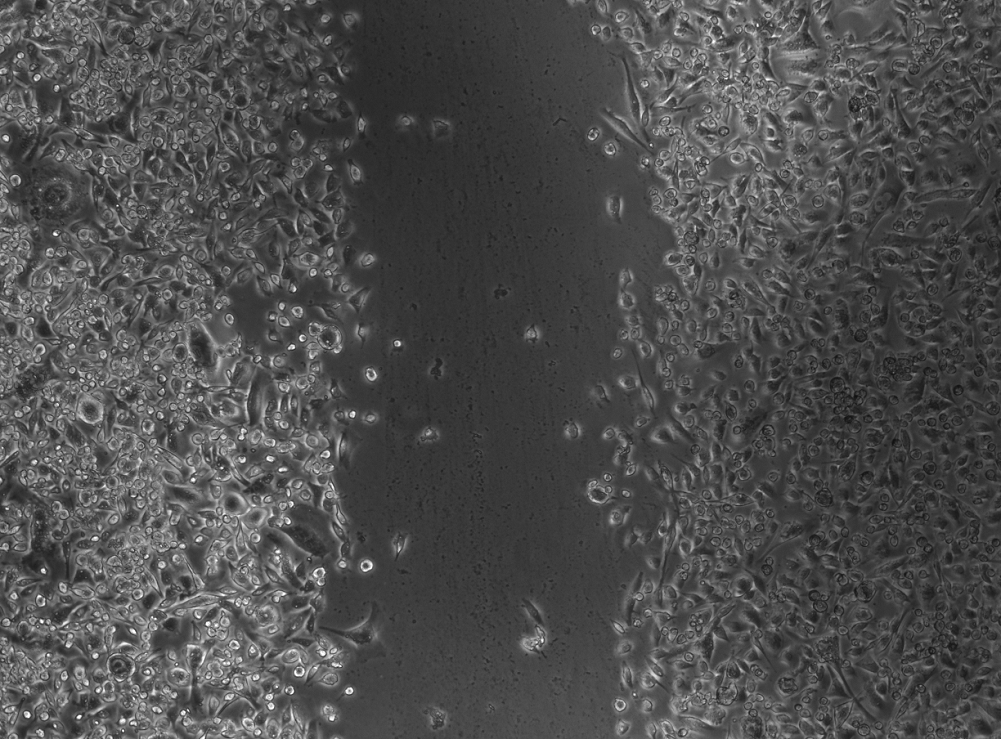

Supplement: Supplementary file 20 — Figure EV3 Source Data [file 44321_2025_260_MOESM20_ESM.zip › Figure EV3/EV3A/sh-LRP8-1# 24h.png]

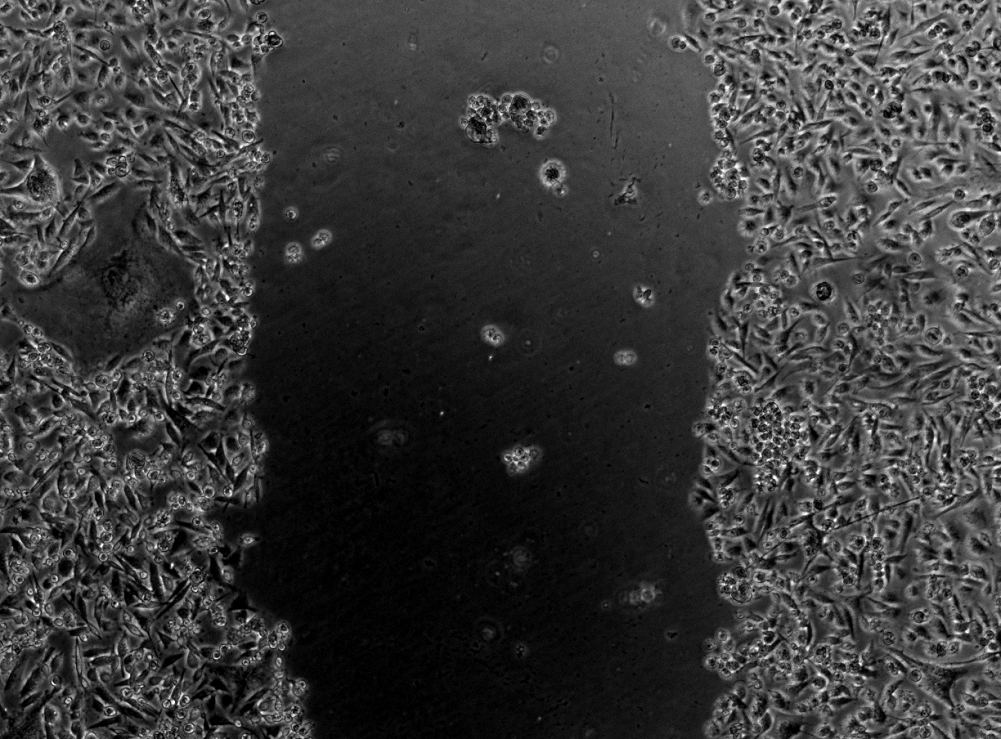

Supplement: Supplementary file 20 — Figure EV3 Source Data [file 44321_2025_260_MOESM20_ESM.zip › Figure EV3/EV3A/sh-LRP8-2# 0h.png]

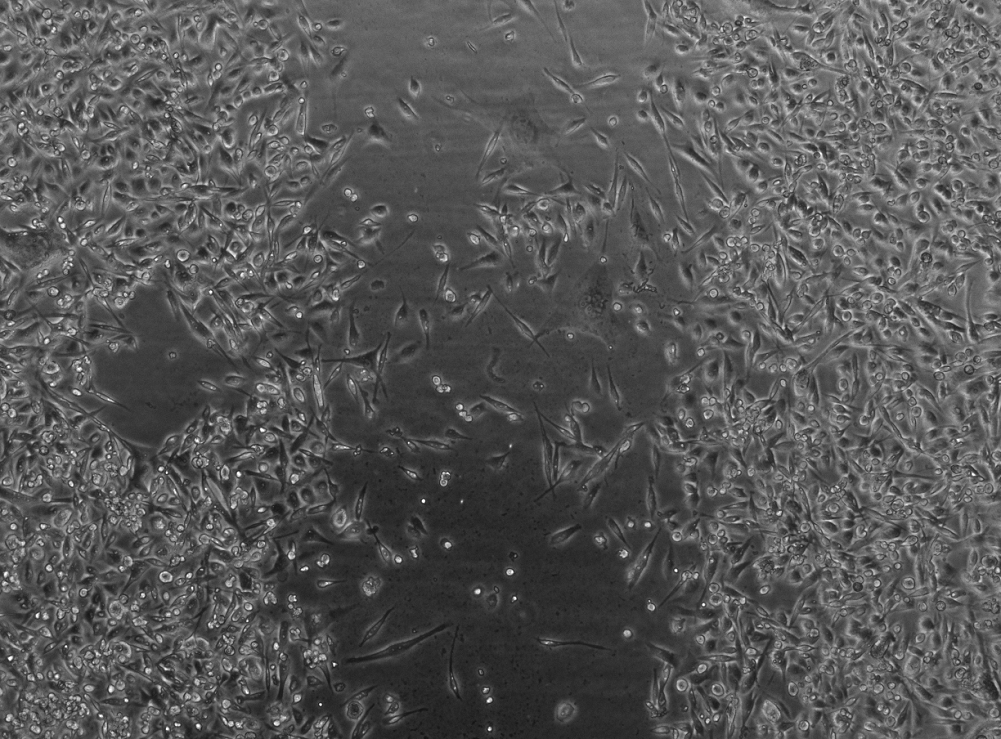

Supplement: Supplementary file 20 — Figure EV3 Source Data [file 44321_2025_260_MOESM20_ESM.zip › Figure EV3/EV3A/sh-LRP8-2# 24h.png]

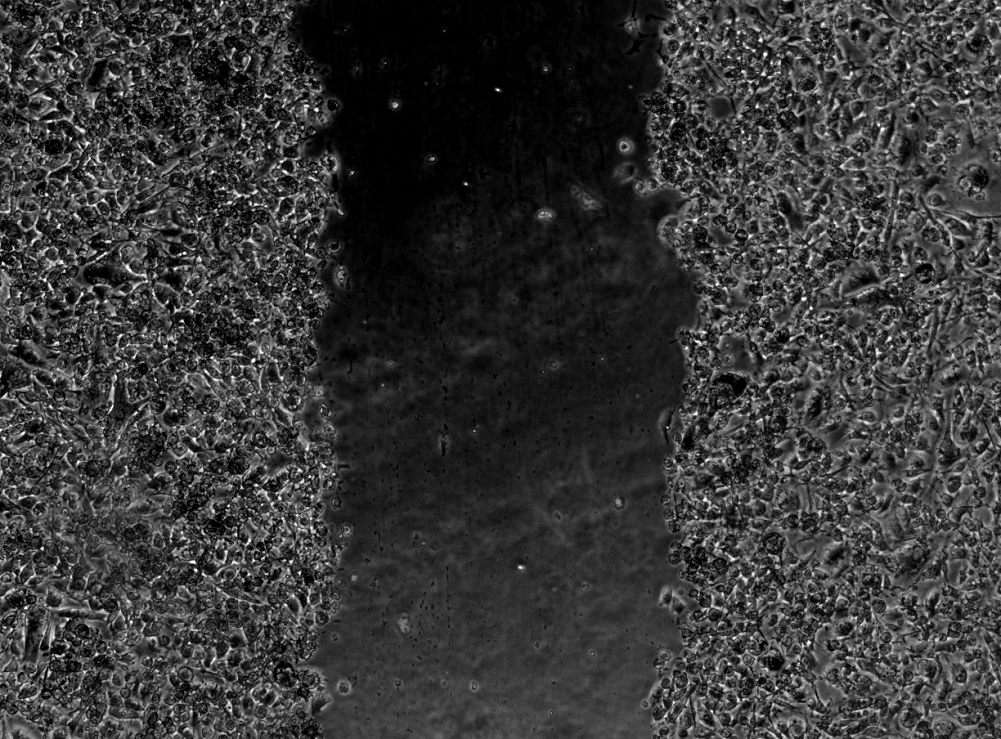

Supplement: Supplementary file 20 — Figure EV3 Source Data [file 44321_2025_260_MOESM20_ESM.zip › Figure EV3/EV3C/sh-ctrl 0h.png]

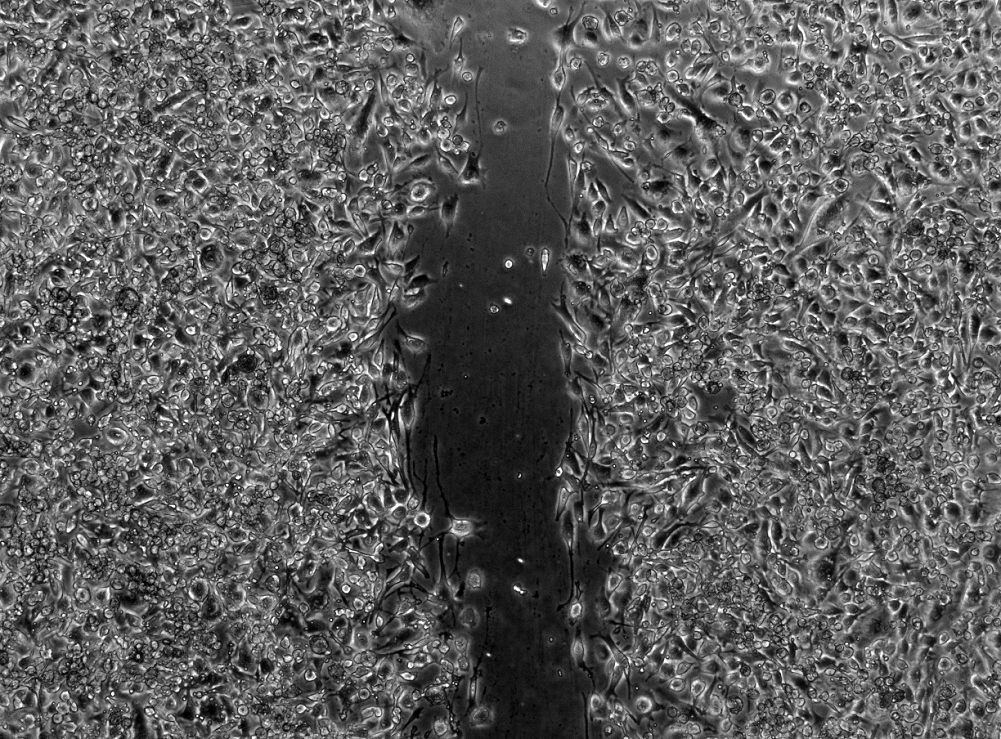

Supplement: Supplementary file 20 — Figure EV3 Source Data [file 44321_2025_260_MOESM20_ESM.zip › Figure EV3/EV3C/sh-ctrl 24h.png]

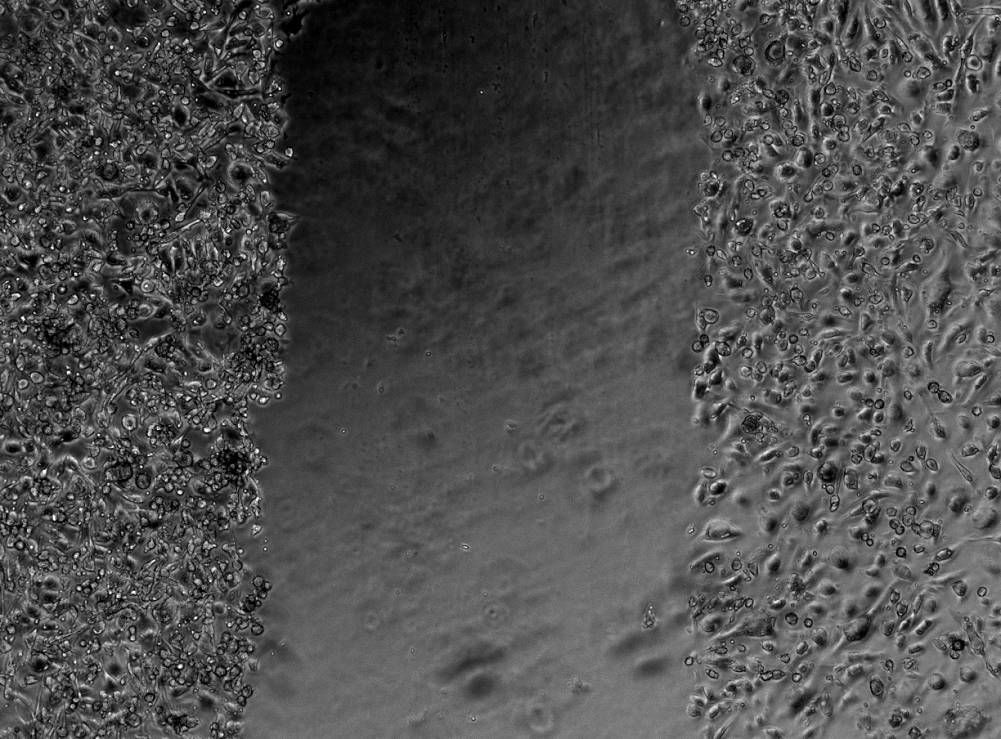

Supplement: Supplementary file 20 — Figure EV3 Source Data [file 44321_2025_260_MOESM20_ESM.zip › Figure EV3/EV3C/sh-LRP8-1# 0h.png]

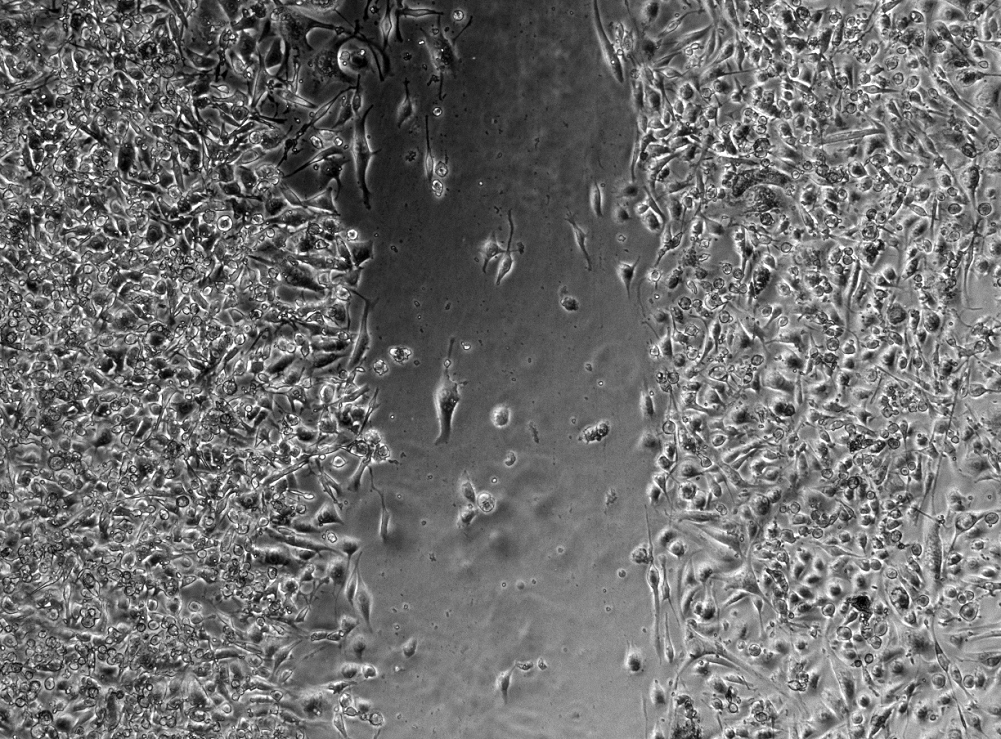

Supplement: Supplementary file 20 — Figure EV3 Source Data [file 44321_2025_260_MOESM20_ESM.zip › Figure EV3/EV3C/sh-LRP8-1# 24h.png]

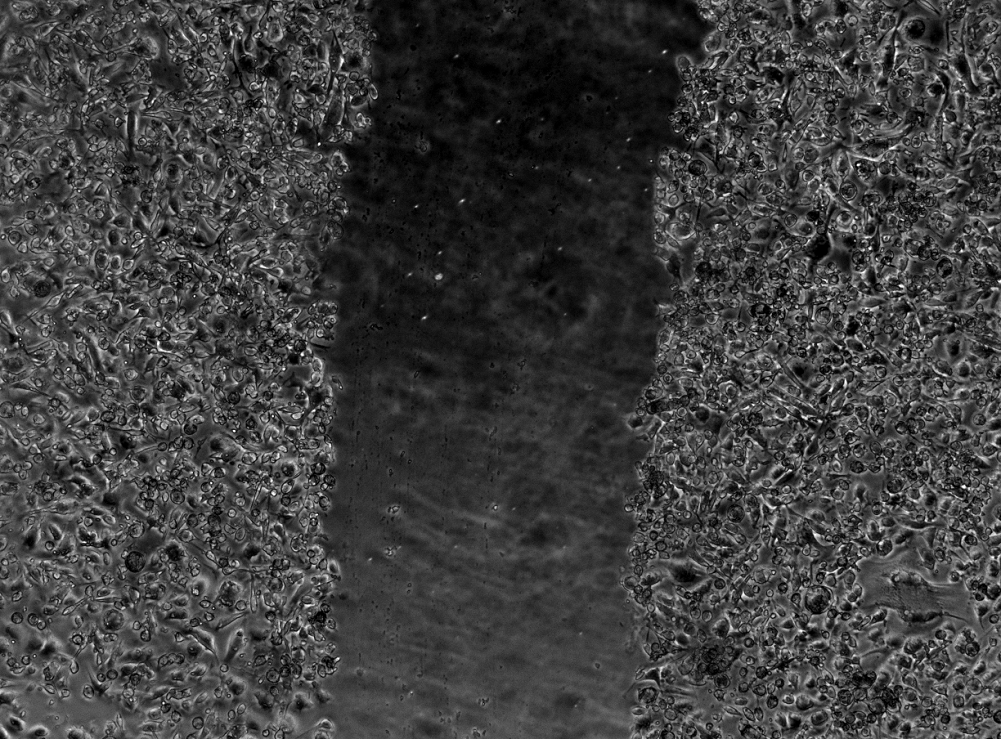

Supplement: Supplementary file 20 — Figure EV3 Source Data [file 44321_2025_260_MOESM20_ESM.zip › Figure EV3/EV3C/sh-LRP8-2# 0h.png]

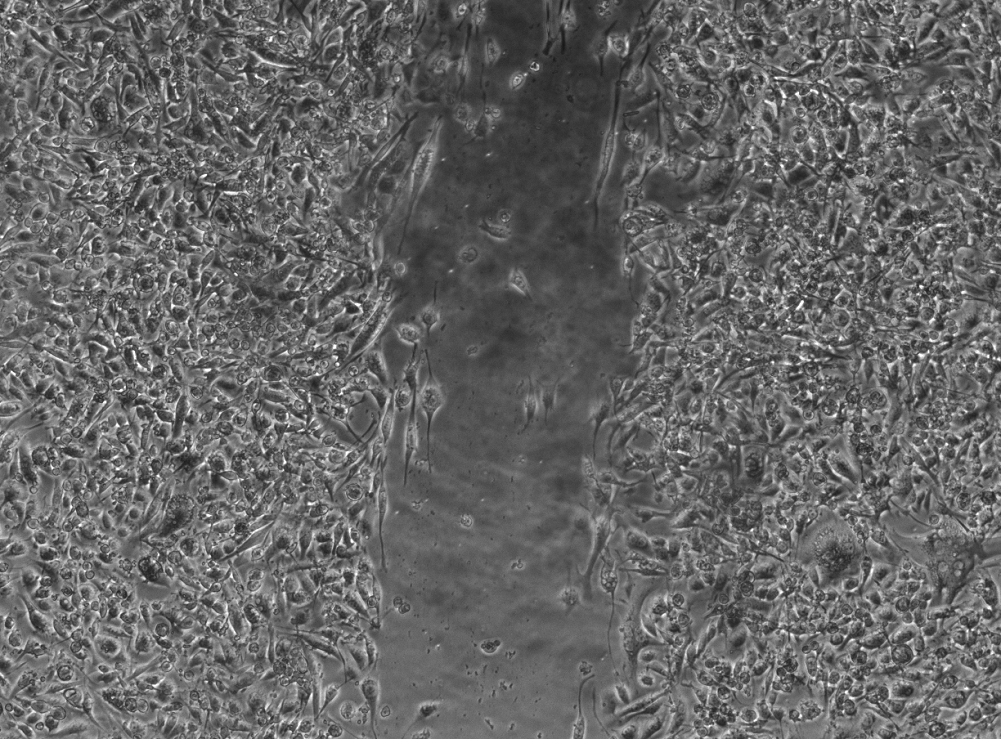

Supplement: Supplementary file 20 — Figure EV3 Source Data [file 44321_2025_260_MOESM20_ESM.zip › Figure EV3/EV3C/sh-LRP8-2# 24h.png]

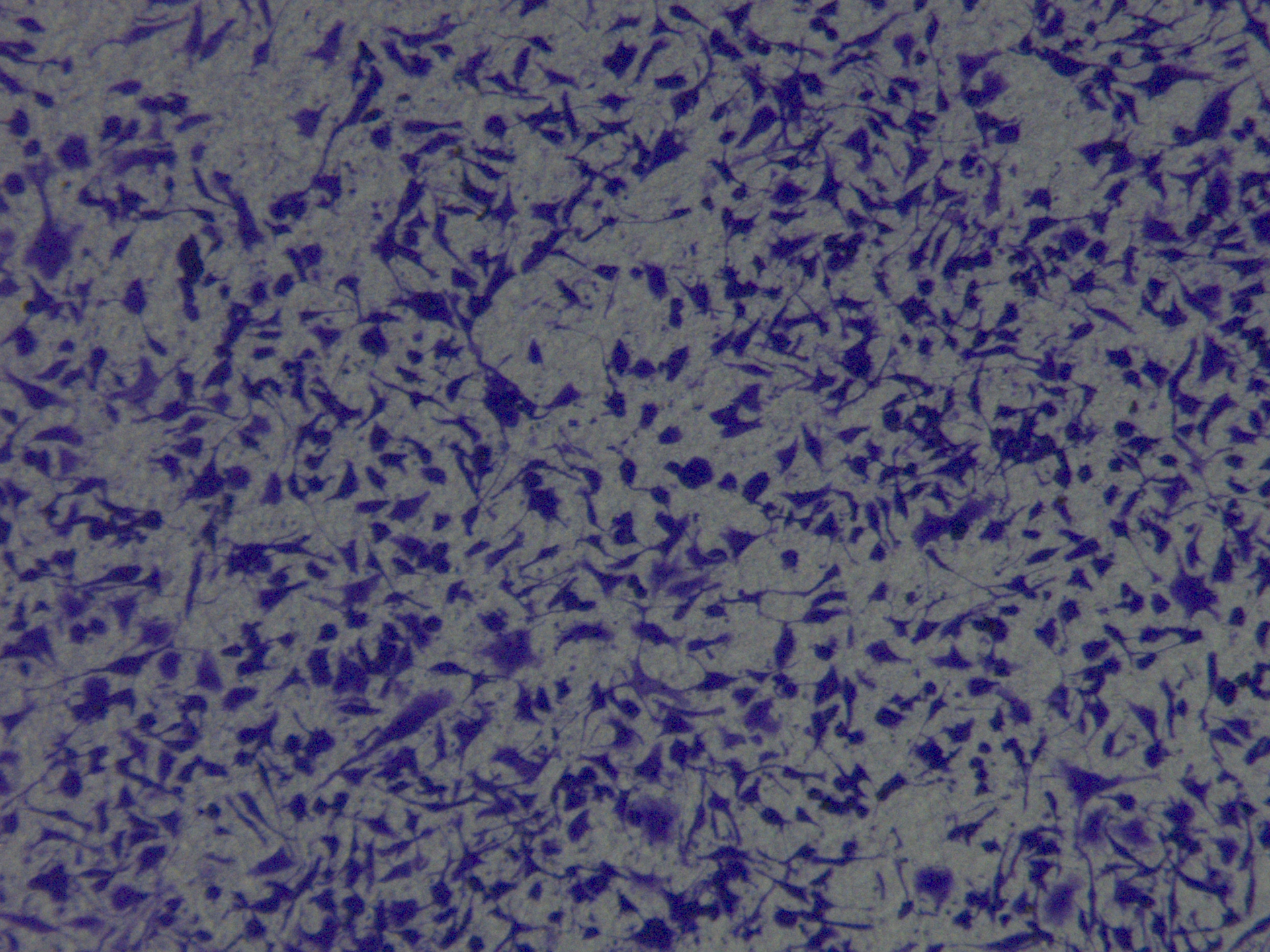

Supplement: Supplementary file 20 — Figure EV3 Source Data [file 44321_2025_260_MOESM20_ESM.zip › Figure EV3/EV3E/sh-ctrl invasion.tif]

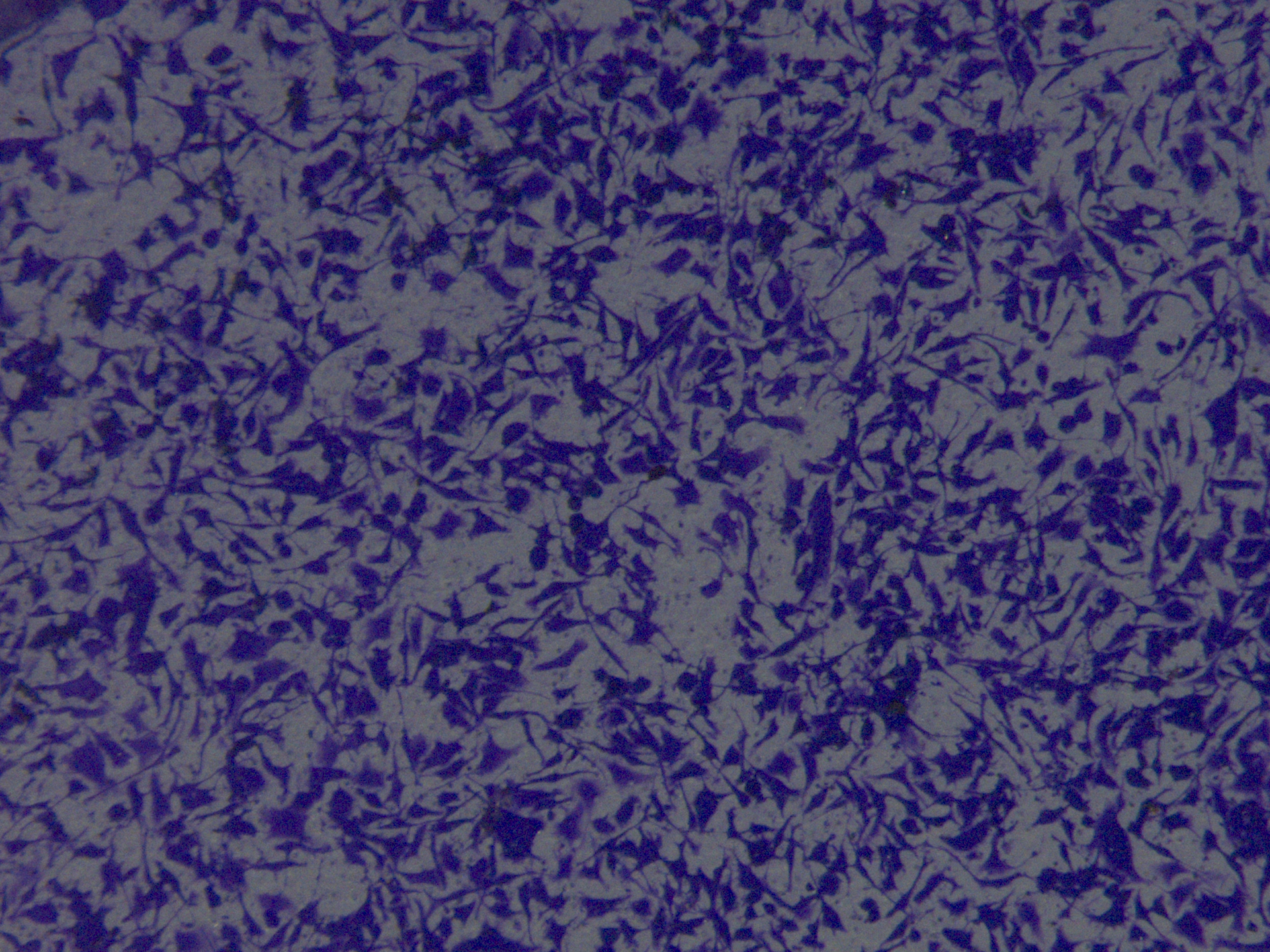

Supplement: Supplementary file 20 — Figure EV3 Source Data [file 44321_2025_260_MOESM20_ESM.zip › Figure EV3/EV3E/sh-ctrl migration.tif]

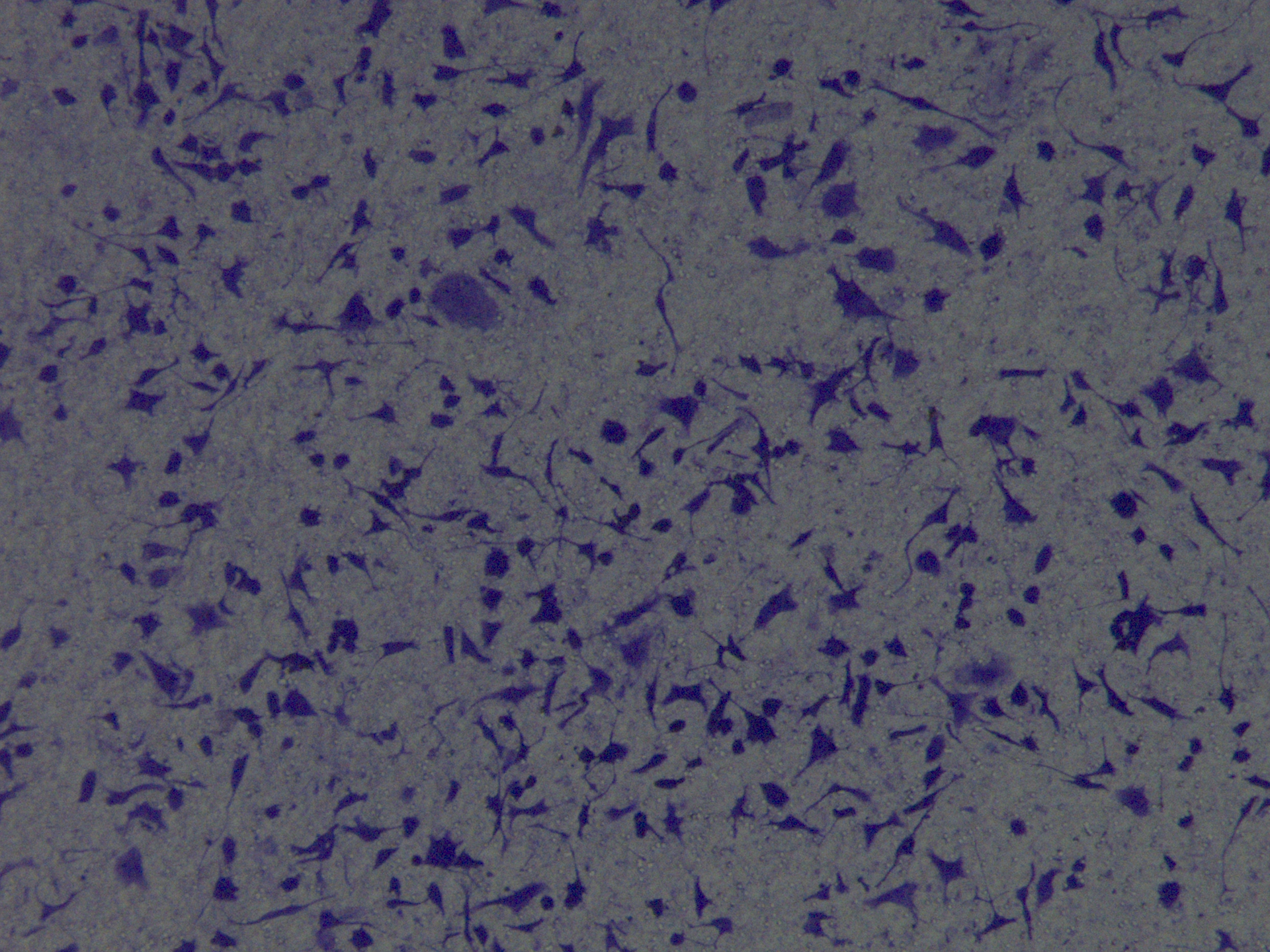

Supplement: Supplementary file 20 — Figure EV3 Source Data [file 44321_2025_260_MOESM20_ESM.zip › Figure EV3/EV3E/sh-LRP8-1# invasion.tif]

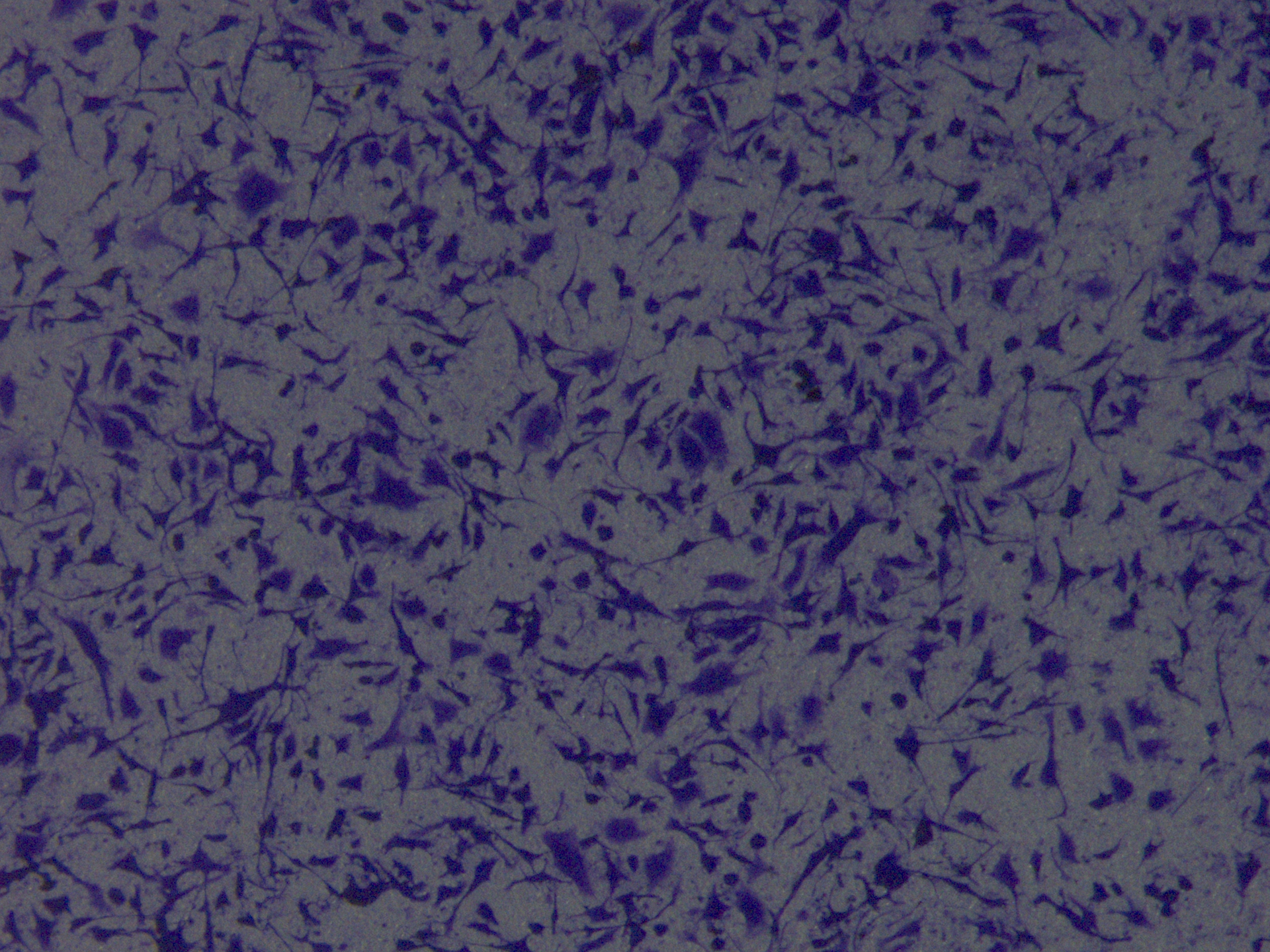

Supplement: Supplementary file 20 — Figure EV3 Source Data [file 44321_2025_260_MOESM20_ESM.zip › Figure EV3/EV3E/sh-LRP8-1# migration.tif]

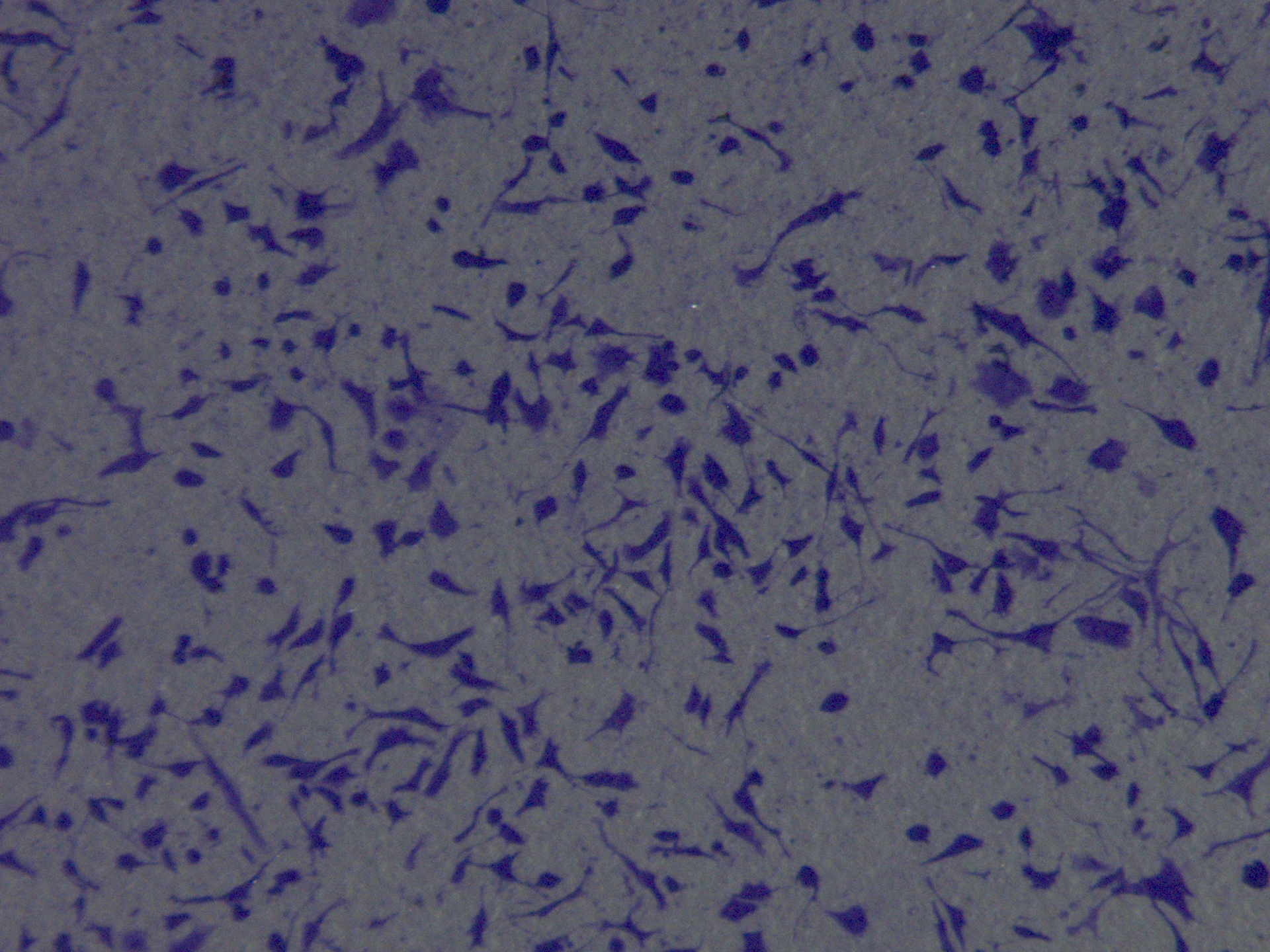

Supplement: Supplementary file 20 — Figure EV3 Source Data [file 44321_2025_260_MOESM20_ESM.zip › Figure EV3/EV3E/sh-LRP8-2# invasion.tif]

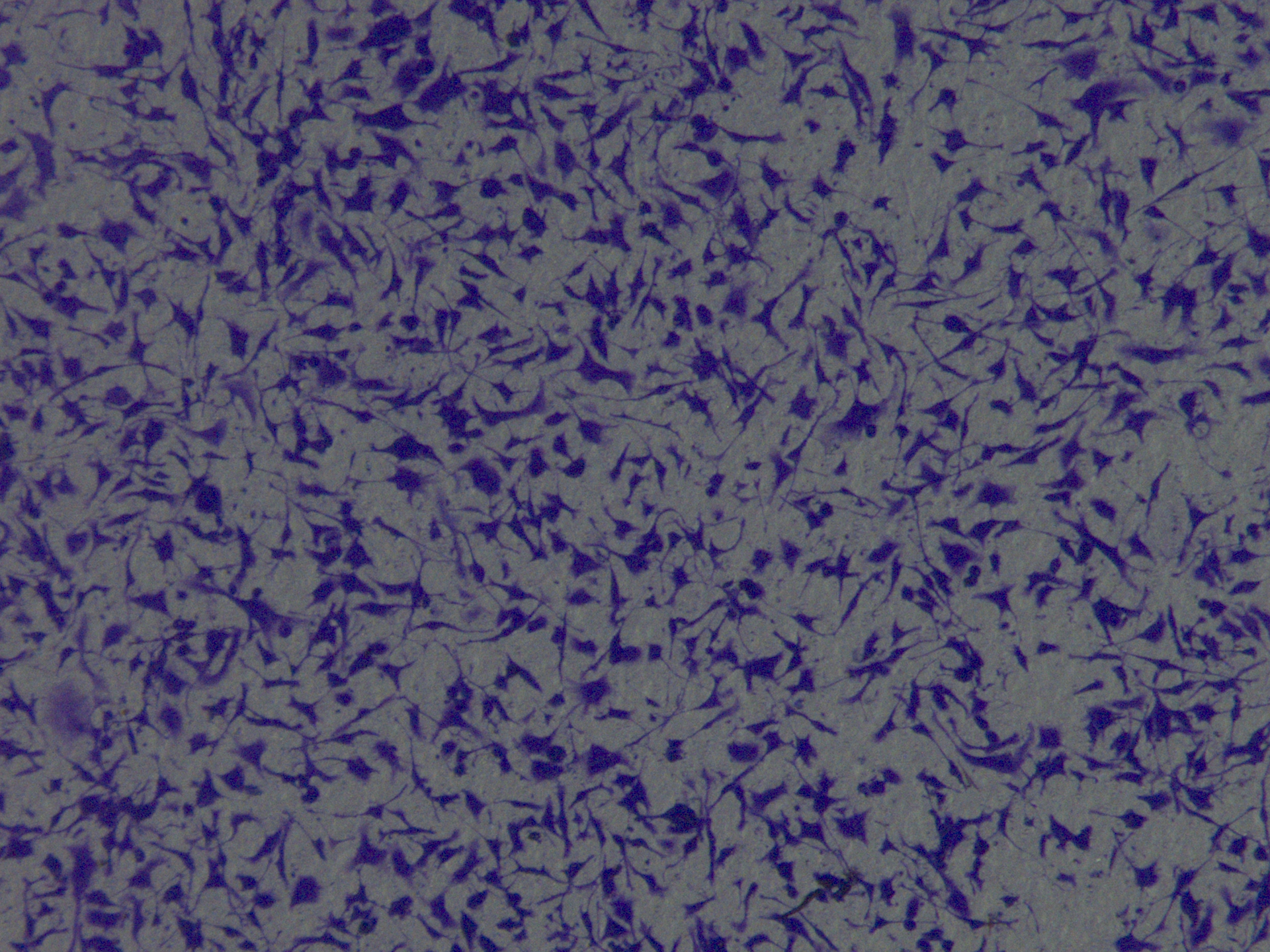

Supplement: Supplementary file 20 — Figure EV3 Source Data [file 44321_2025_260_MOESM20_ESM.zip › Figure EV3/EV3E/sh-LRP8-2# migration.tif]

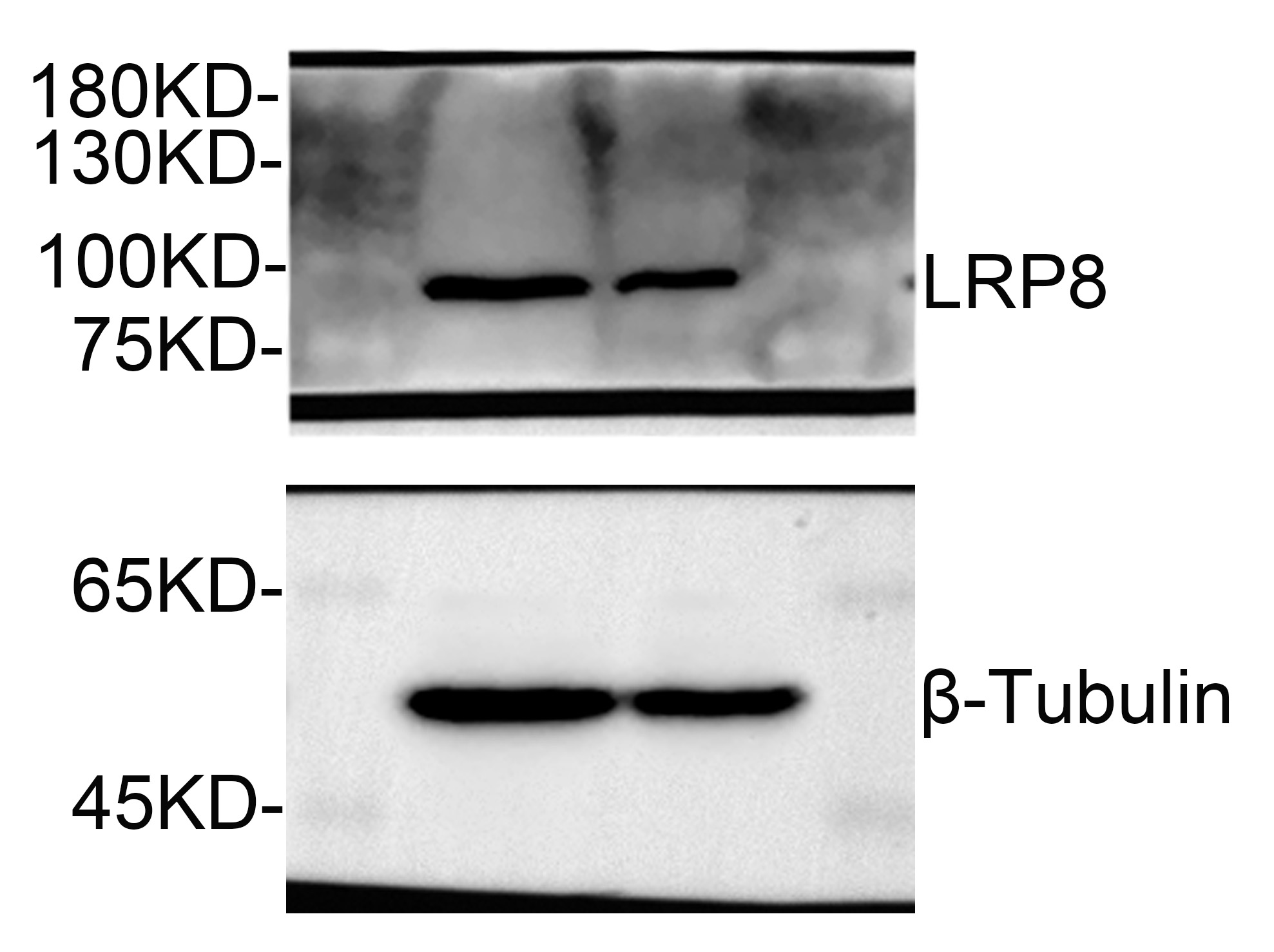

Supplement: Supplementary file 21 — Figure EV4 Source Data [file 44321_2025_260_MOESM21_ESM.zip › Figure EV4/EV4A/EV4A Blot.jpg]

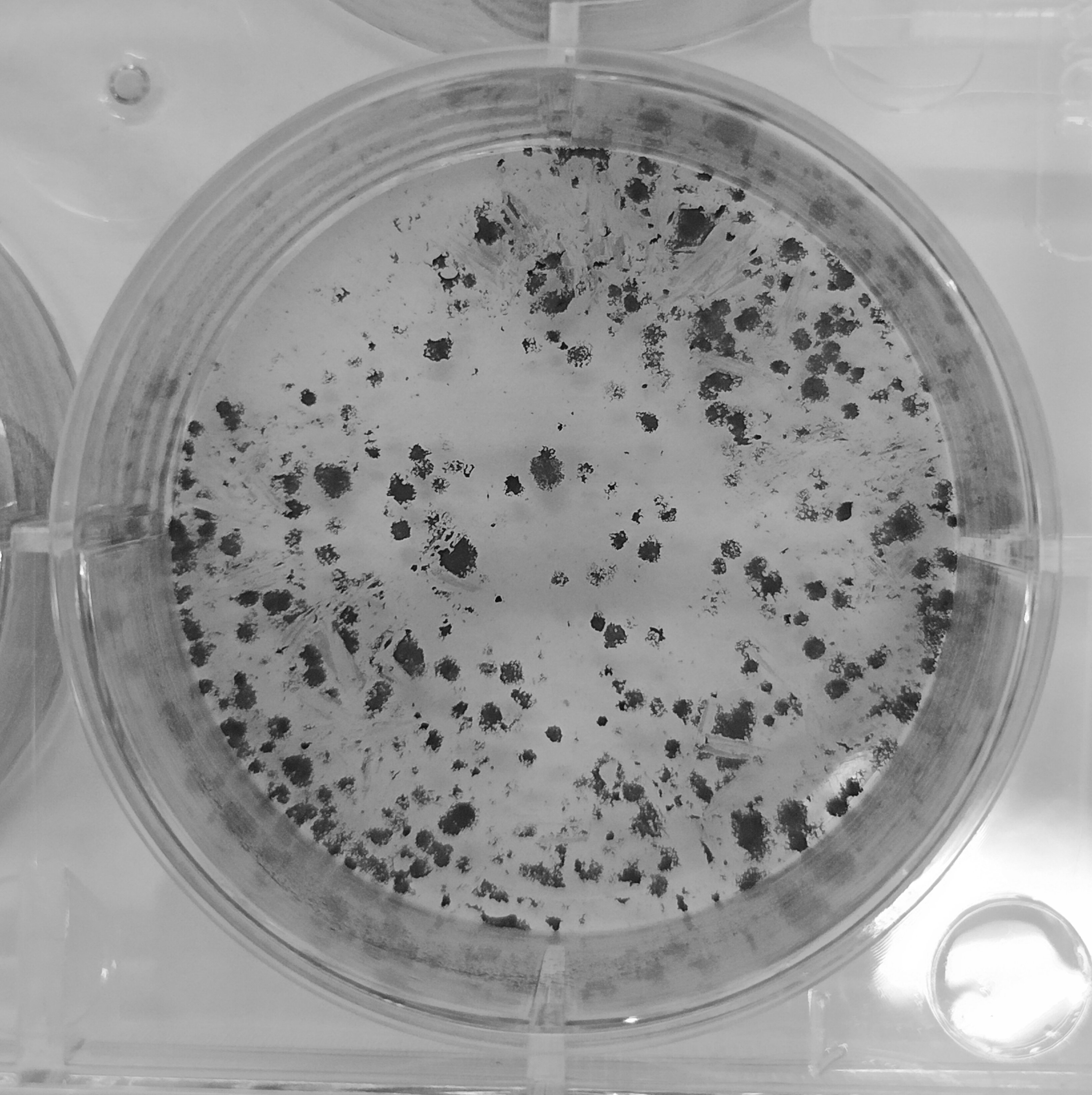

Supplement: Supplementary file 21 — Figure EV4 Source Data [file 44321_2025_260_MOESM21_ESM.zip › Figure EV4/EV4C/sh-ctrl vehicle..jpg]

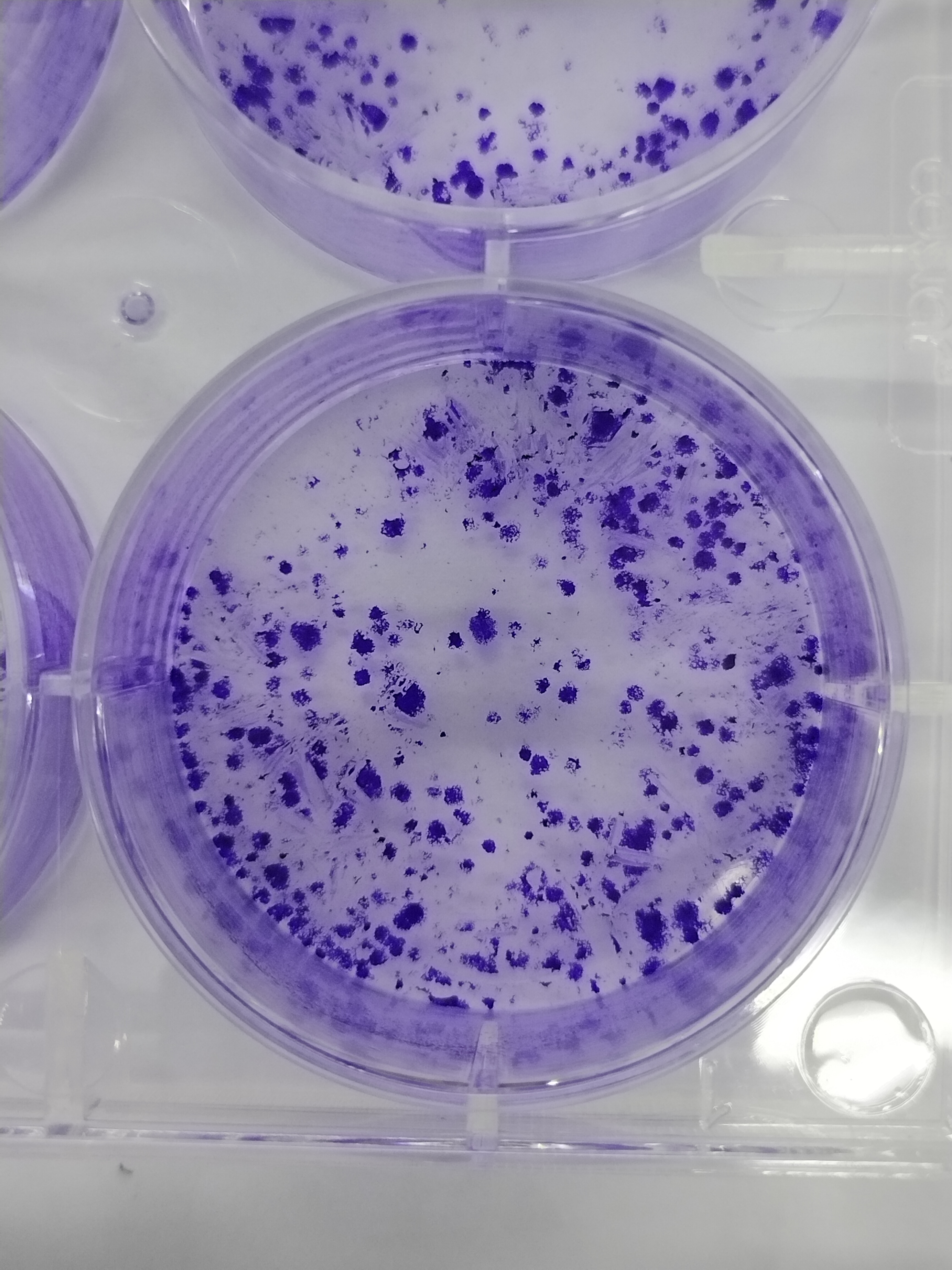

Supplement: Supplementary file 21 — Figure EV4 Source Data [file 44321_2025_260_MOESM21_ESM.zip › Figure EV4/EV4C/sh-ctrl vehicle.jpg]

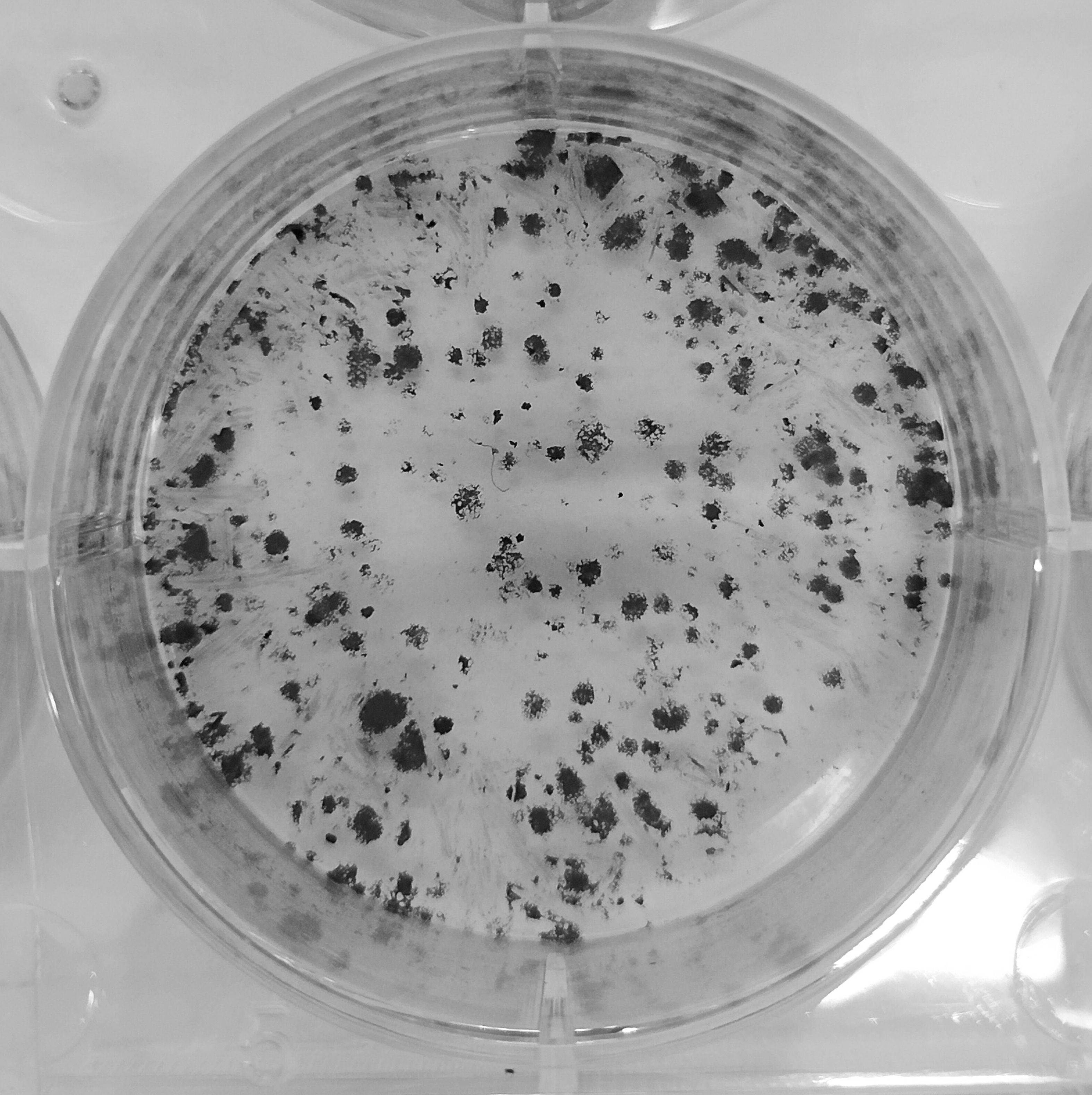

Supplement: Supplementary file 21 — Figure EV4 Source Data [file 44321_2025_260_MOESM21_ESM.zip › Figure EV4/EV4C/sh-ctrl+Reelin..jpg]

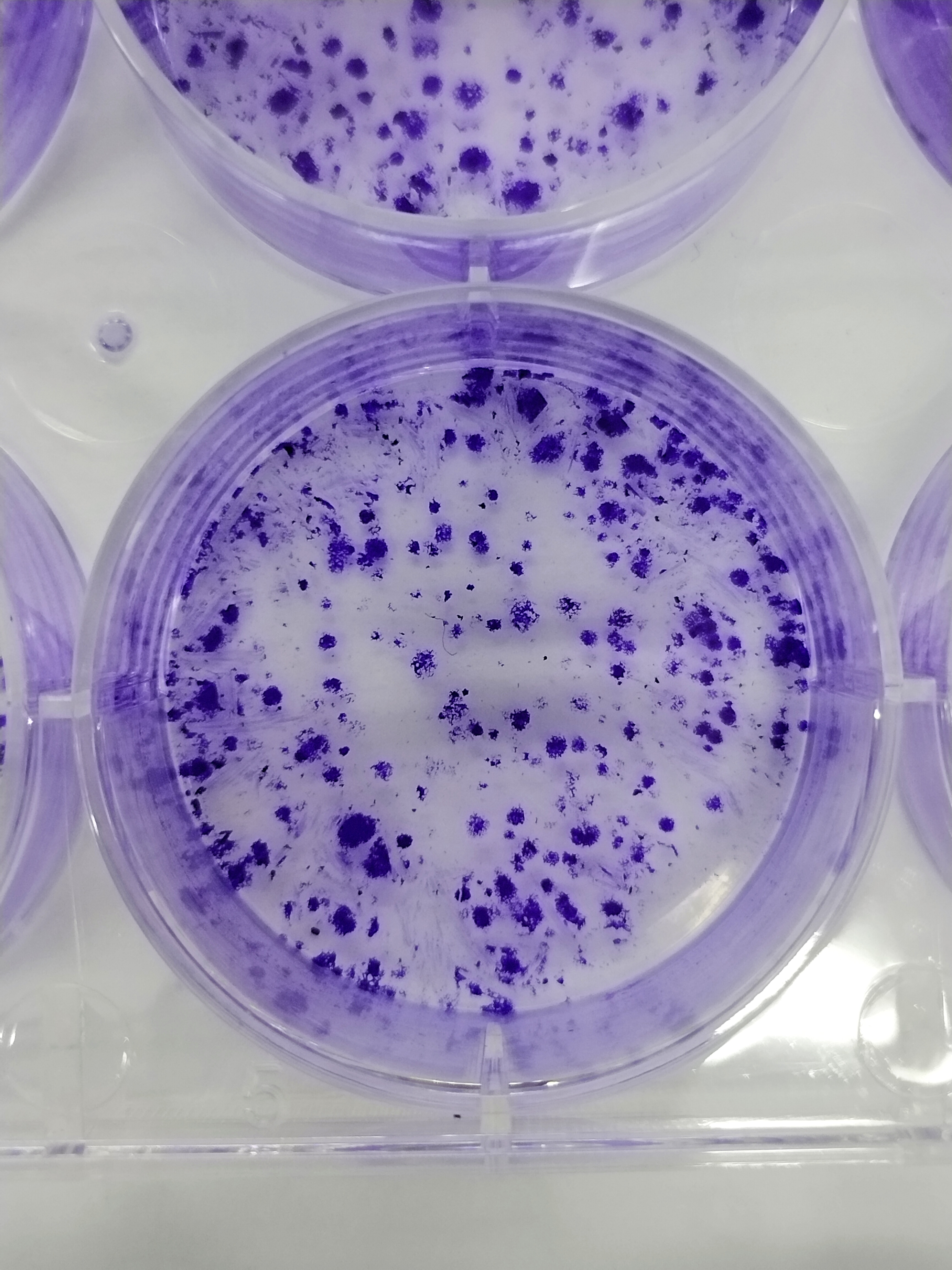

Supplement: Supplementary file 21 — Figure EV4 Source Data [file 44321_2025_260_MOESM21_ESM.zip › Figure EV4/EV4C/sh-ctrl+Reelin.jpg]

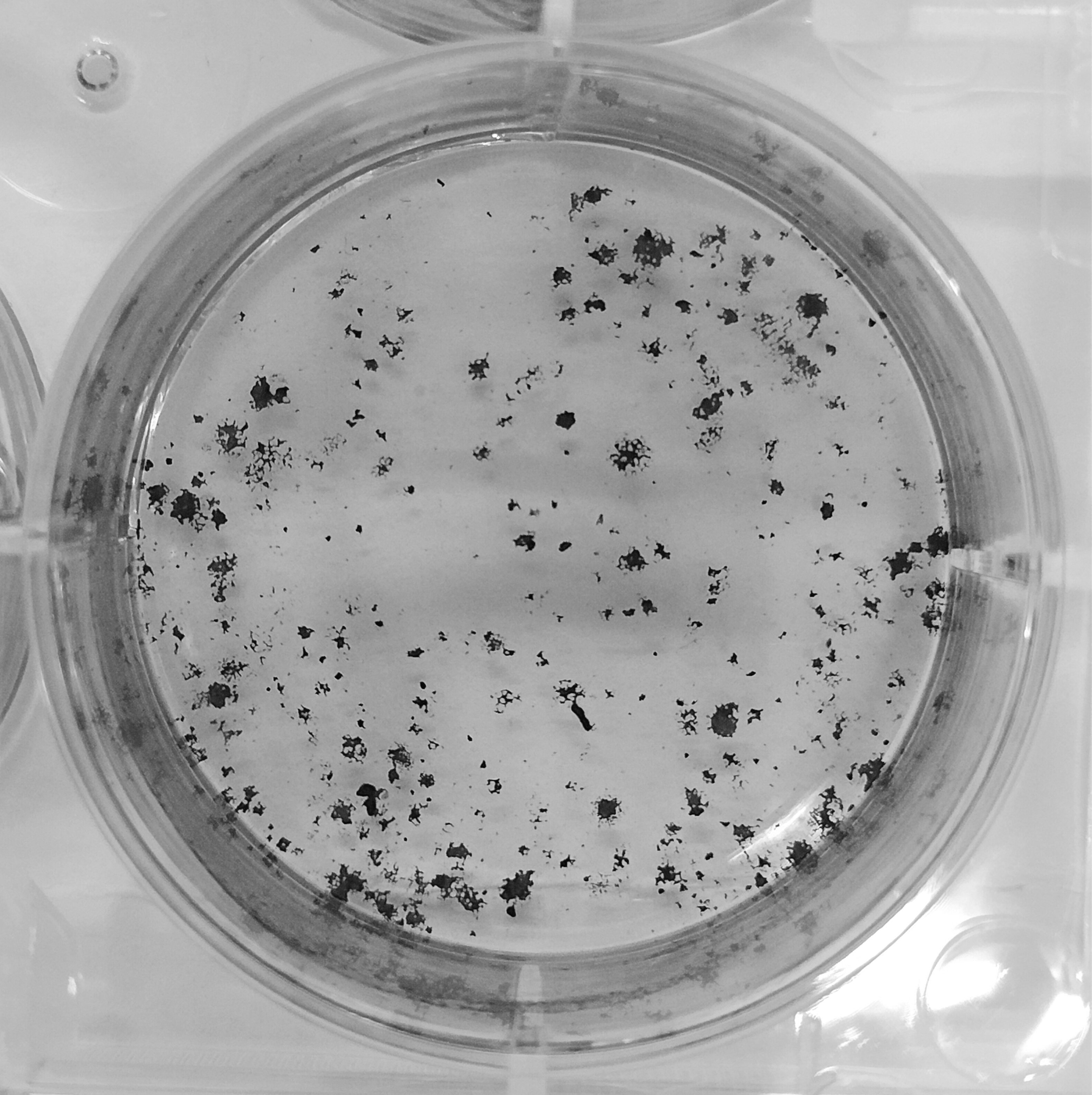

Supplement: Supplementary file 21 — Figure EV4 Source Data [file 44321_2025_260_MOESM21_ESM.zip › Figure EV4/EV4C/sh-LRP8-2# vehicle..jpg]

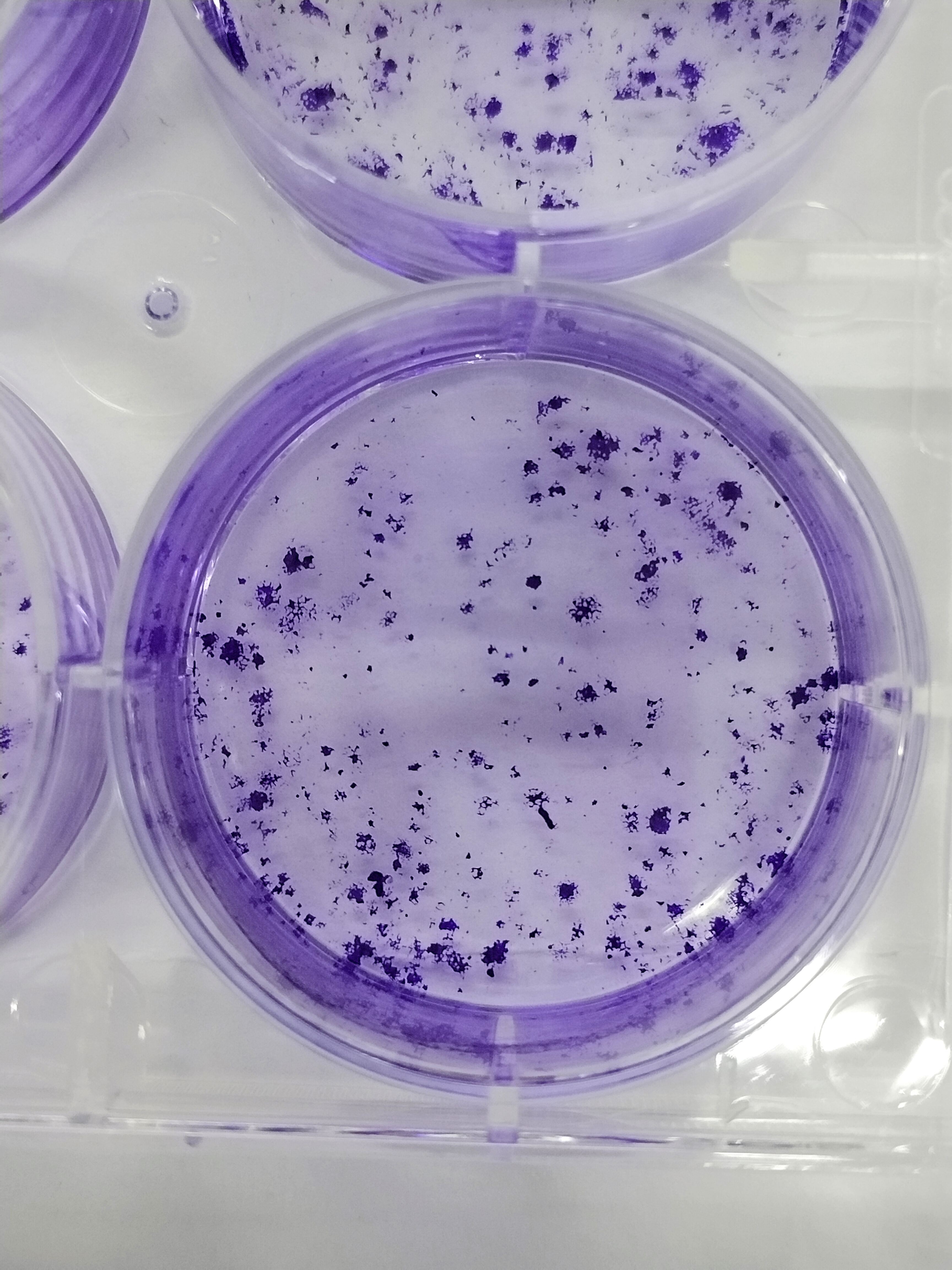

Supplement: Supplementary file 21 — Figure EV4 Source Data [file 44321_2025_260_MOESM21_ESM.zip › Figure EV4/EV4C/sh-LRP8-2# vehicle.jpg]

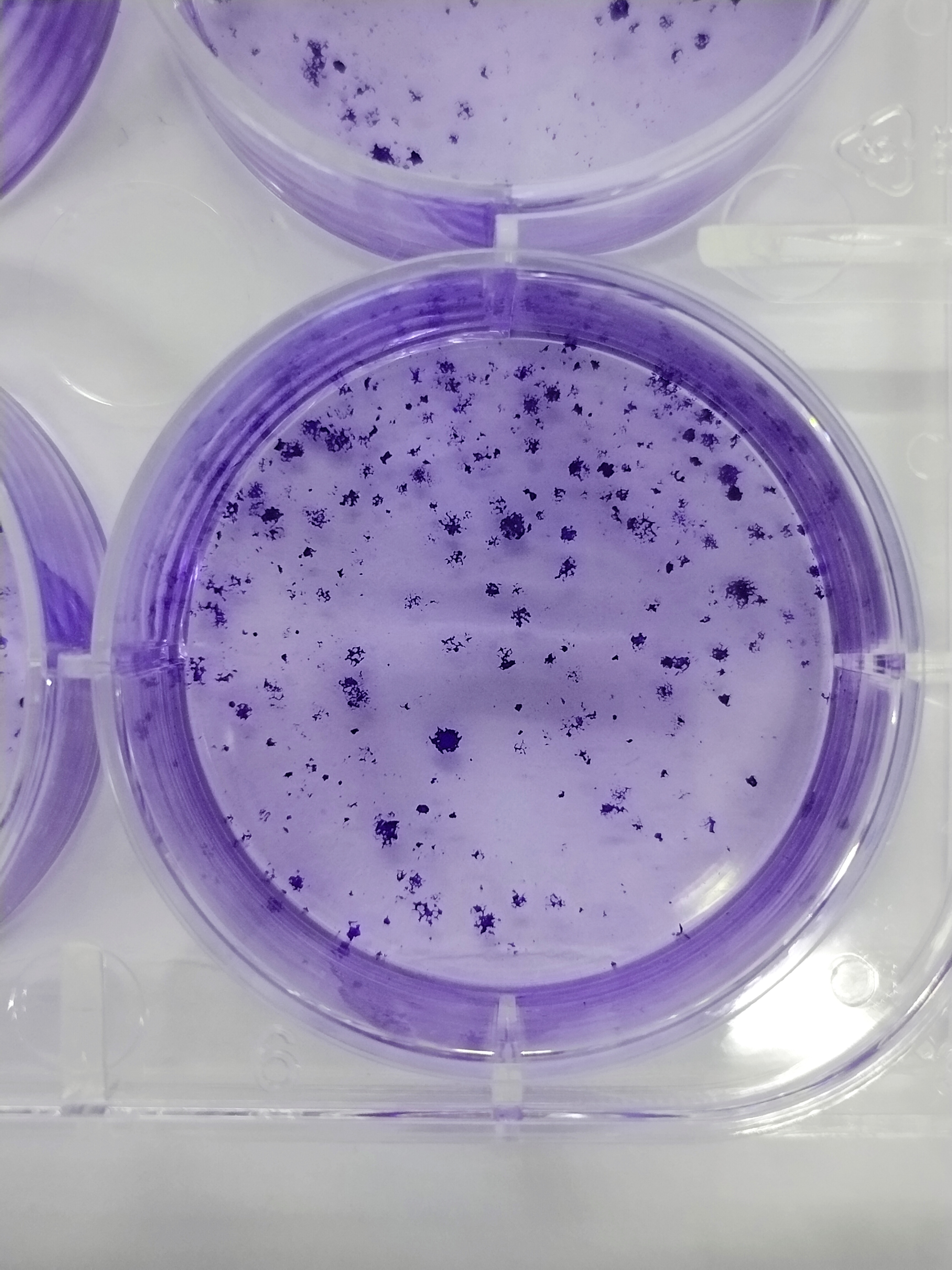

Supplement: Supplementary file 21 — Figure EV4 Source Data [file 44321_2025_260_MOESM21_ESM.zip › Figure EV4/EV4C/sh-LRP8-2#+Reelin..jpg]

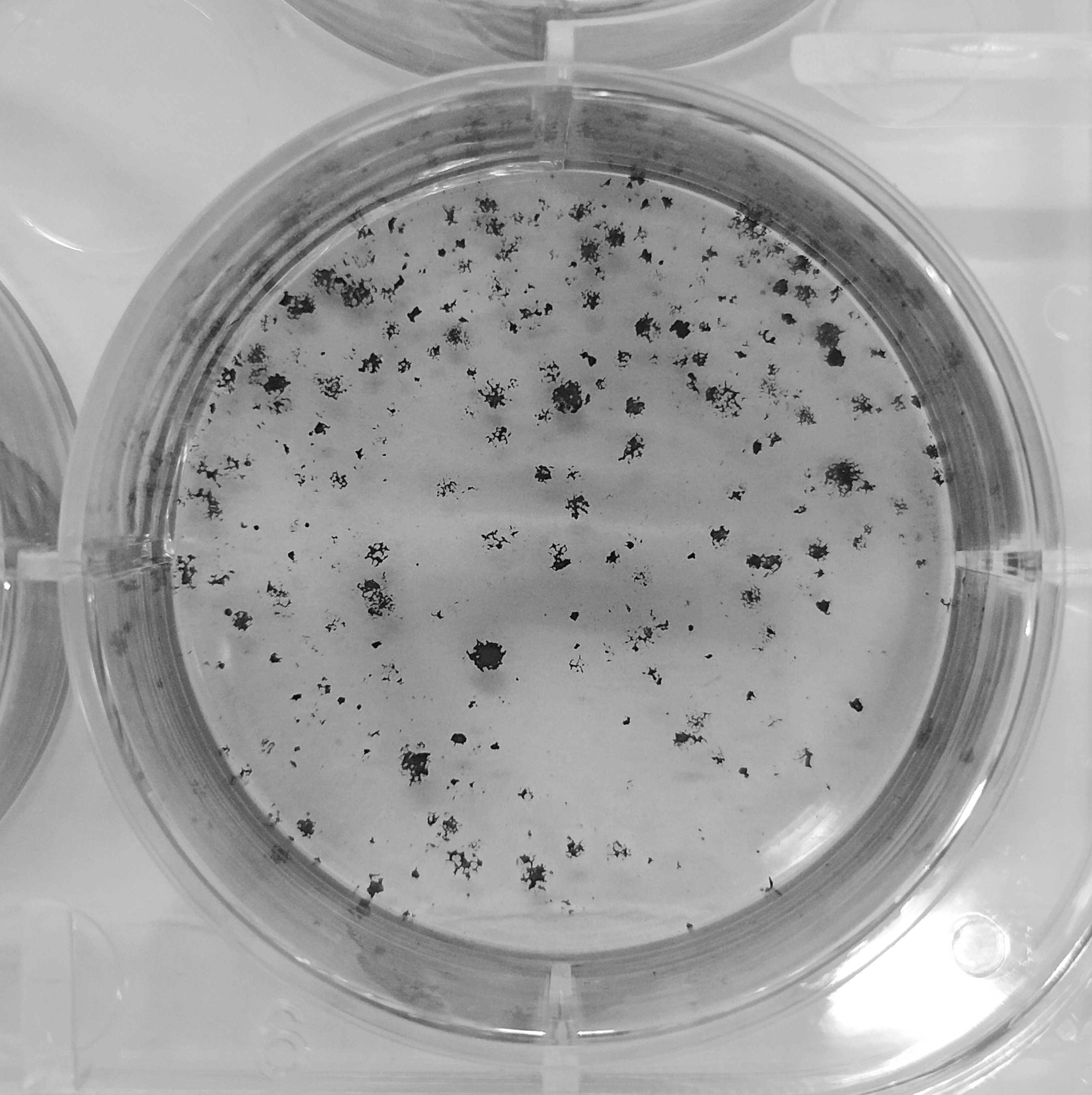

Supplement: Supplementary file 21 — Figure EV4 Source Data [file 44321_2025_260_MOESM21_ESM.zip › Figure EV4/EV4C/sh-LRP8-2#+Reelin.jpg]

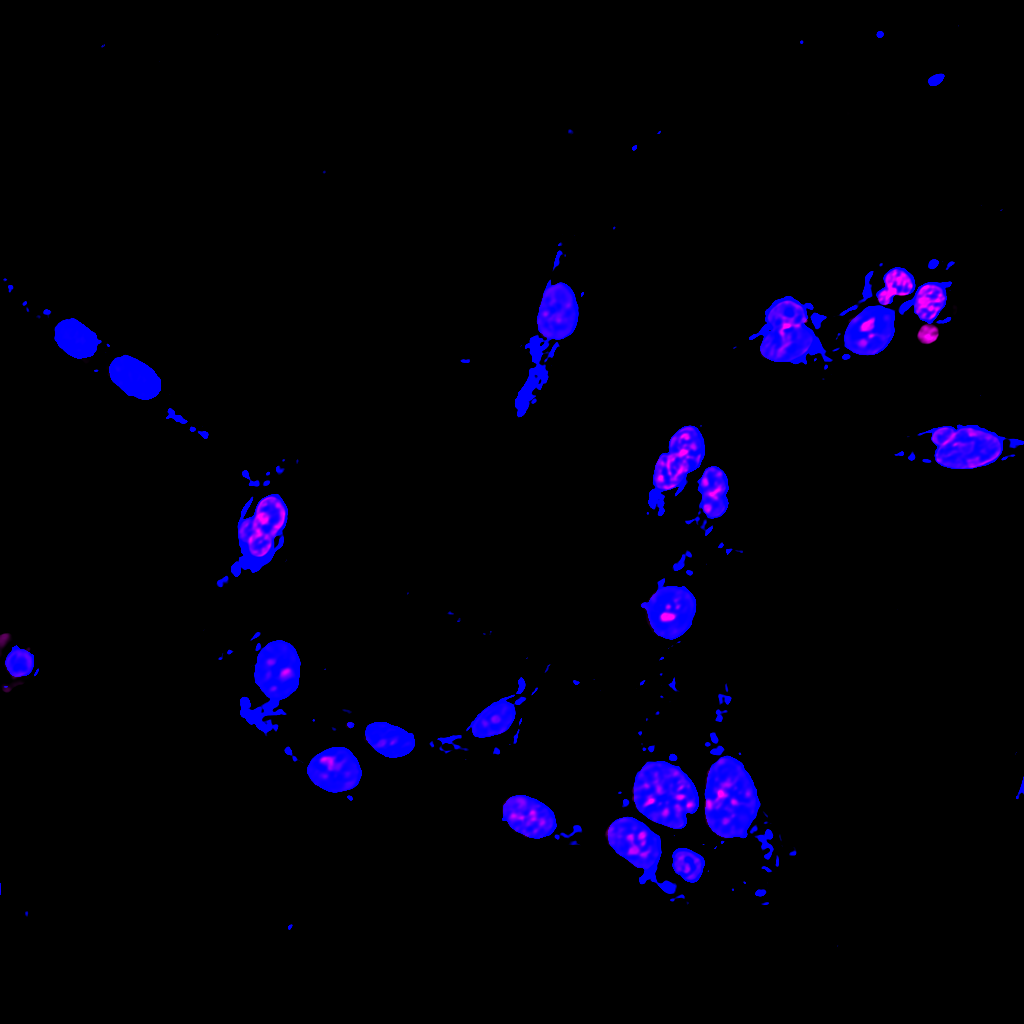

Supplement: Supplementary file 21 — Figure EV4 Source Data [file 44321_2025_260_MOESM21_ESM.zip › Figure EV4/EV4D/sh-ctrl vehicle 6X.png]

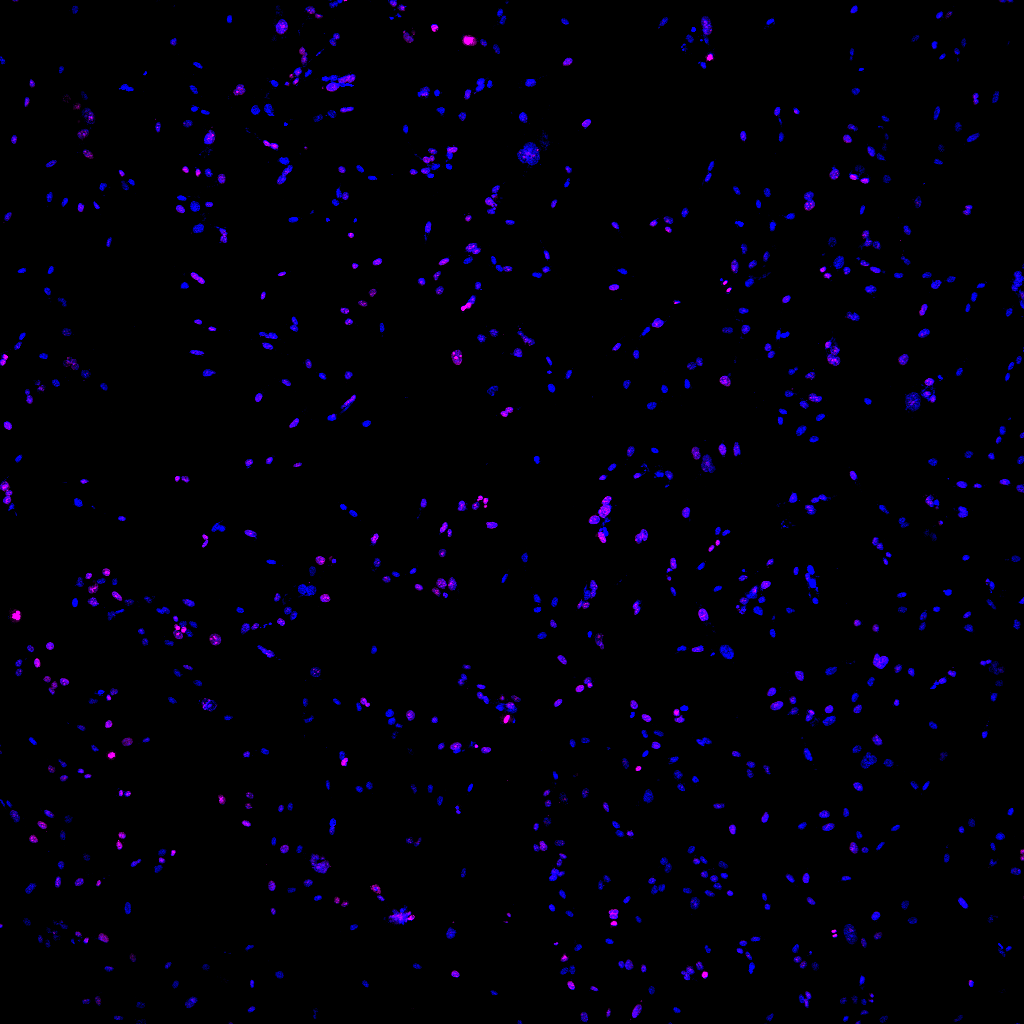

Supplement: Supplementary file 21 — Figure EV4 Source Data [file 44321_2025_260_MOESM21_ESM.zip › Figure EV4/EV4D/sh-ctrl vehicle.png]

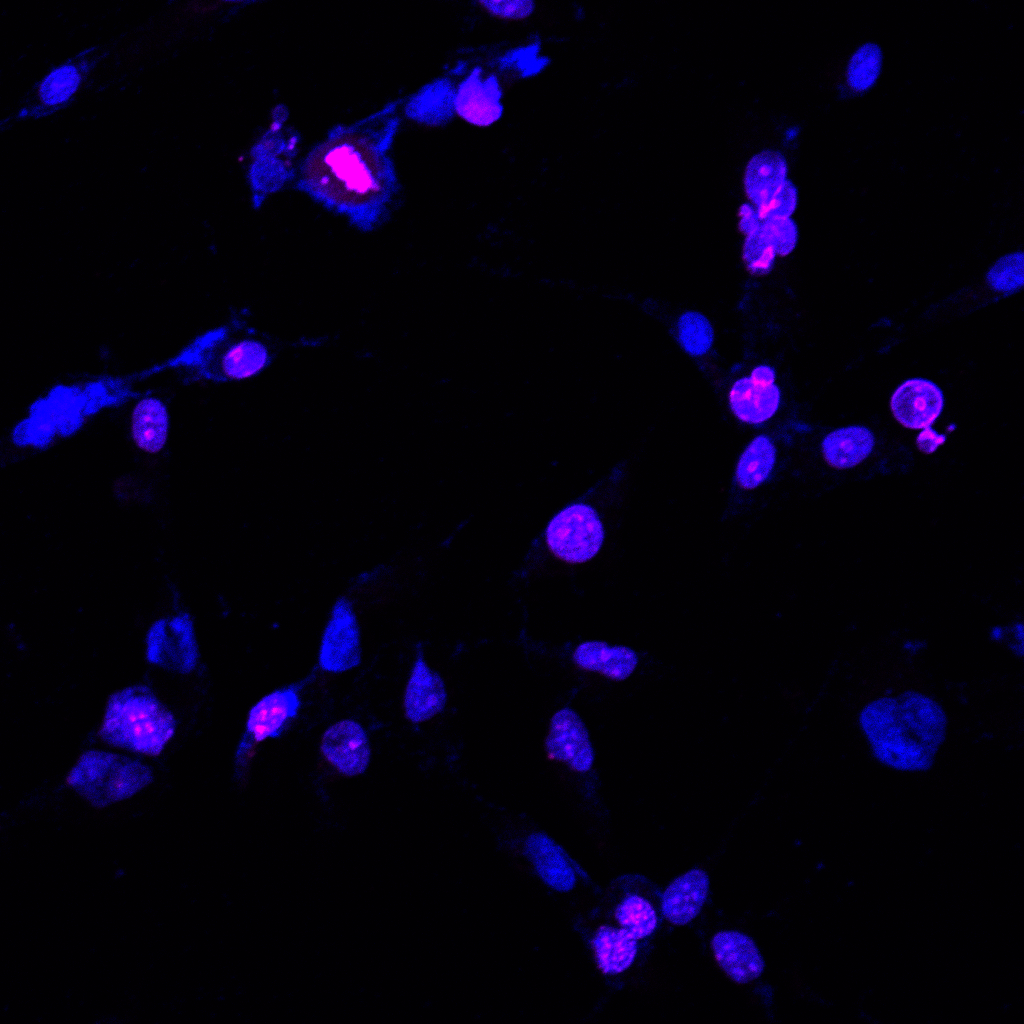

Supplement: Supplementary file 21 — Figure EV4 Source Data [file 44321_2025_260_MOESM21_ESM.zip › Figure EV4/EV4D/sh-ctrl+Reelin X6.png]

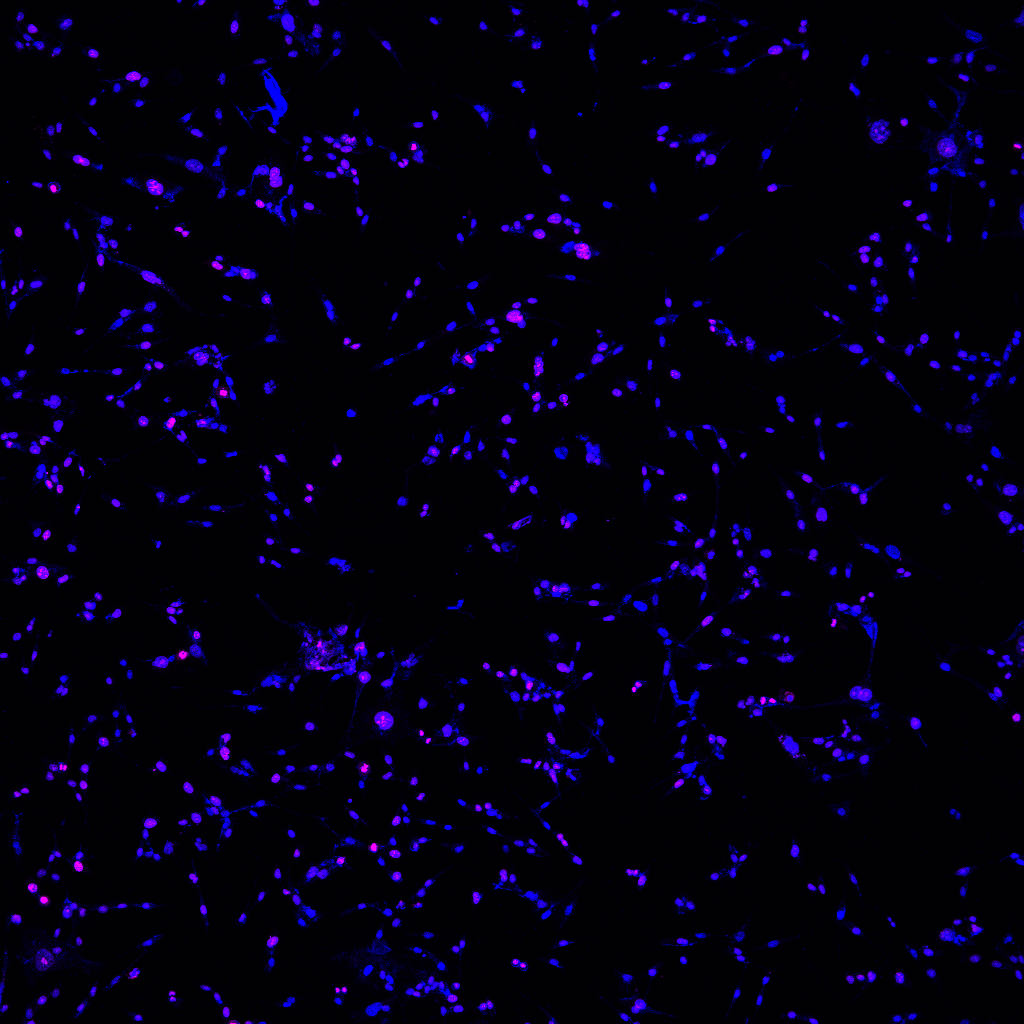

Supplement: Supplementary file 21 — Figure EV4 Source Data [file 44321_2025_260_MOESM21_ESM.zip › Figure EV4/EV4D/sh-ctrl+Reelin.png]

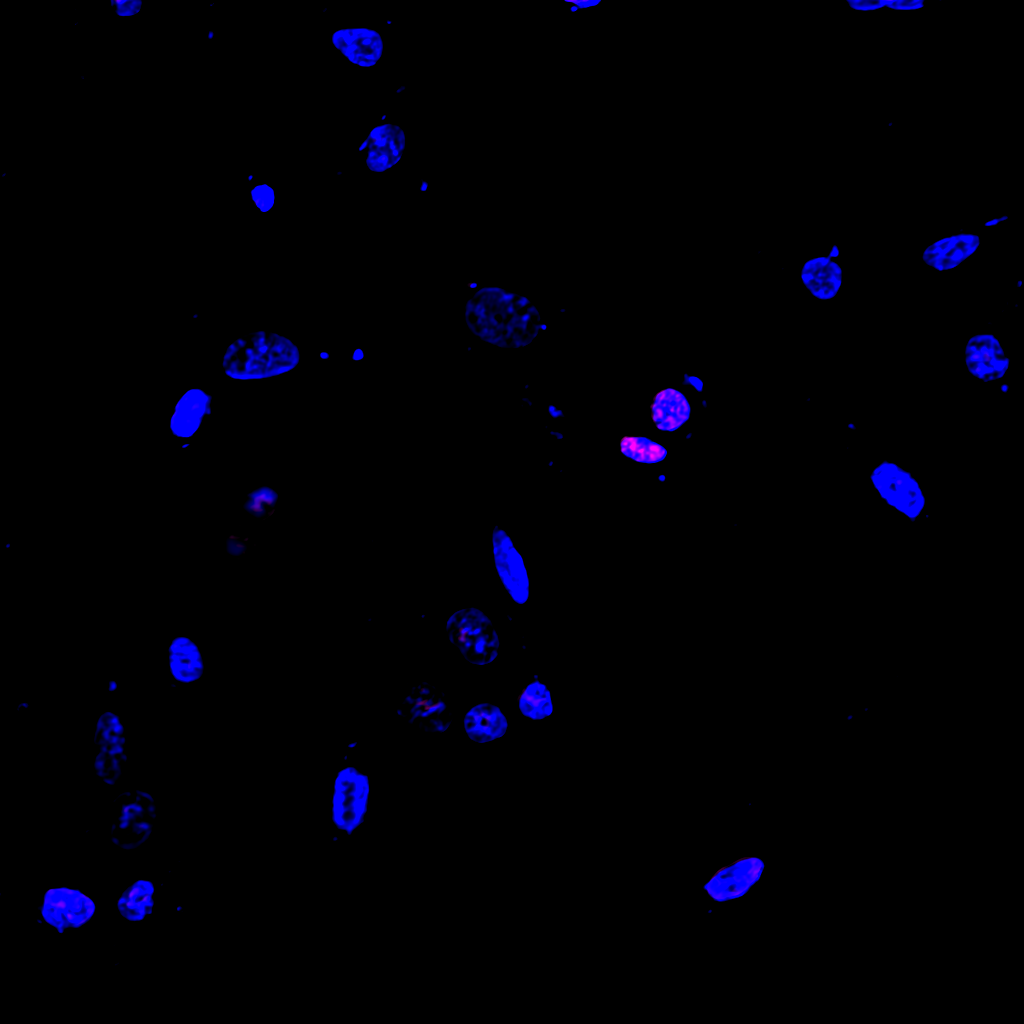

Supplement: Supplementary file 21 — Figure EV4 Source Data [file 44321_2025_260_MOESM21_ESM.zip › Figure EV4/EV4D/sh-LRP8-2# vehicle X6.png]

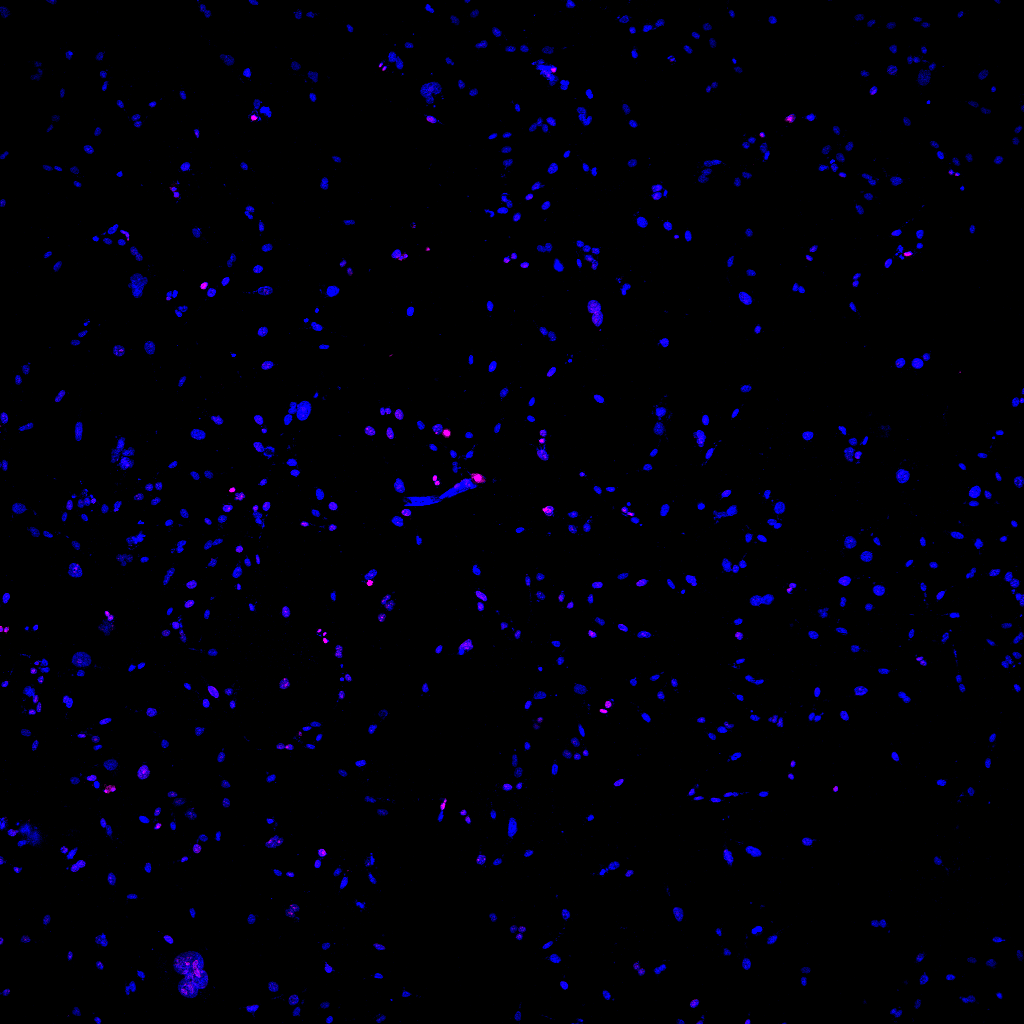

Supplement: Supplementary file 21 — Figure EV4 Source Data [file 44321_2025_260_MOESM21_ESM.zip › Figure EV4/EV4D/sh-LRP8-2# vehicle.png]

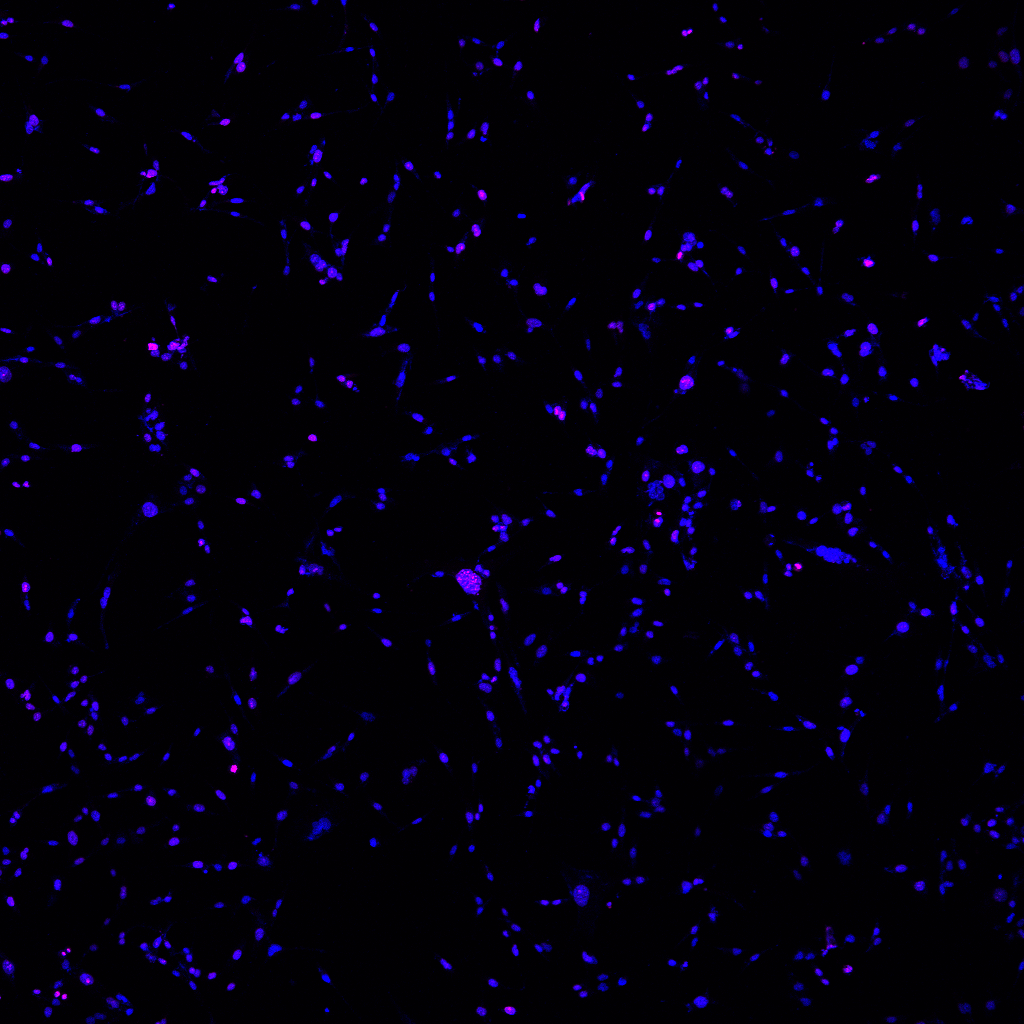

Supplement: Supplementary file 21 — Figure EV4 Source Data [file 44321_2025_260_MOESM21_ESM.zip › Figure EV4/EV4D/sh-LRP8-2#+Reelin.png]

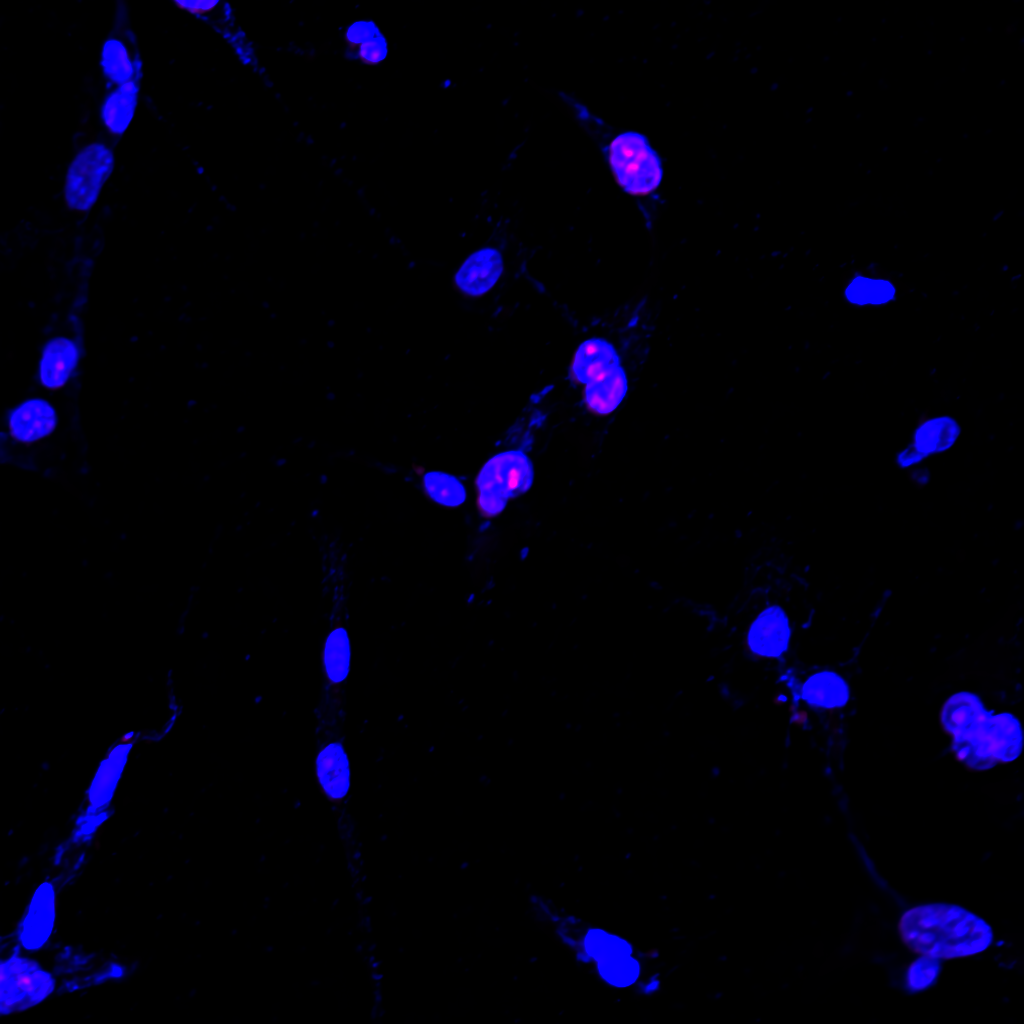

Supplement: Supplementary file 21 — Figure EV4 Source Data [file 44321_2025_260_MOESM21_ESM.zip › Figure EV4/EV4D/sh-LRP8-5#+Reelin X6.png]

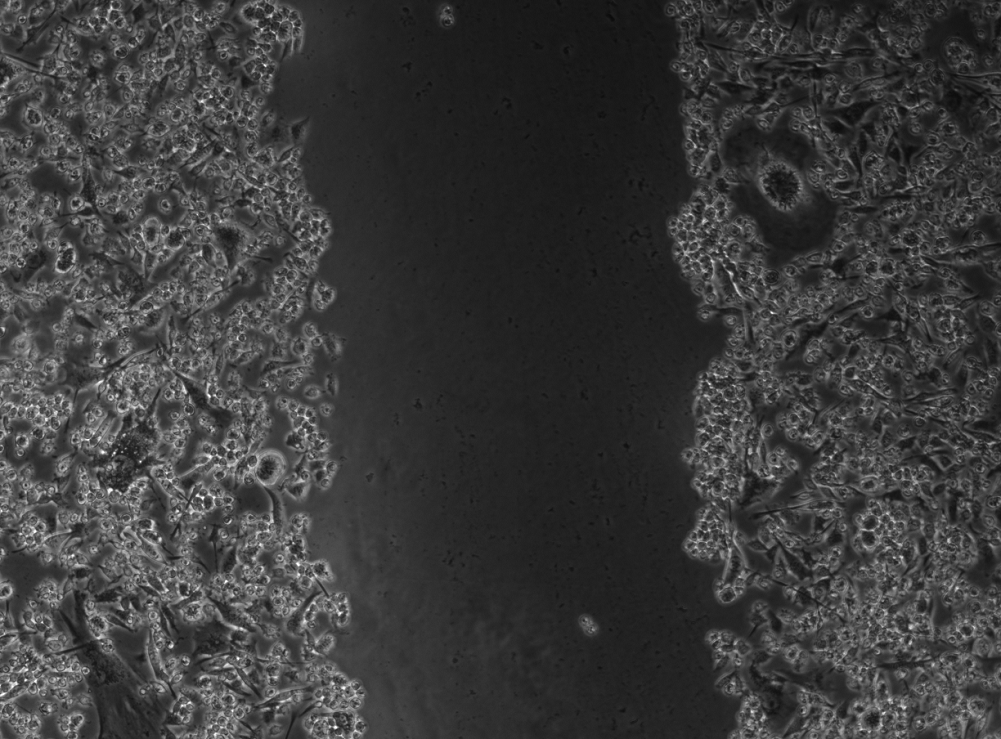

Supplement: Supplementary file 21 — Figure EV4 Source Data [file 44321_2025_260_MOESM21_ESM.zip › Figure EV4/EV4G/sh-ctrl vehicle 0h.png]

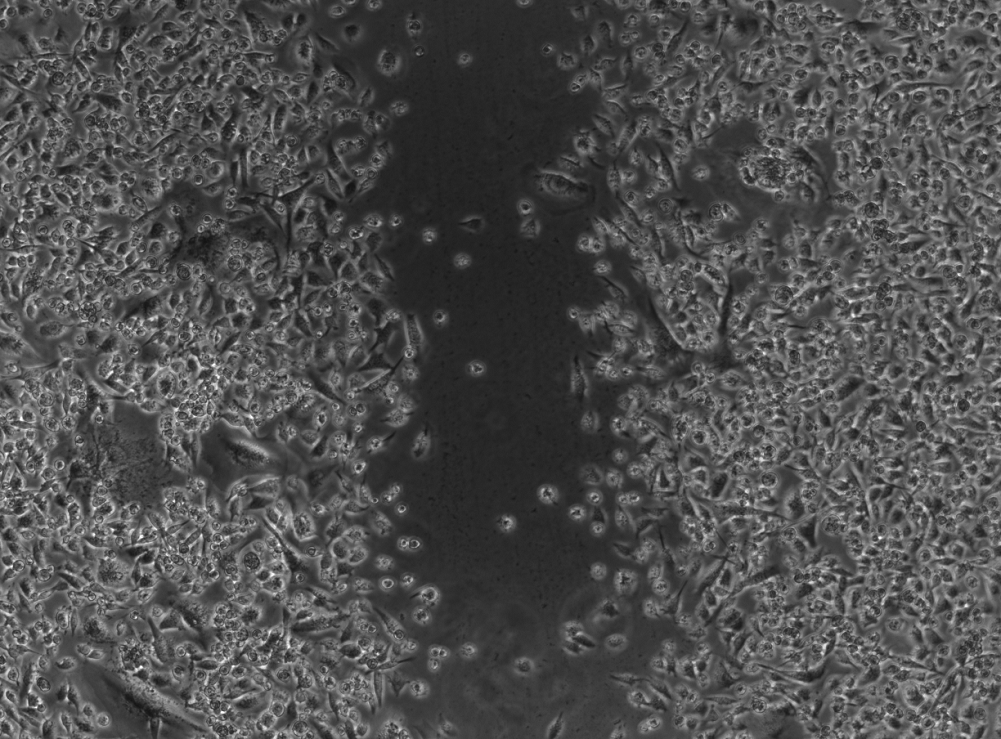

Supplement: Supplementary file 21 — Figure EV4 Source Data [file 44321_2025_260_MOESM21_ESM.zip › Figure EV4/EV4G/sh-ctrl vehicle 24h.png]

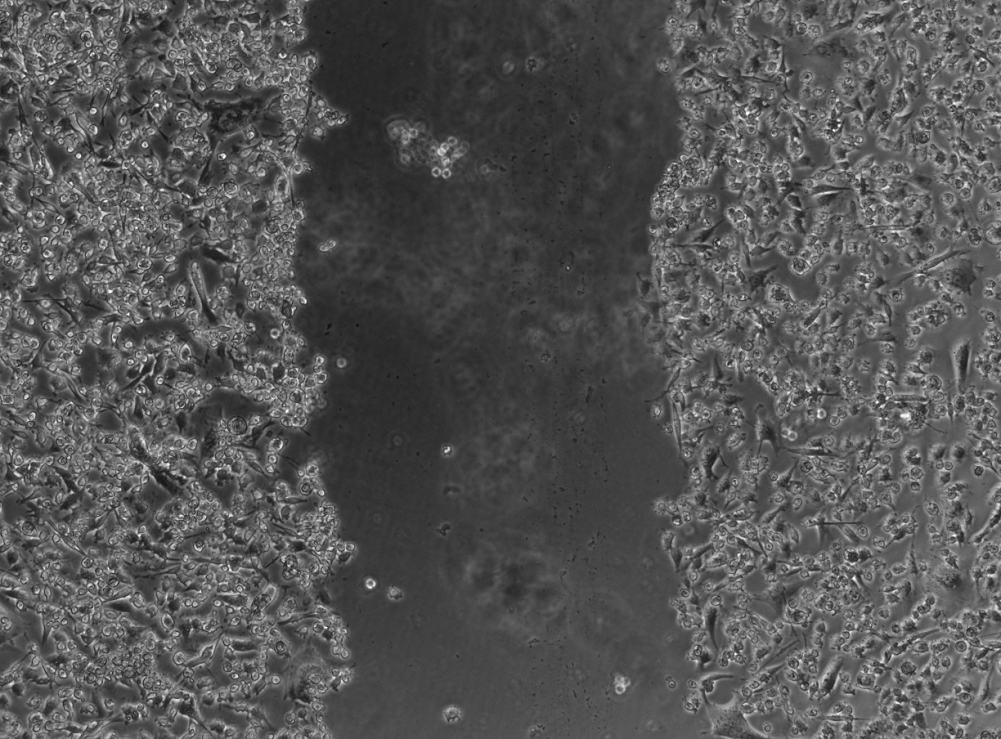

Supplement: Supplementary file 21 — Figure EV4 Source Data [file 44321_2025_260_MOESM21_ESM.zip › Figure EV4/EV4G/sh-ctrl+Reelin 0h.png]

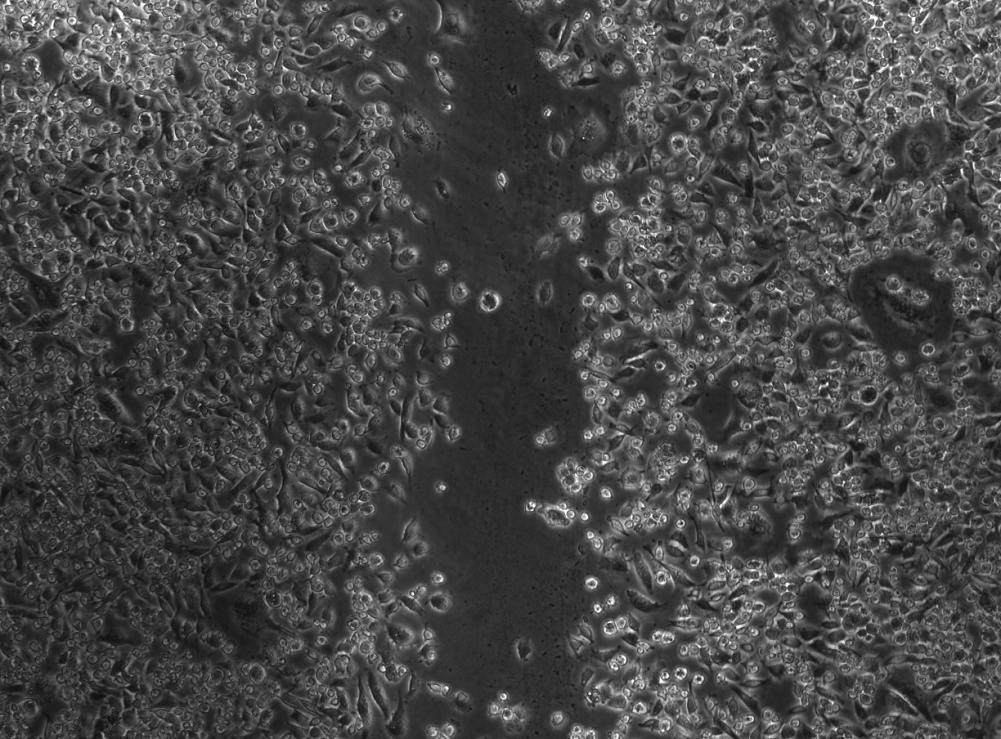

Supplement: Supplementary file 21 — Figure EV4 Source Data [file 44321_2025_260_MOESM21_ESM.zip › Figure EV4/EV4G/sh-ctrl+Reelin 24h.png]

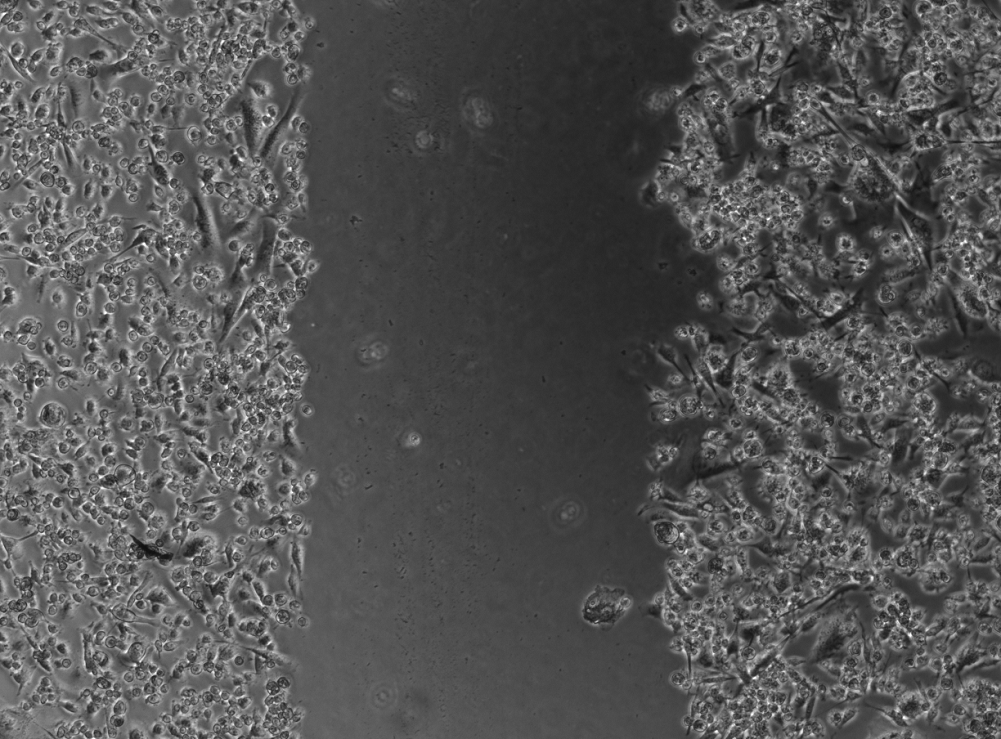

Supplement: Supplementary file 21 — Figure EV4 Source Data [file 44321_2025_260_MOESM21_ESM.zip › Figure EV4/EV4G/sh-LRP8-2# 0h.png]

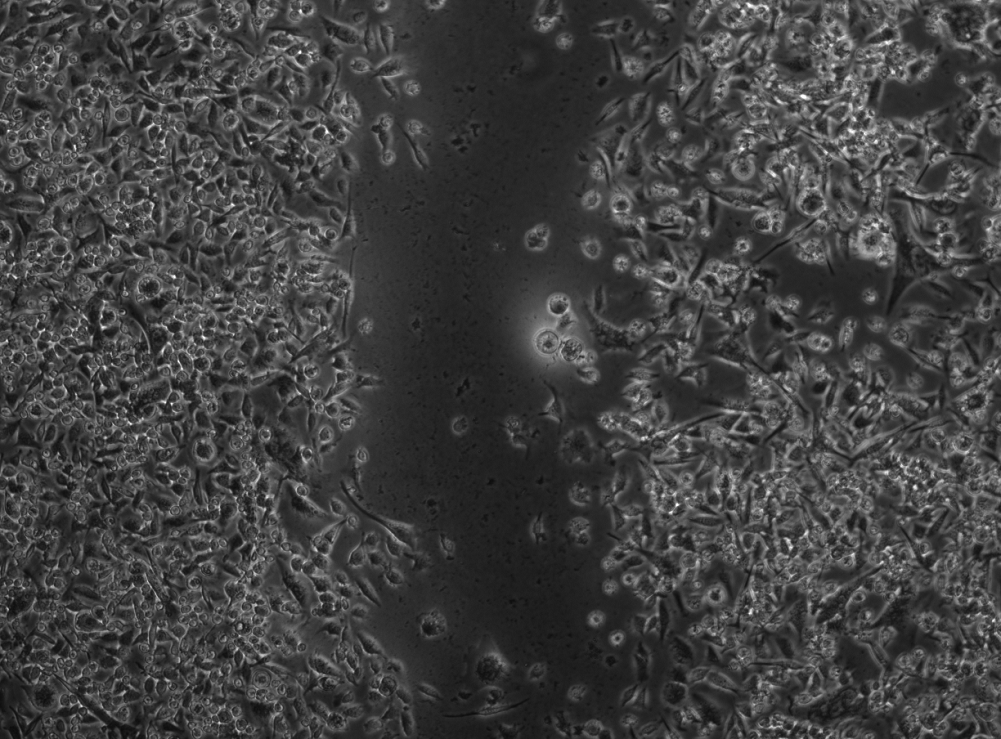

Supplement: Supplementary file 21 — Figure EV4 Source Data [file 44321_2025_260_MOESM21_ESM.zip › Figure EV4/EV4G/sh-LRP8-2# 24h.png]

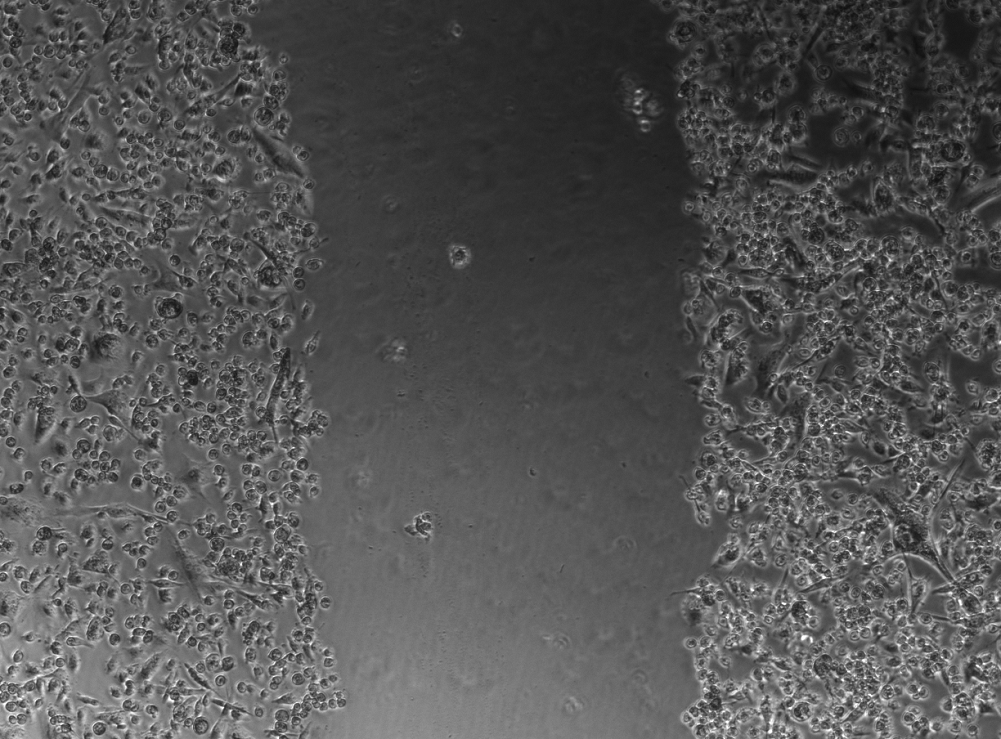

Supplement: Supplementary file 21 — Figure EV4 Source Data [file 44321_2025_260_MOESM21_ESM.zip › Figure EV4/EV4G/sh-LRP8-2#+Reelin 0h.png]

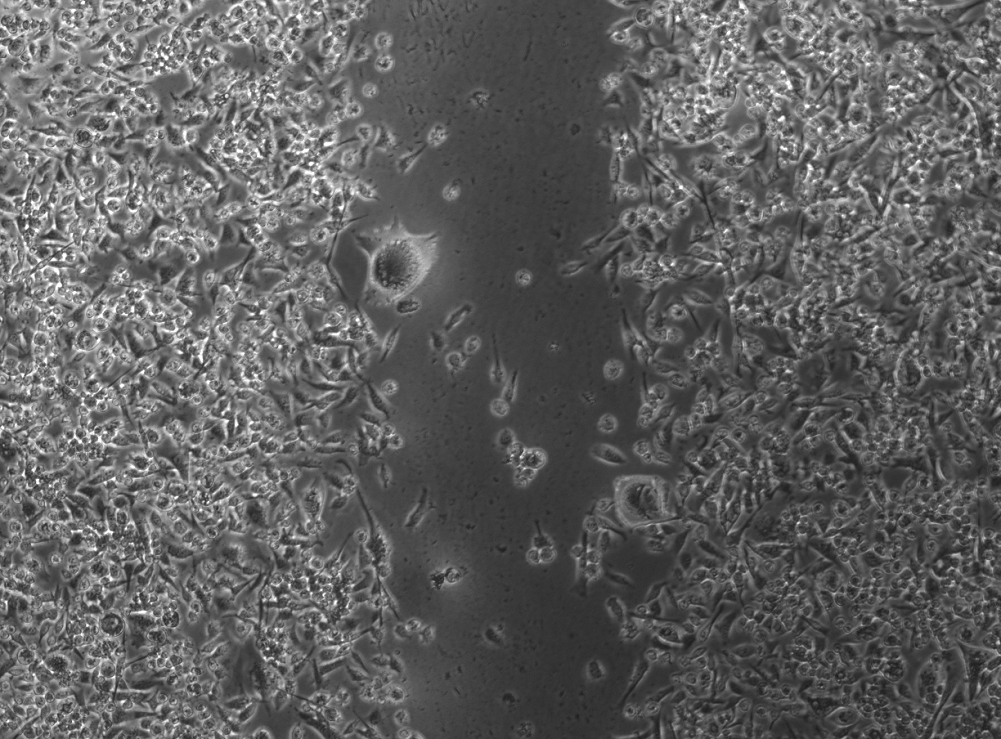

Supplement: Supplementary file 21 — Figure EV4 Source Data [file 44321_2025_260_MOESM21_ESM.zip › Figure EV4/EV4G/sh-LRP8-2#+Reelin 24h.png]

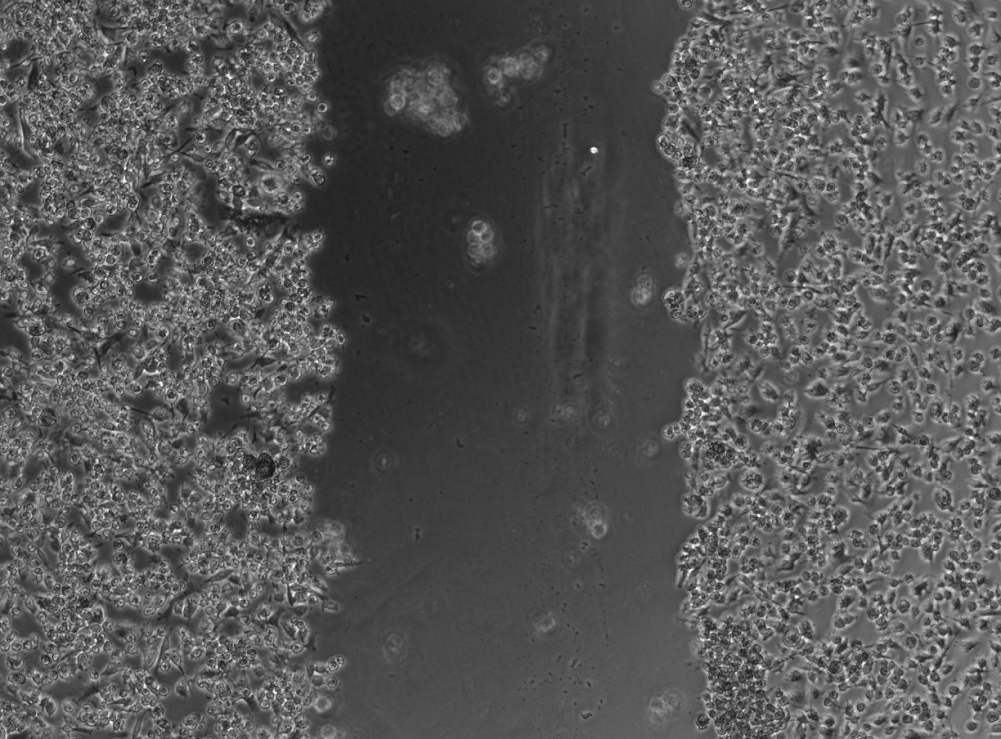

Supplement: Supplementary file 21 — Figure EV4 Source Data [file 44321_2025_260_MOESM21_ESM.zip › Figure EV4/EV4I/BM0 0h.png]

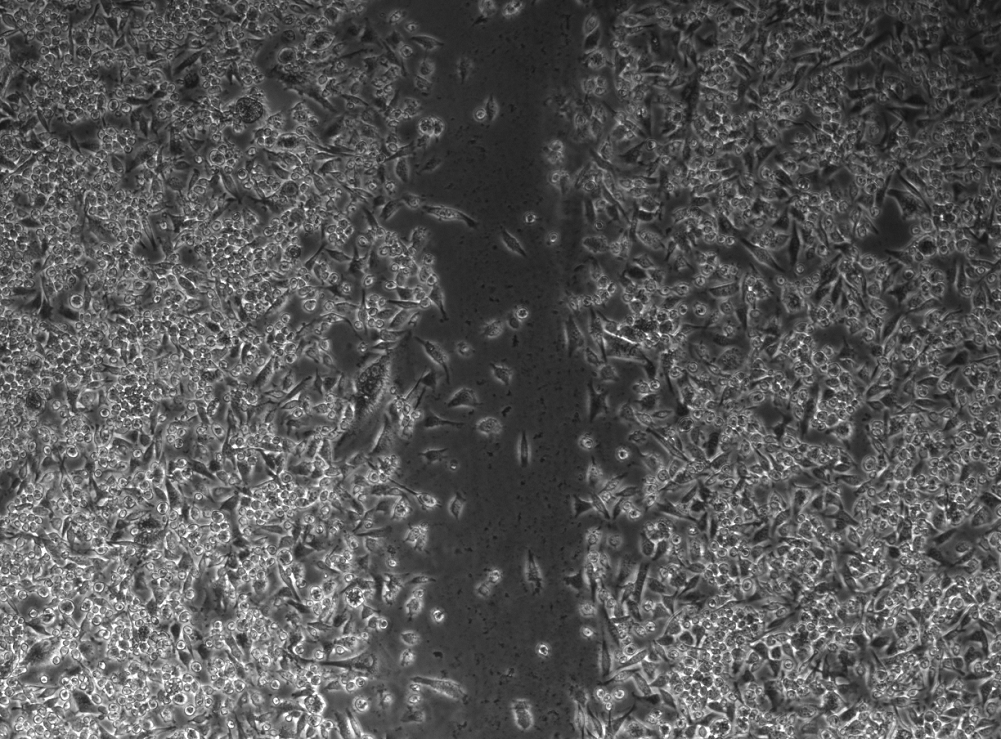

Supplement: Supplementary file 21 — Figure EV4 Source Data [file 44321_2025_260_MOESM21_ESM.zip › Figure EV4/EV4I/BM0 24h.png]

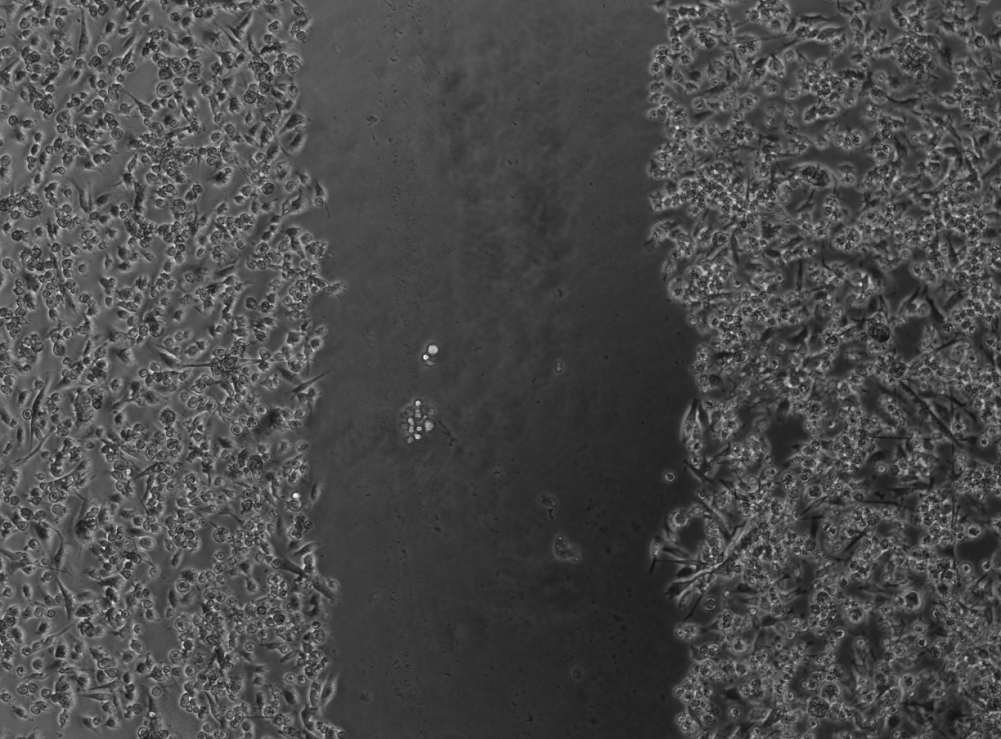

Supplement: Supplementary file 21 — Figure EV4 Source Data [file 44321_2025_260_MOESM21_ESM.zip › Figure EV4/EV4I/BM6 0h.png]

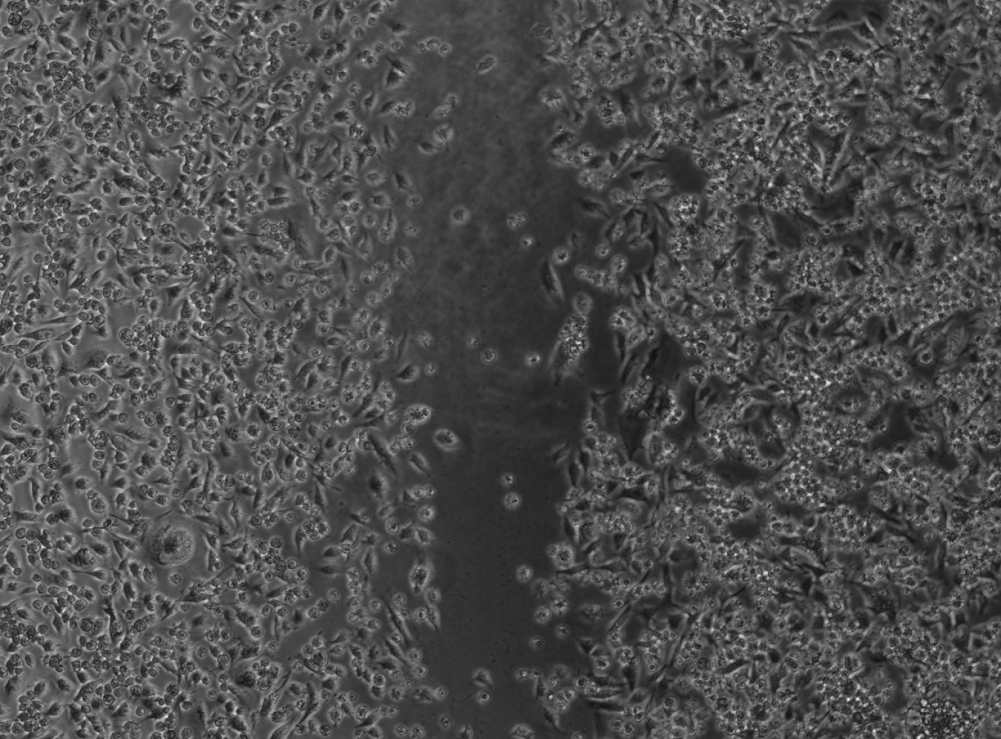

Supplement: Supplementary file 21 — Figure EV4 Source Data [file 44321_2025_260_MOESM21_ESM.zip › Figure EV4/EV4I/BM6 24h.png]

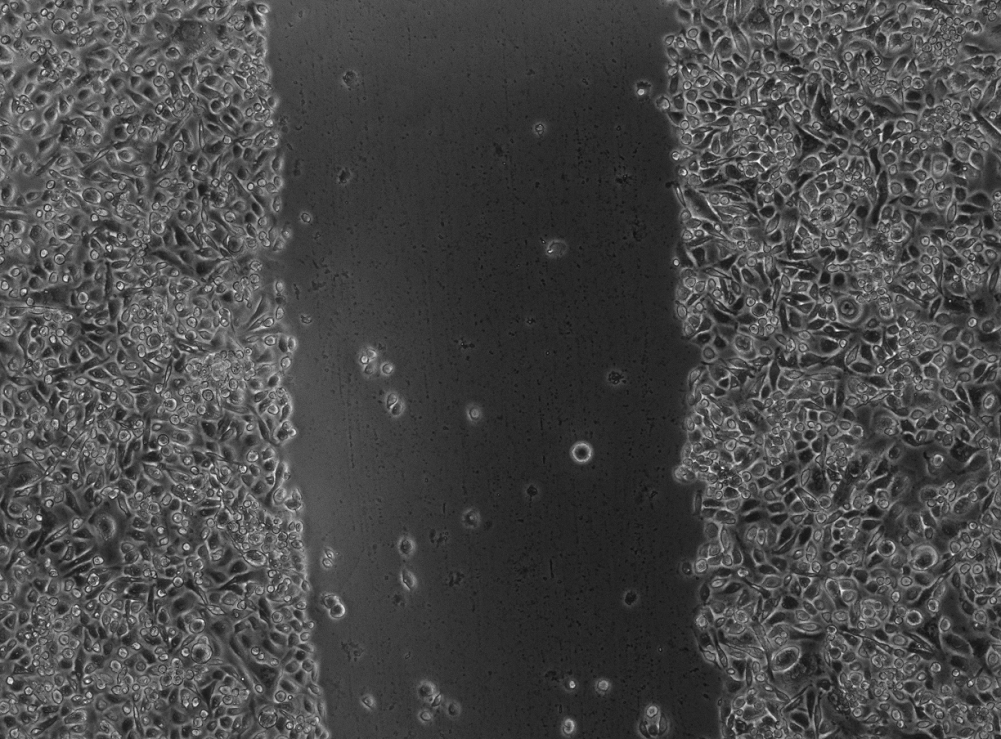

Supplement: Supplementary file 22 — Figure EV5 Source Data [file 44321_2025_260_MOESM22_ESM.zip › Figure EV5/EV5C/MEN10207 0h.png]

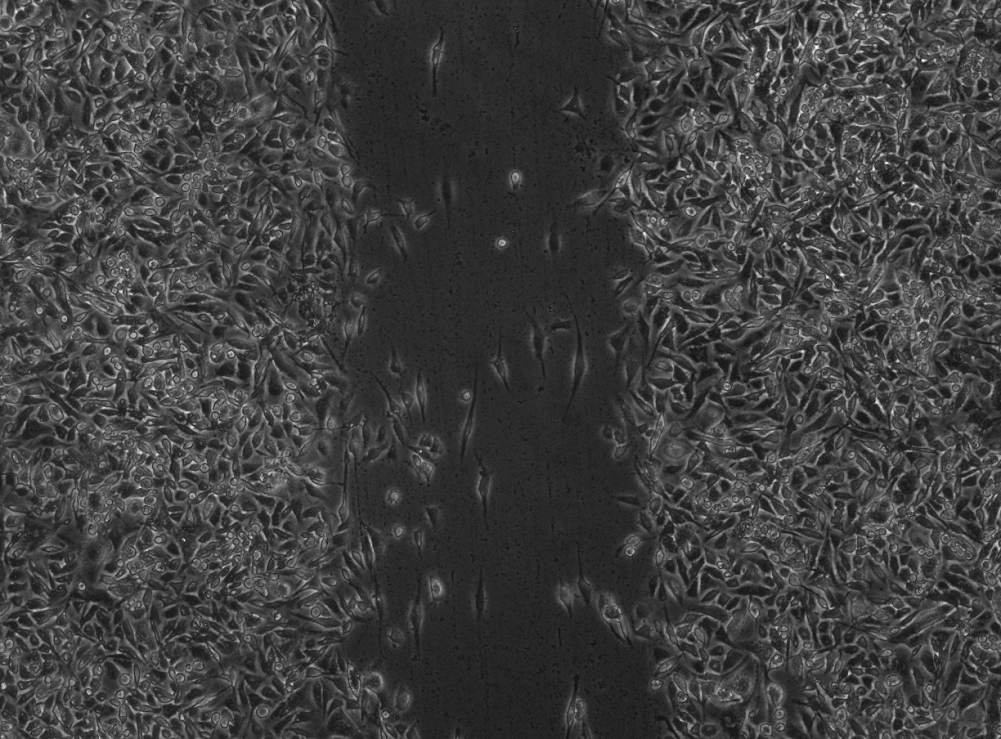

Supplement: Supplementary file 22 — Figure EV5 Source Data [file 44321_2025_260_MOESM22_ESM.zip › Figure EV5/EV5C/MEN10207 24h.png]

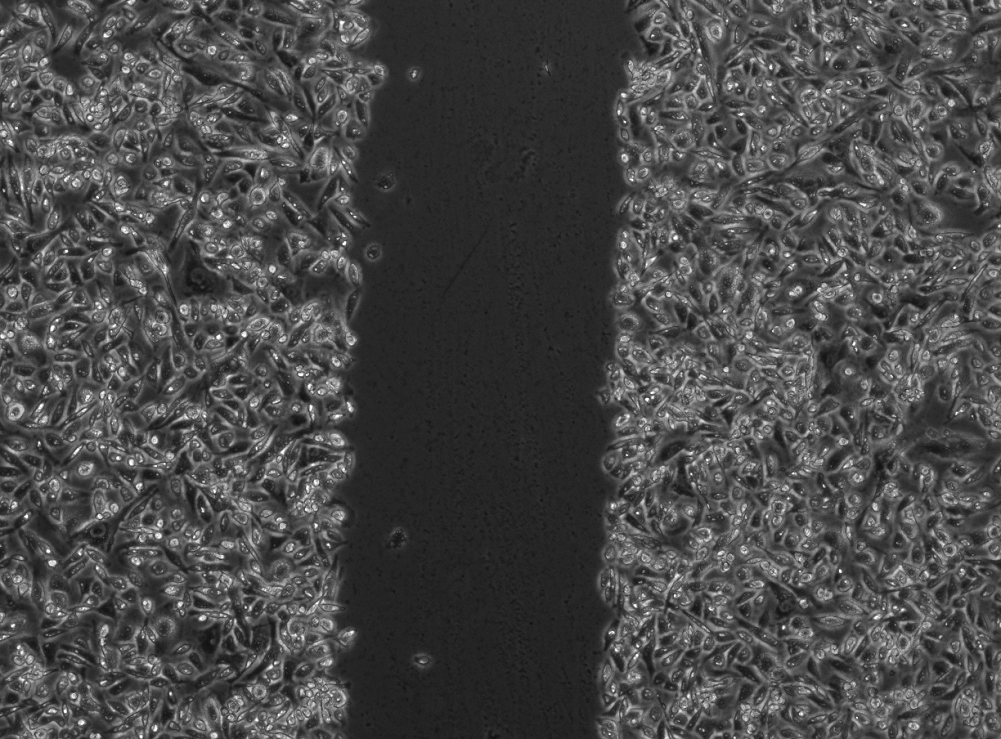

Supplement: Supplementary file 22 — Figure EV5 Source Data [file 44321_2025_260_MOESM22_ESM.zip › Figure EV5/EV5C/OB 0h.png]

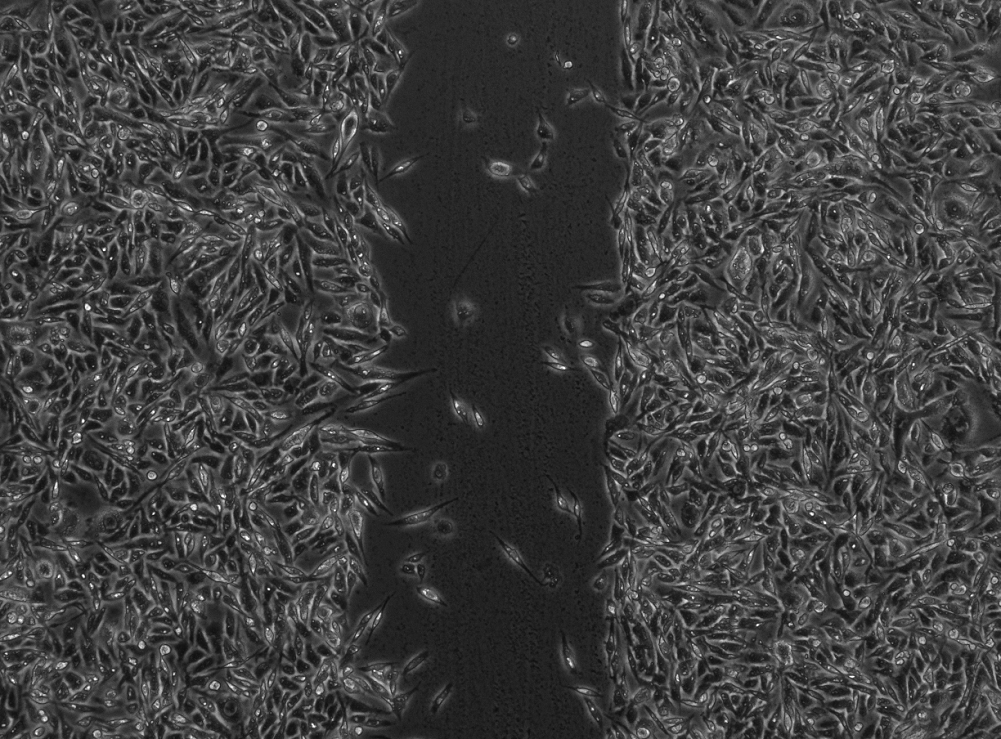

Supplement: Supplementary file 22 — Figure EV5 Source Data [file 44321_2025_260_MOESM22_ESM.zip › Figure EV5/EV5C/OB 24h.png]

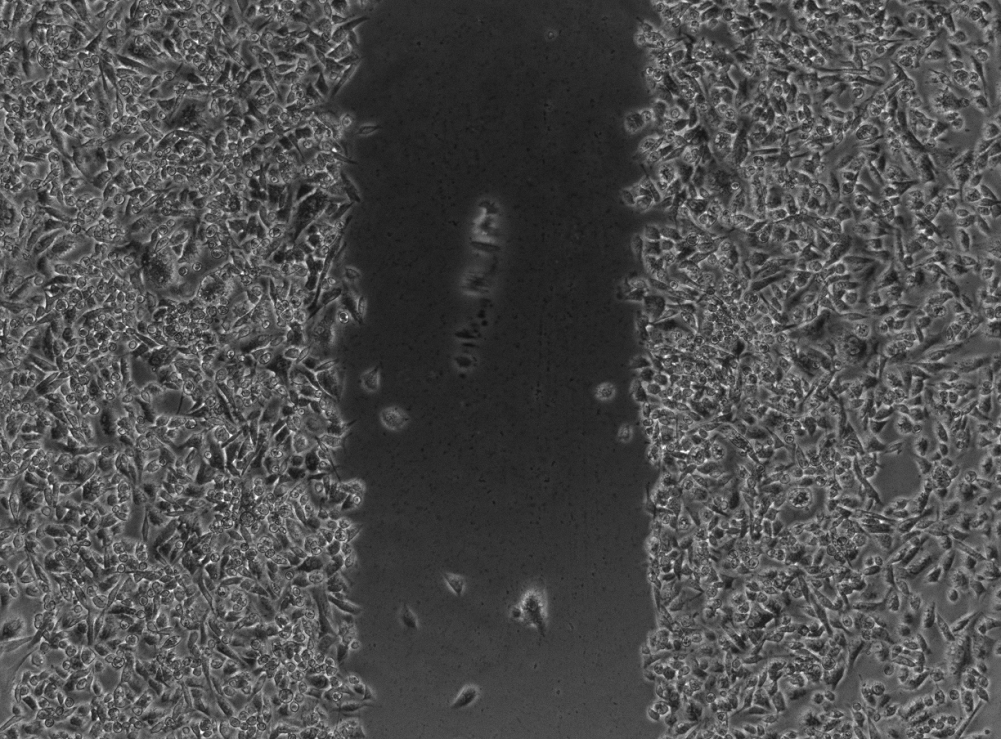

Supplement: Supplementary file 22 — Figure EV5 Source Data [file 44321_2025_260_MOESM22_ESM.zip › Figure EV5/EV5C/vehicle 0h.png]

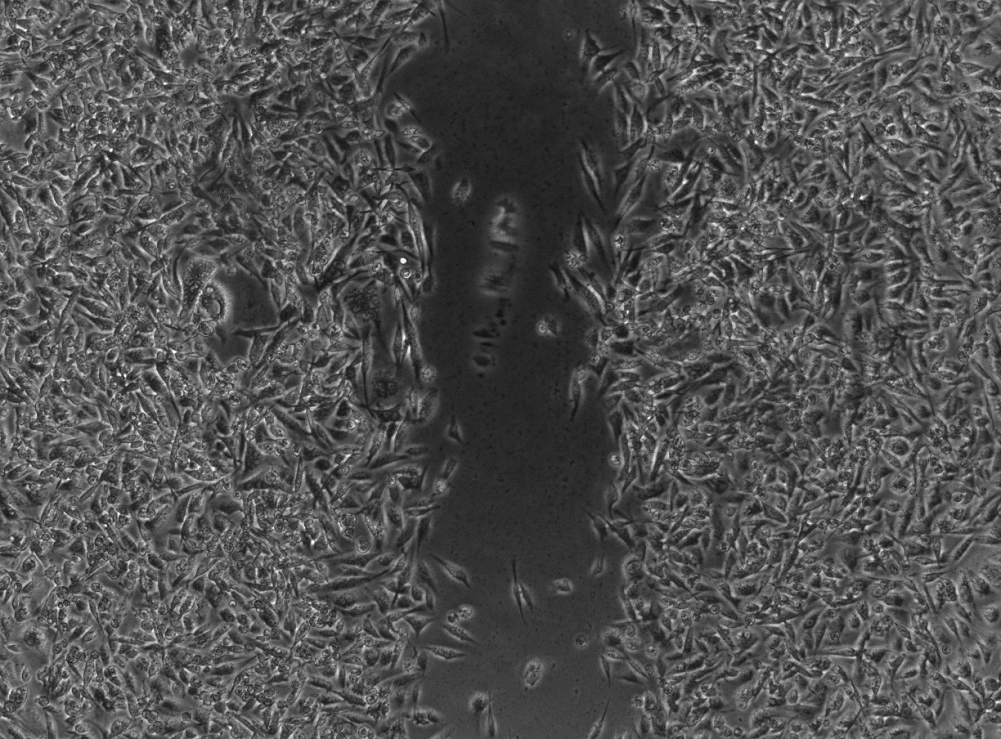

Supplement: Supplementary file 22 — Figure EV5 Source Data [file 44321_2025_260_MOESM22_ESM.zip › Figure EV5/EV5C/vehicle 24h.png]

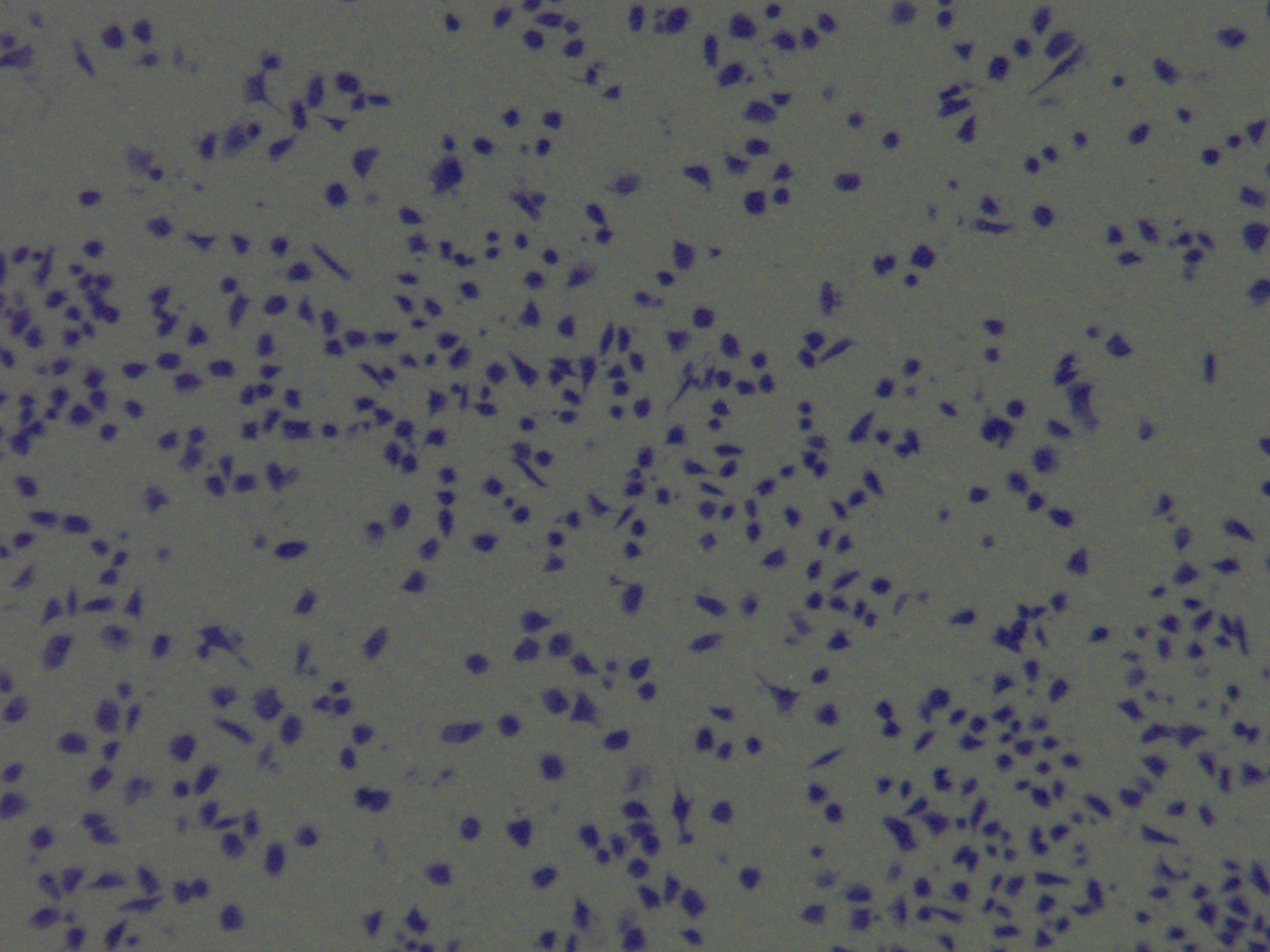

Supplement: Supplementary file 22 — Figure EV5 Source Data [file 44321_2025_260_MOESM22_ESM.zip › Figure EV5/EV5D/MEN10207 invasion.tif]

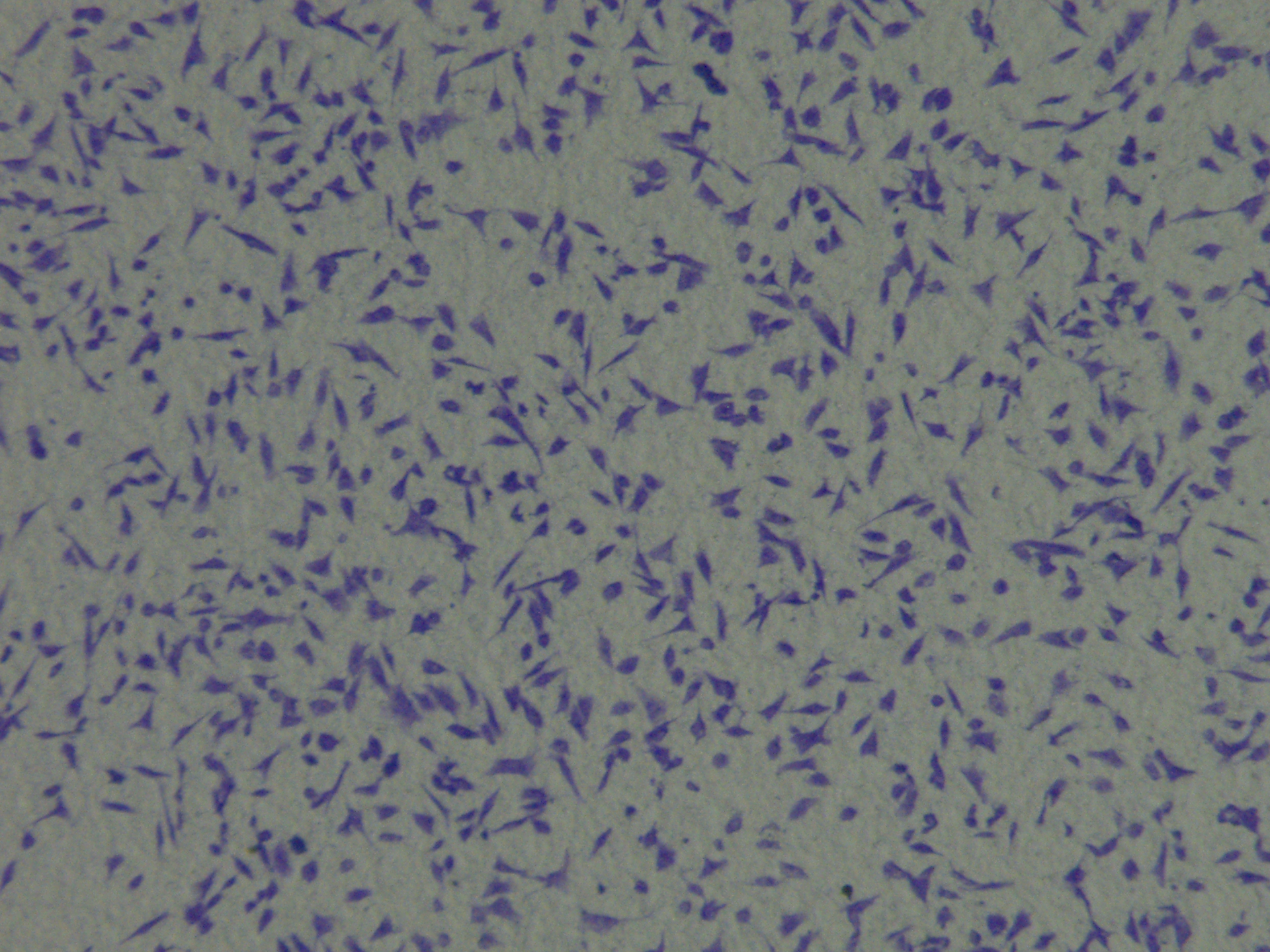

Supplement: Supplementary file 22 — Figure EV5 Source Data [file 44321_2025_260_MOESM22_ESM.zip › Figure EV5/EV5D/MEN10207 migration.tif]

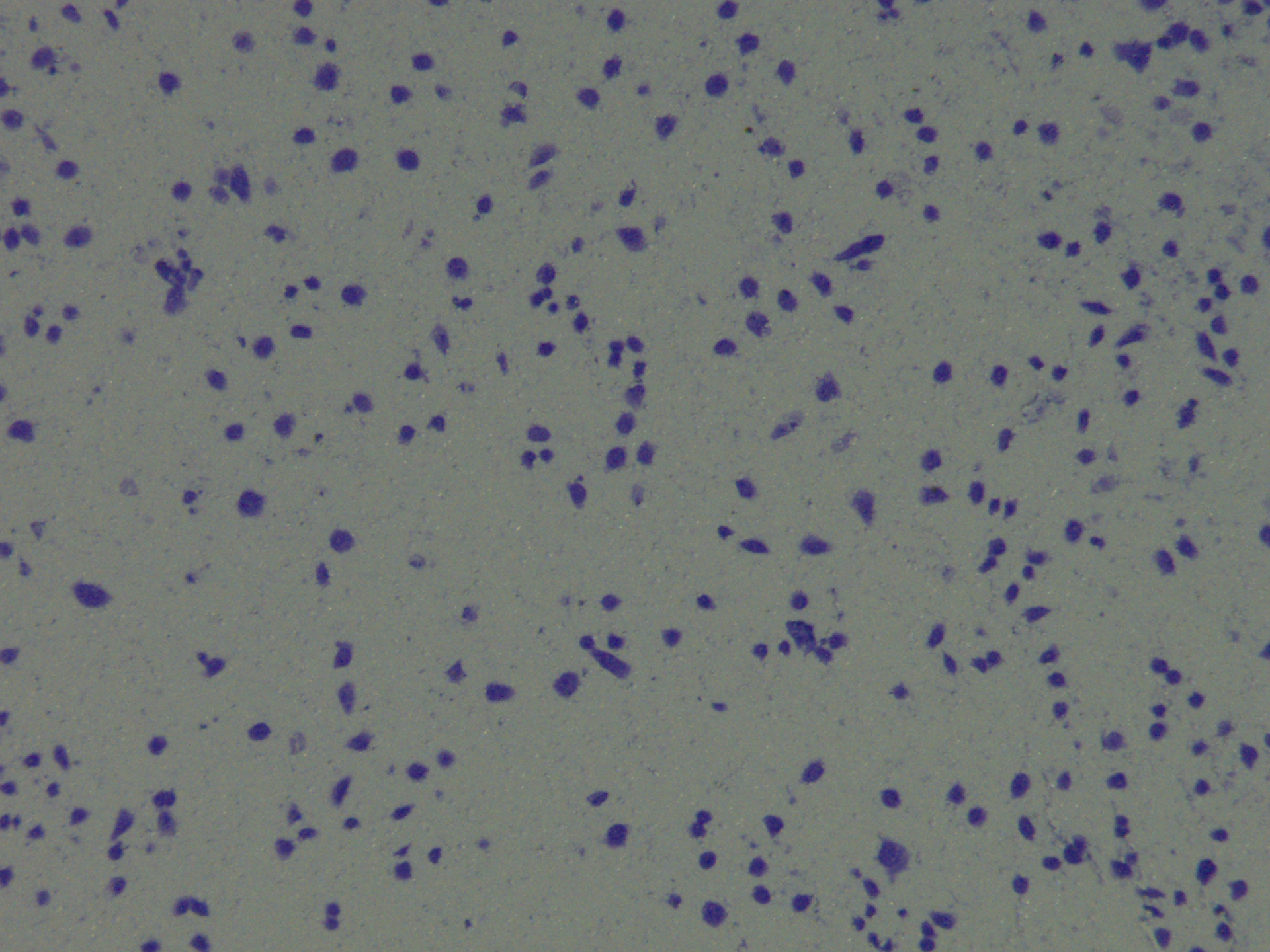

Supplement: Supplementary file 22 — Figure EV5 Source Data [file 44321_2025_260_MOESM22_ESM.zip › Figure EV5/EV5D/OB invasion.tif]

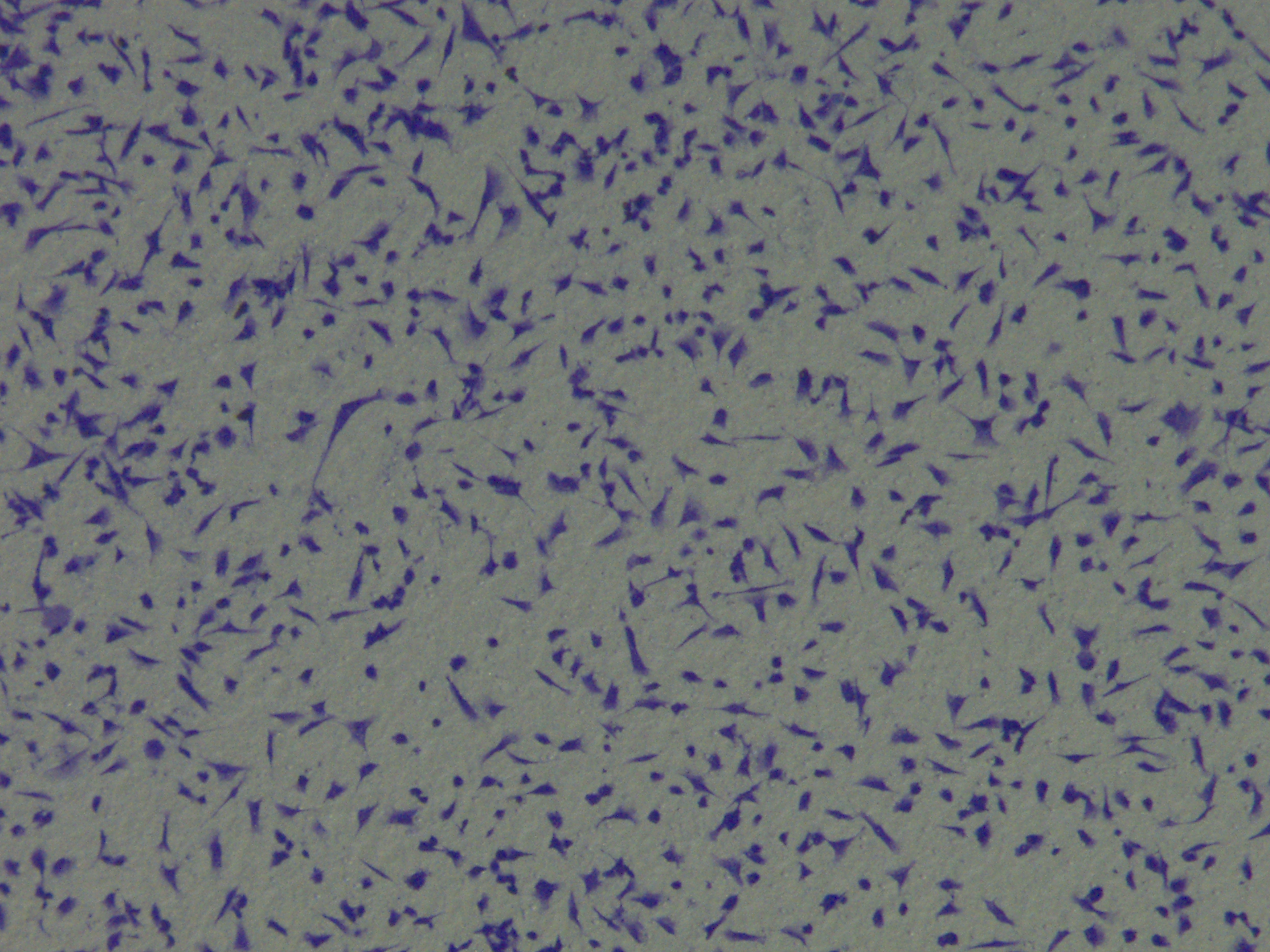

Supplement: Supplementary file 22 — Figure EV5 Source Data [file 44321_2025_260_MOESM22_ESM.zip › Figure EV5/EV5D/OB migration.tif]

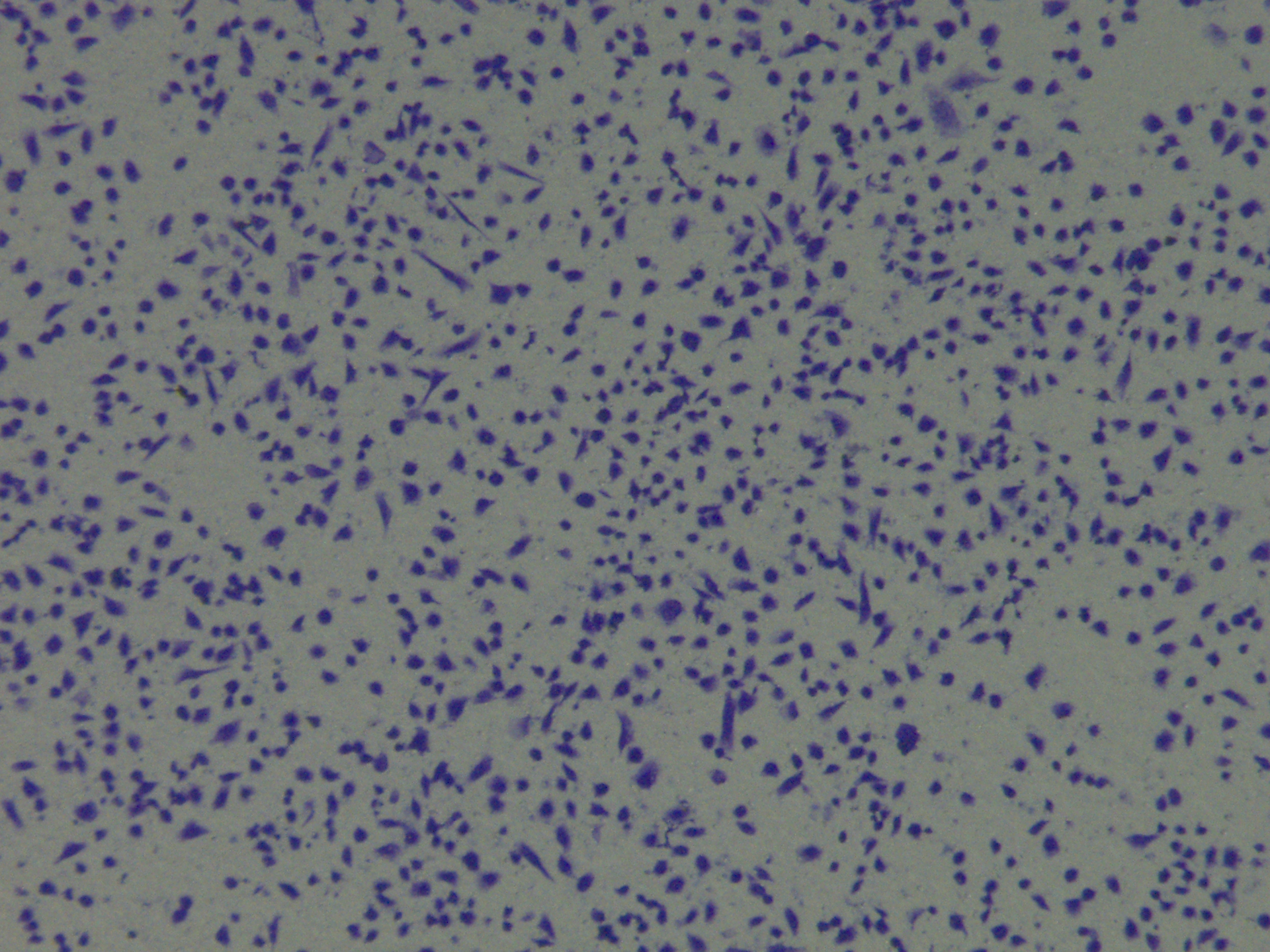

Supplement: Supplementary file 22 — Figure EV5 Source Data [file 44321_2025_260_MOESM22_ESM.zip › Figure EV5/EV5D/vehicle invasion.tif]

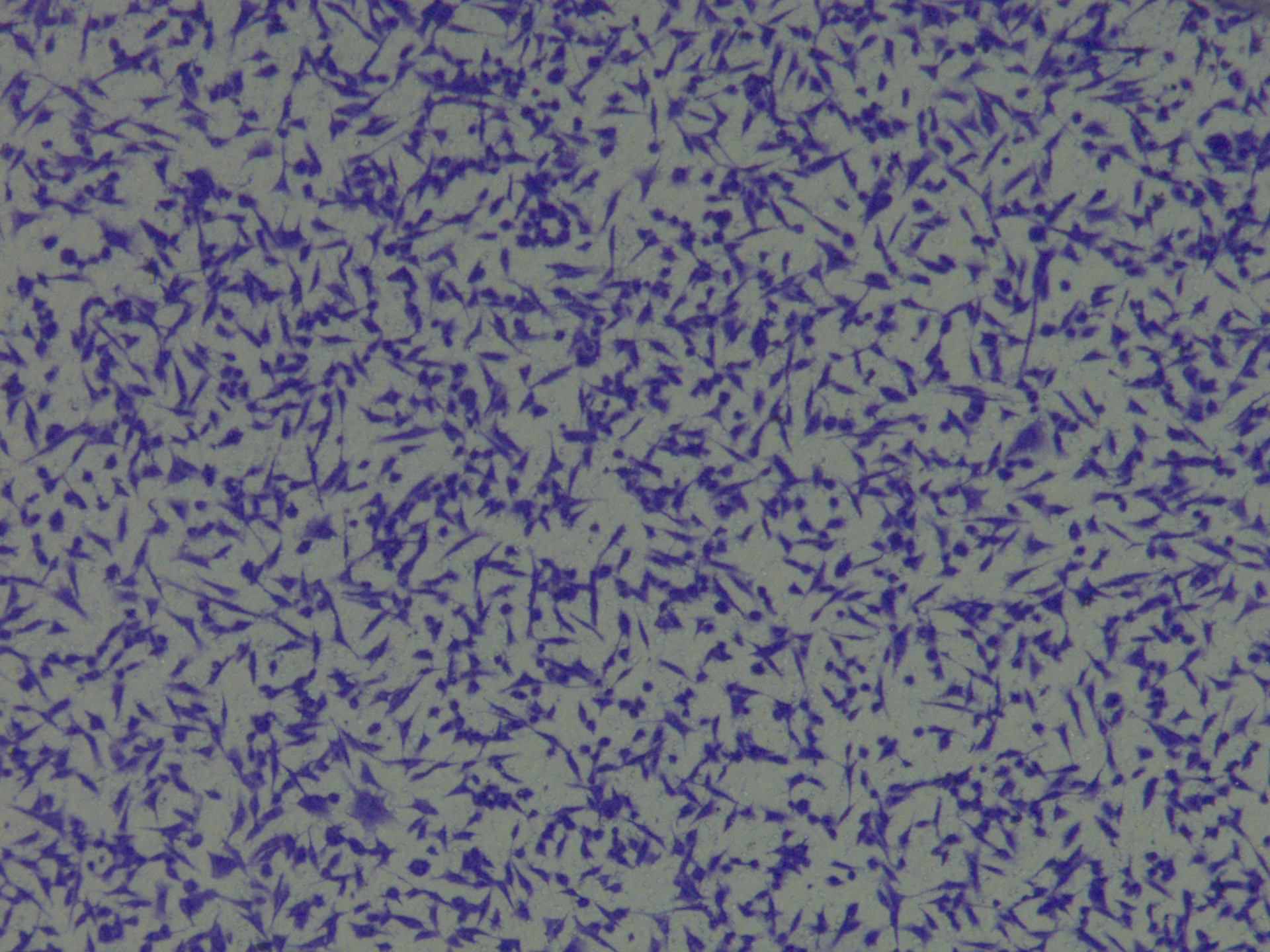

Supplement: Supplementary file 22 — Figure EV5 Source Data [file 44321_2025_260_MOESM22_ESM.zip › Figure EV5/EV5D/vehicle migration.tif]

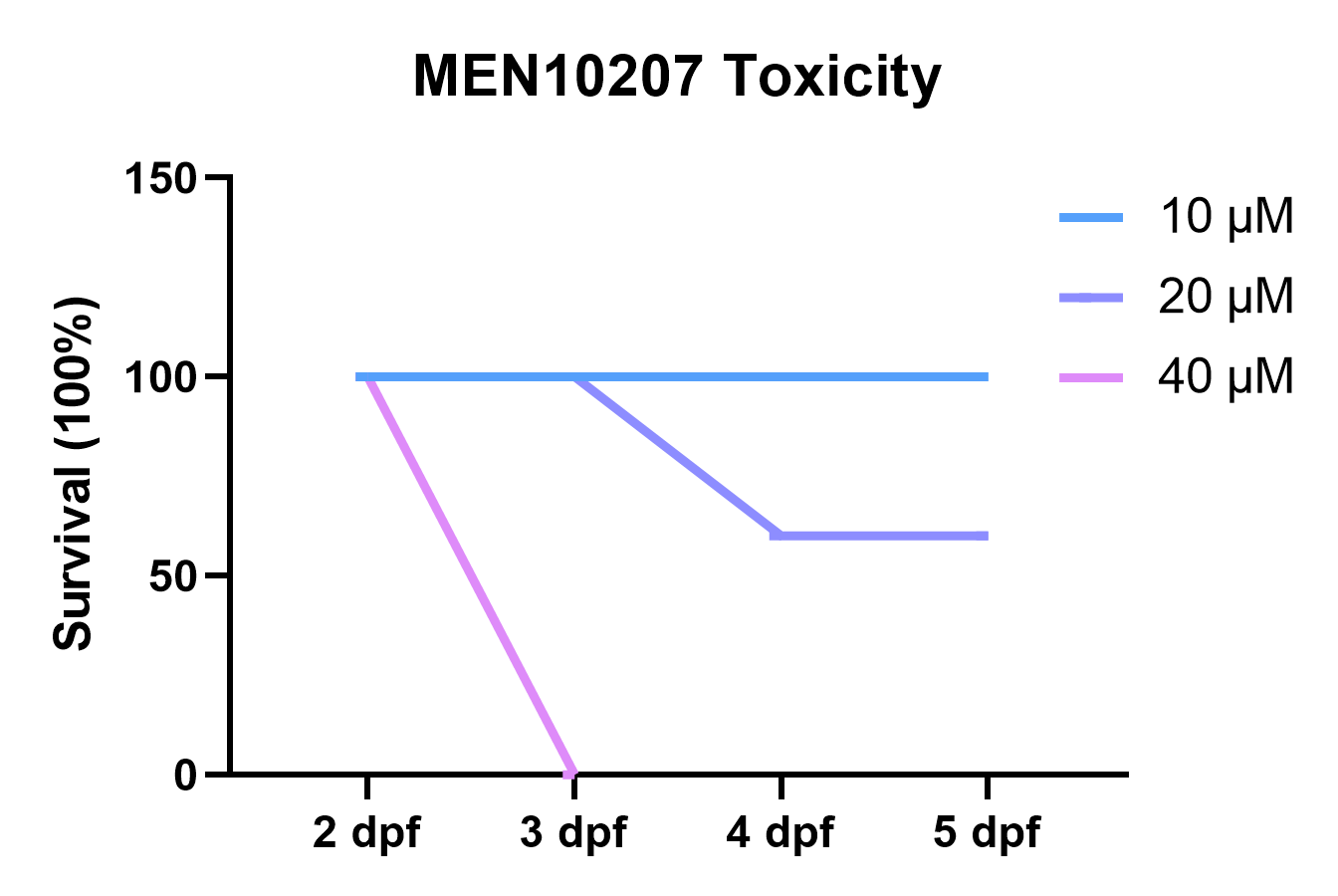

Supplement: Supplementary file 22 — Figure EV5 Source Data [file 44321_2025_260_MOESM22_ESM.zip › Figure EV5/EV5H/MEN10207 Toxicity.tif]

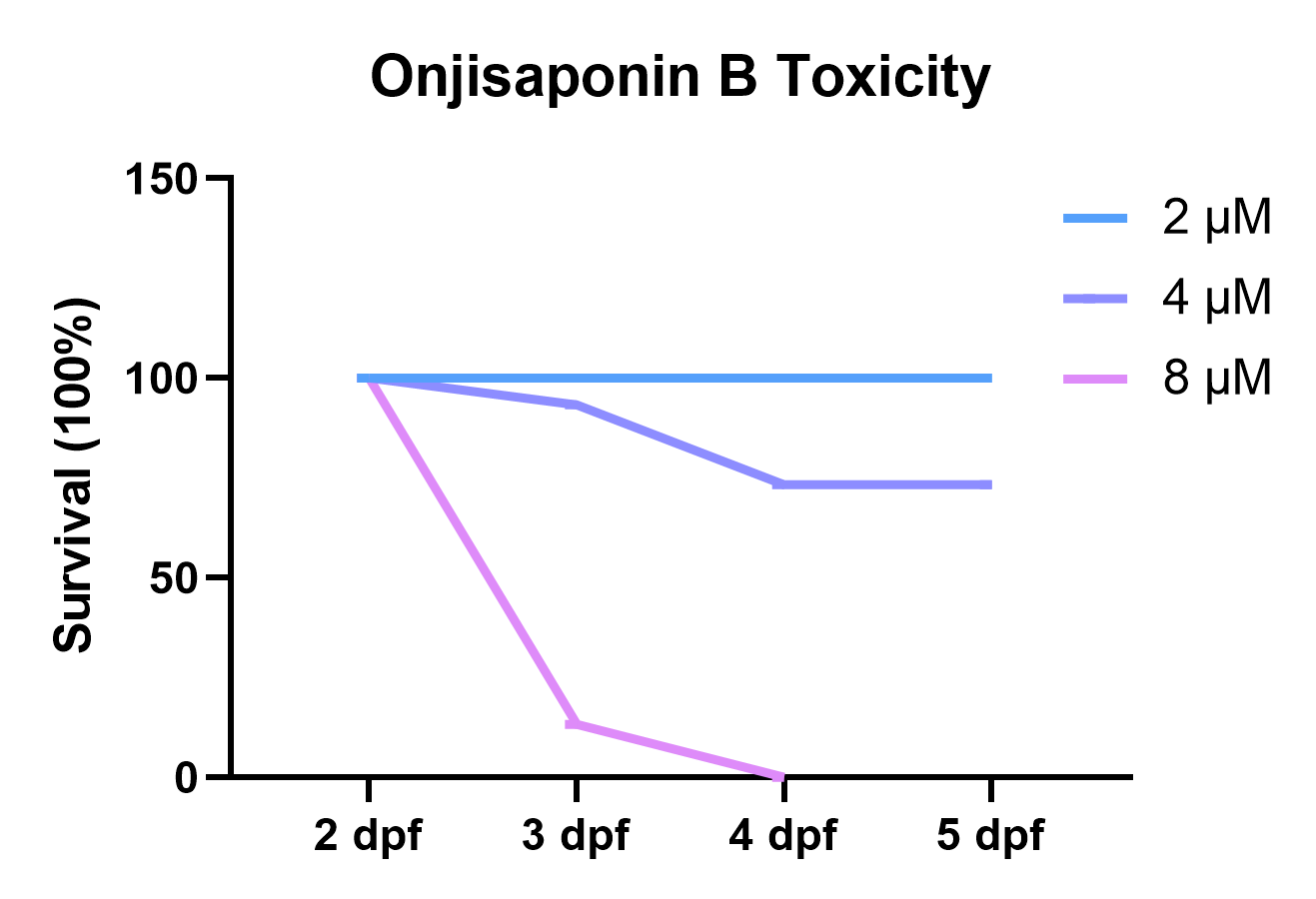

Supplement: Supplementary file 22 — Figure EV5 Source Data [file 44321_2025_260_MOESM22_ESM.zip › Figure EV5/EV5H/Onjisaponin B Toxicity.tif]

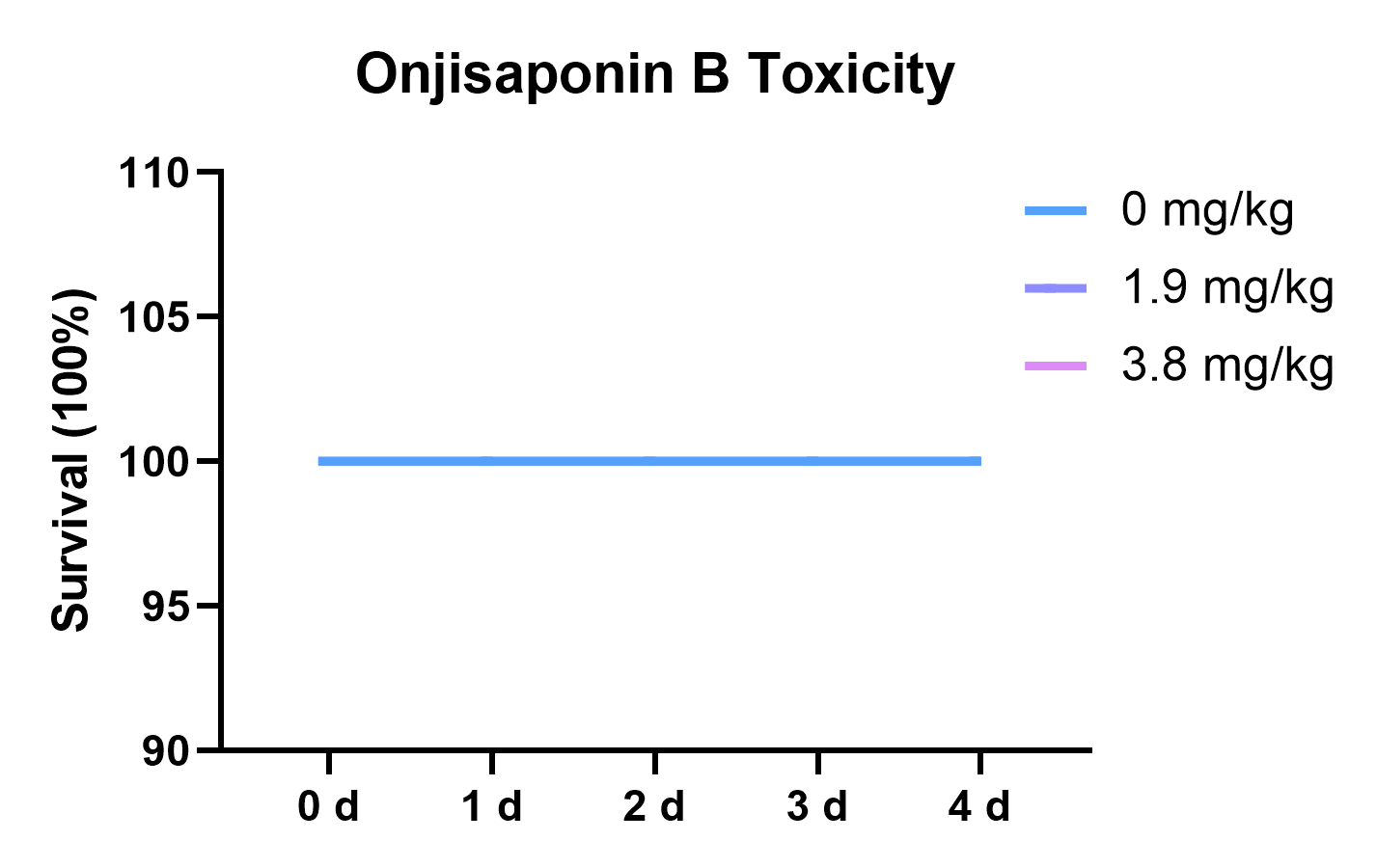

Supplement: Supplementary file 22 — Figure EV5 Source Data [file 44321_2025_260_MOESM22_ESM.zip › Figure EV5/EV5I/MEN 10207 Toxicity.tif]

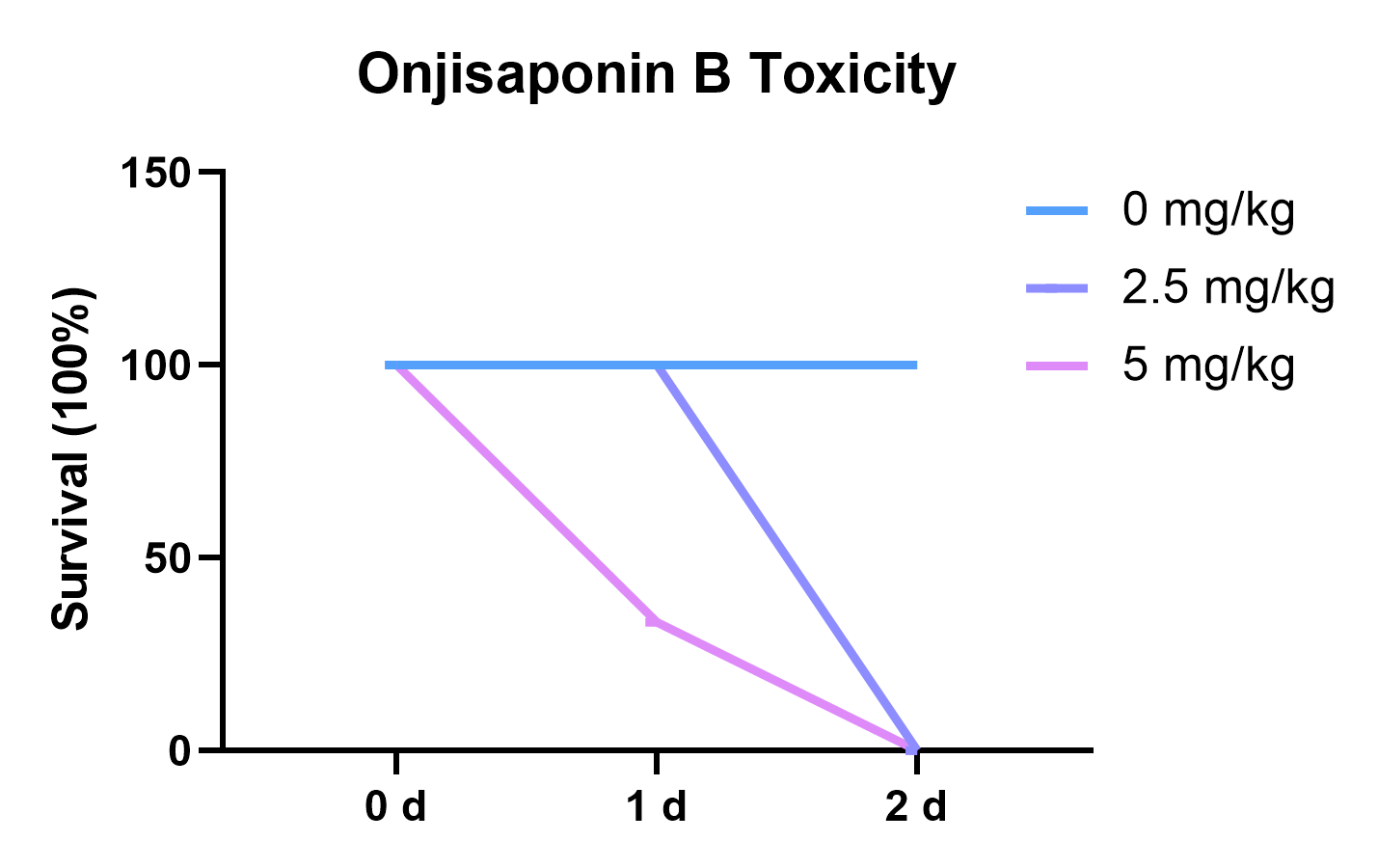

Supplement: Supplementary file 22 — Figure EV5 Source Data [file 44321_2025_260_MOESM22_ESM.zip › Figure EV5/EV5I/Onjisaponin B Toxicity.tif]
