# Supplementary material for: Reelin-LRP8 signaling mediates brain dissemination of breast cancer cells via abluminal migration
Source: EMBO Mol Med. 2025 Jun 12;17(8):1983–2010. doi: 10.1038/s44321-025-00260-0 (PMC12339728; doi:10.1038/s44321-025-00260-0)
Supplement: Supplementary file 23 — Expanded View Figures [file 44321_2025_260_MOESM23_ESM.pdf]

Expanded View Figures

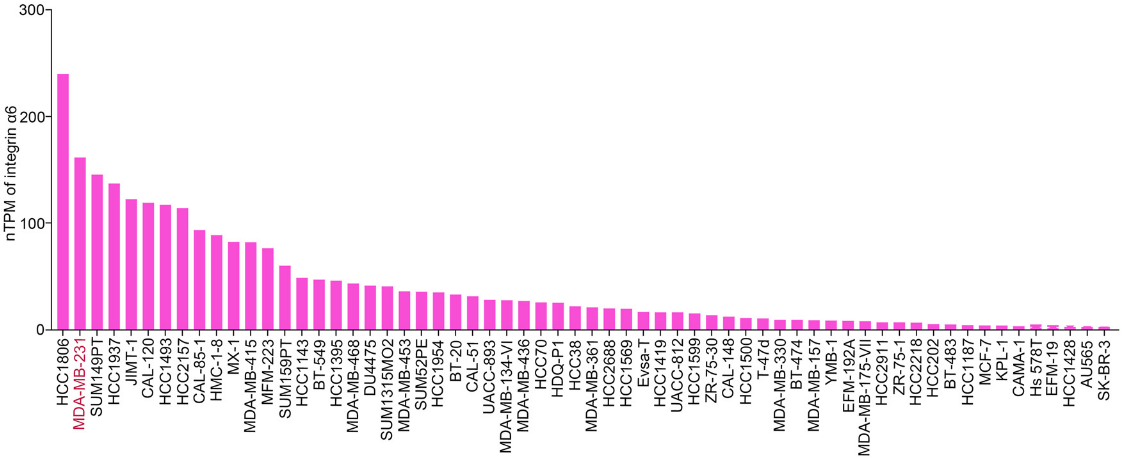

Figure EV1. Integrin α6 mRNA expression in various human breast cancer cell lines.

Integrin α6 mRNA expression in various human breast cancer cell lines. Data were obtained from Human Protein Atlas.

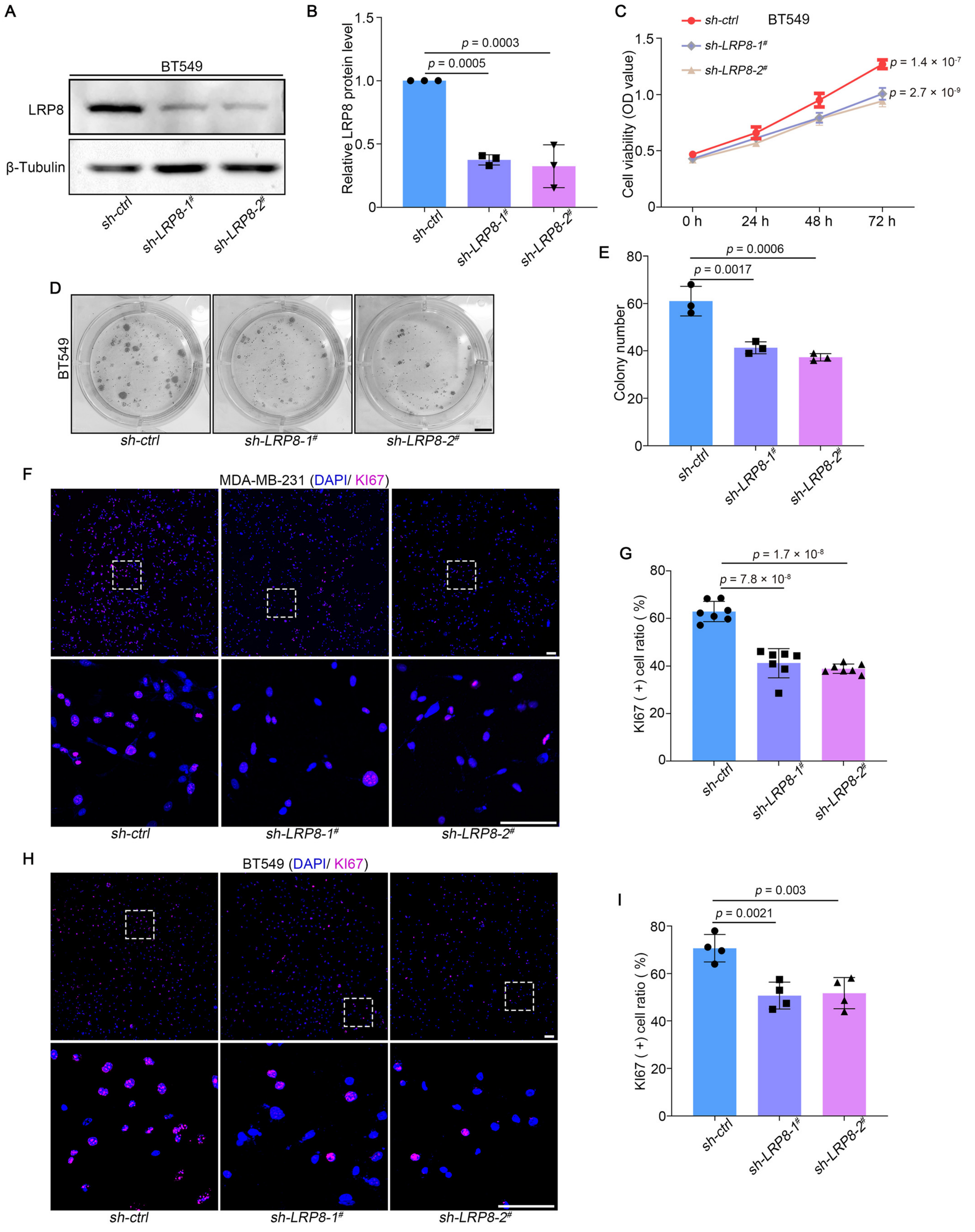
A
BT549
LRP8
β-Tubulin
sh-ctrl
sh-LRP8-1#
sh-LRP8-2#
B
Relative LRP8 protein level
p = 0.0003
p = 0.0005
C
sh-ctrl
sh-LRP8-1#
sh-LRP8-2#
BT549
Cell viability (OD value)
p = 1.4 × 10-7
p = 2.7 × 10-9
0 h
24 h
48 h
72 h
D
BT549
E
Colony number
p = 0.0006
p = 0.0017
F
MDA-MB-231 (DAPI/ KI67)
G
KI67 (+) cell ratio (%)
p = 1.7 × 10-8
p = 7.8 × 10-8
H
BT549 (DAPI/ KI67)
I
p = 0.003
p = 0.0021

 © The Author(s)

Figure EV2. ***LRP8*** **knockdown inhibits proliferation of TNBC cells.**

(**A**) BT549 cells transfected with *sh-ctrl*, *sh-LRP8-1#* and *sh-LRP8-2#* were subjected for western blot assay to analyse the protein level of LRP8. (**B**) Quantitative analysis showed the relative LRP8 protein level normalized to β-tubulin, $n = 3$. (**C**) Cell viability of BT549 cells transfected with *sh-ctrl*, *sh-LRP8-1#* and *sh-LRP8-2#* was evaluated using the CCK-8 assays, $n = 3$. (**D**, **E**) Images and quantification of the number of colonies formed from BT549 cells transfected with *sh-ctrl*, *sh-LRP8-1#* and *sh-LRP8-2#*. Scale bar: 500 μm, $n = 3$. (**F**) MDA-MB-231 cells transfected with *sh-ctrl*, *sh-LRP8-1#* and *sh-LRP8-2#* were immunostained with KI67 (pure signal) and nuclei were stained by DAPI (blue signal). Scale bar: 100 μm. (**G**) Quantitative analysis of KI67 positive cell rate in three cell lines of MDA-MB-231 cells in vitro, $n = 7$. $P = 7.8 \times 10^{-8}$ (*sh-ctrl* vs. *sh-LRP8-1#*), $P = 1.7 \times 10^{-8}$ (*sh-ctrl* vs. *sh-LRP8-2#*). (**H**) BT549 cells transfected with *sh-ctrl*, *sh-LRP8-1#* and *sh-LRP8-2#* were immunostained with KI67 (pure signal) and nuclei were stained by DAPI (blue signal). Scale bar: 100 μm. (**I**) Quantitative analysis of KI67 positive cell rate in three cell lines of BT549 cells in vitro, $n = 4$. Data information: data are shown as mean ± SD, P values were analyzed with one-way ANOVA test (**B**, **E**, **G**, **I**) and two-way ANOVA test (**C**). Source data are available online for this figure.

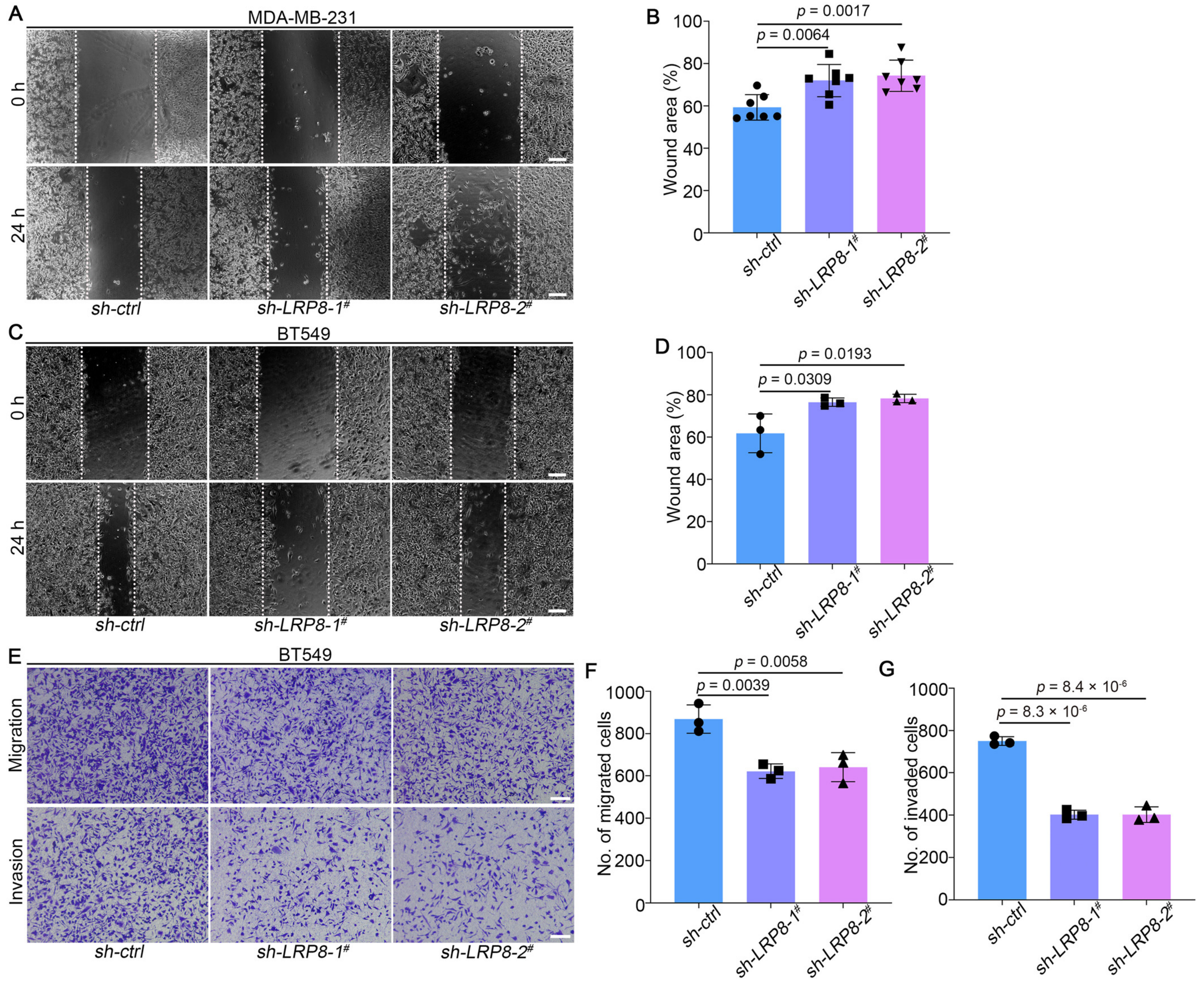

Figure EV3. ***LRP8* knockdown inhibits the migration and invasion of TNBC cells.**

(**A**, **B**) The investigation of wound healing capabilities in MDA-MB-231 cells transfected with *sh-ctrl*, *sh-LRP8-1#* and *sh-LRP8-2#* were depicted. Assessment of the wound area ratio in 24 h in comparison to baseline measurements at 0 h. Scale bar: 200 μm, $n = 7$. (**C**, **D**) Investigation of wound healing capabilities in BT549 cells transfected with *sh-ctrl*, *sh-LRP8-1#* and *sh-LRP8-2#*. Assessment of the wound area ratio in 24 h in comparison to baseline measurements at 0 h. Scale bar: 200 μm, $n = 3$. (**E**–**G**) Transwell migration assay and matrigel transwell invasion assay in BT549 cells transfected with *sh-ctrl*, *sh-LRP8-1#* and *sh-LRP8-2#*. Scale bar: 200 μm, $n = 3$. $P = 8.3 \times 10^{-6}$ (*sh-ctrl* vs. *sh-LRP8-1#*), $P = 8.4 \times 10^{-6}$ (*sh-ctrl* vs. *sh-LRP8-2#*). Data information: data are shown as mean ± SD, *P* values were analyzed with one-way ANOVA test (**B**, **D**, **F**, **G**). Source data are available online for this figure.

 © The Author(s)

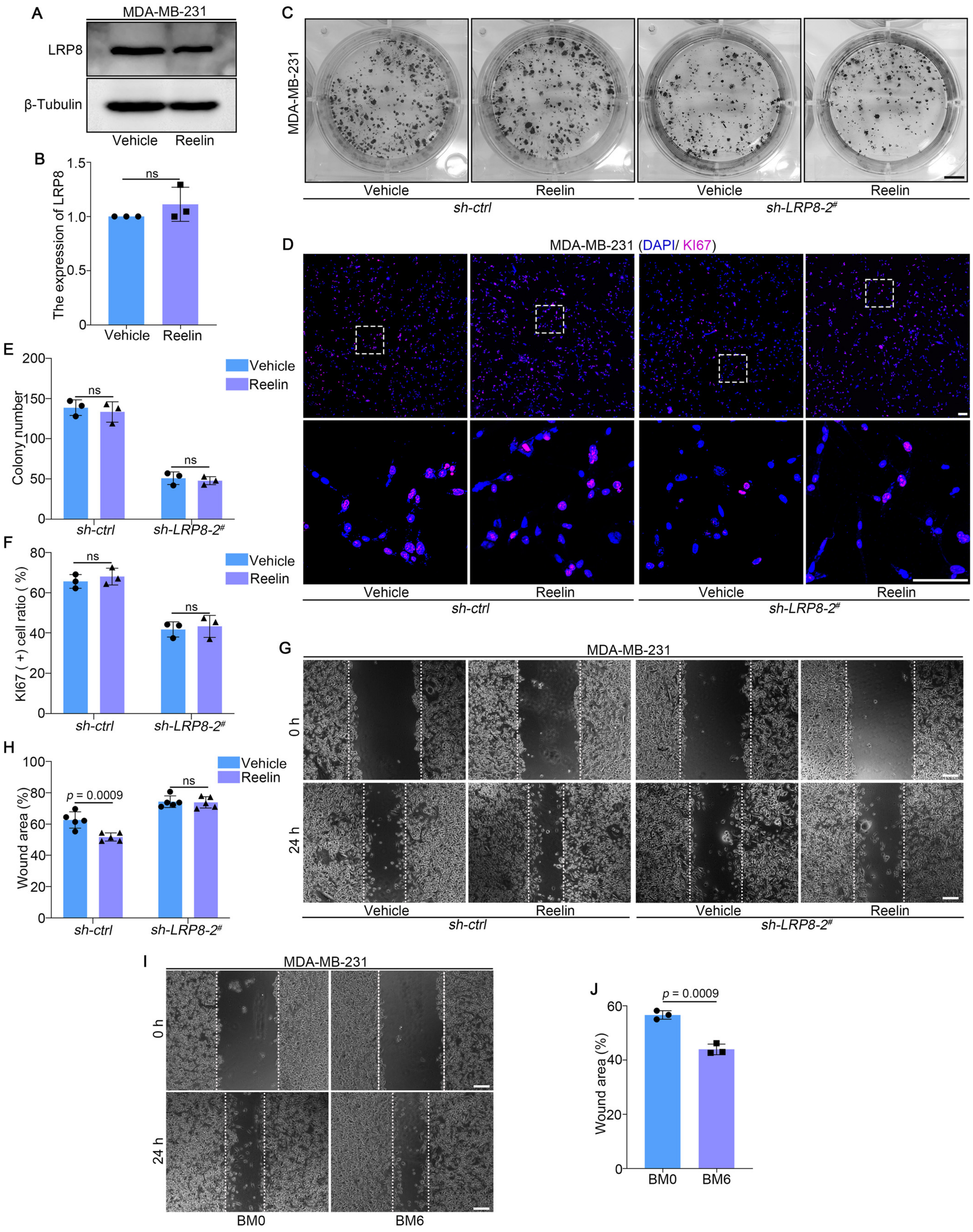
A
MDA-MB-231
LRP8
β-Tubulin
Vehicle Reelin
B
The expression of LRP8
ns
Vehicle Reelin
C
MDA-MB-231
Vehicle Reelin Vehicle Reelin
sh-ctrl sh-LRP8-2#
D
MDA-MB-231 (DAPI/ KI67)
Vehicle Reelin Vehicle Reelin
sh-ctrl sh-LRP8-2#
E
Colony number
Vehicle
Reelin
ns
sh-ctrl sh-LRP8-2#
F
KI67 (+) cell ratio (%)
Vehicle
Reelin
ns
sh-ctrl sh-LRP8-2#
G
MDA-MB-231
0 h
24 h
Vehicle Reelin Vehicle Reelin
sh-ctrl sh-LRP8-2#
H
Wound area (%)
Vehicle
Reelin
p = 0.0009
ns
sh-ctrl sh-LRP8-2#
I
MDA-MB-231
0 h
24 h
BM0 BM6
J
Wound area (%)
p = 0.0009
BM0 BM6

◀ **Figure EV4. Reelin-LRP8 pathway may not influence the proliferation of MDA-MB-231 cells, but increased the migration.**

(**A**, **B**) MDA-MB-231 cells were subjected for western blot assay to analyze the protein level of LRP8 after Reelin treatment. $n = 3$. (**C**, **E**) Images and quantification of the number of colonies formed from MDA-MB-231 cells transfected with *sh-ctrl* and *sh-LRP8-2#*, following Reelin treatment. Scale bar: 500 μm, $n = 3$. (**D**, **F**) MDA-MB-231 cells transfected with *sh-ctrl* and *sh-LRP8-2#* were immunostained with KI67 (pure signal) and nuclei were stained by DAPI (blue signal) after Reelin treatment. Scale bar: 100 μm, $n = 3$. (**G**) Wound healing capabilities were investigated in MDA-MB-231 cells transfected with *sh-ctrl* and *sh-LRP8-2#* after Reelin treatment. Scale bar: 200 μm. (**H**) Assessment of the wound area ratio in 24 h in comparison to baseline measurements at 0 h. $n = 5$. (**I**, **J**) Investigation and quantitative analysis of wound healing capabilities of BM0 cells and BM6 cells. Scale bar: 200 μm, $n = 3$. Data information: data are shown as mean ± SD, *P* values were analyzed with two-way ANOVA test (**E**, **F**, **H**) and unpaired Student's *t* test (**B**, **J**). ns non-significant. Source data are available online for this figure.

 © The Author(s)

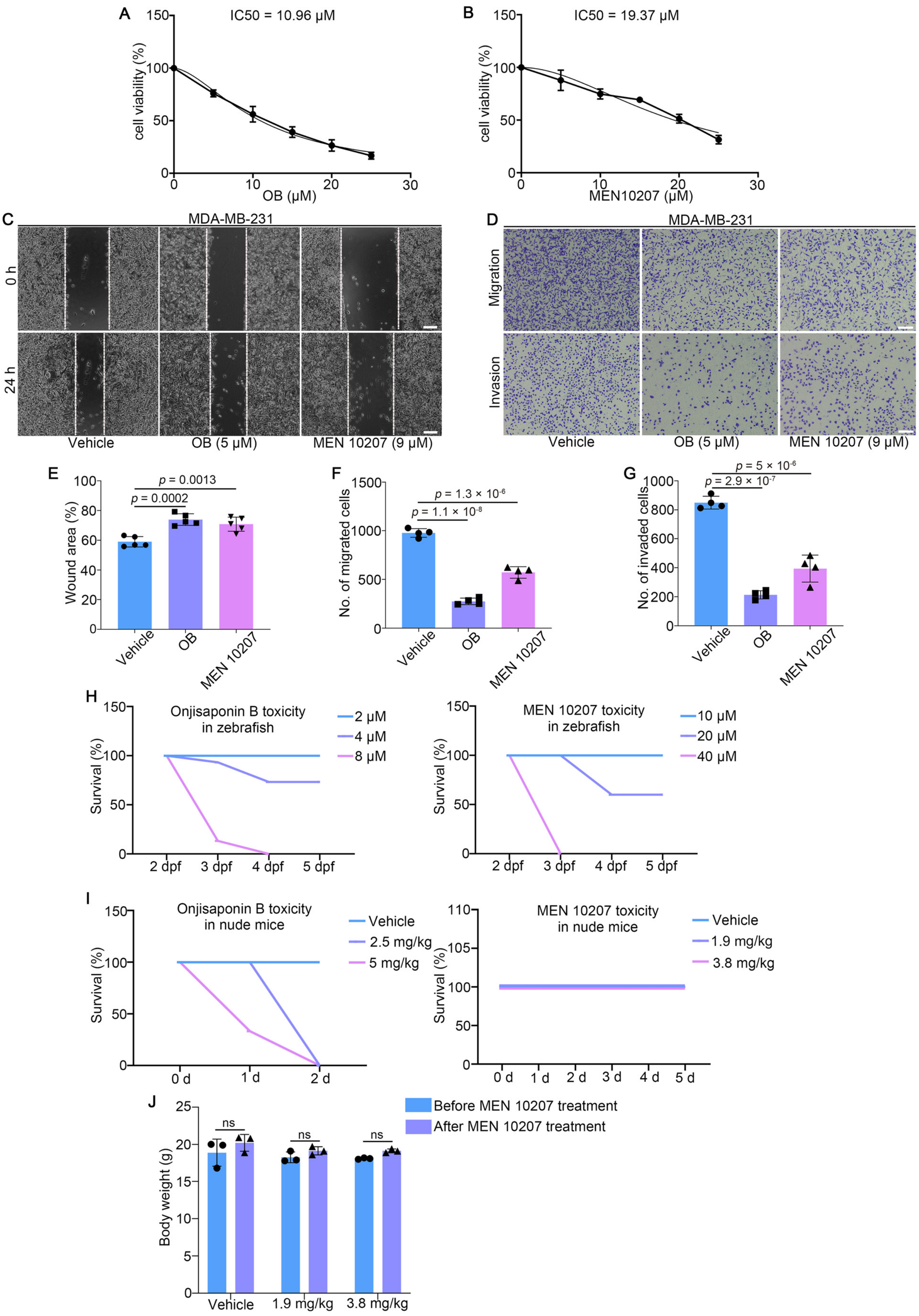
A
IC50 = 10.96 µM
cell viability (%)
OB (µM)
B
IC50 = 19.37 µM
cell viability (%)
MEN10207 (µM)
C
MDA-MB-231
0 h
24 h
Vehicle
OB (5 µM)
MEN 10207 (9 µM)
D
MDA-MB-231
Migration
Invasion
Vehicle
OB (5 µM)
MEN 10207 (9 µM)
E
p = 0.0013
p = 0.0002
Wound area (%)
Vehicle
OB
MEN 10207
F
p = 1.3 × 10-6
p = 1.1 × 10-8
No. of migrated cells
Vehicle
OB
MEN 10207
G
p = 5 × 10-6
p = 2.9 × 10-7
No. of invaded cells
Vehicle
OB
MEN 10207
H
Onjisaponin B toxicity in zebrafish
2 µM
4 µM
8 µM
Survival (%)
2 dpf
3 dpf
4 dpf
5 dpf
MEN 10207 toxicity in zebrafish
10 µM
20 µM
40 µM
Survival (%)
I
Onjisaponin B toxicity in nude mice
Vehicle
2.5 mg/kg
5 mg/kg
Survival (%)
0 d
1 d
2 d
MEN 10207 toxicity in nude mice
Vehicle
1.9 mg/kg
3.8 mg/kg
Survival (%)
0 d
1 d
2 d
3 d
4 d
5 d
J
Before MEN 10207 treatment
After MEN 10207 treatment
ns
Body weight (g)
Vehicle
1.9 mg/kg
3.8 mg/kg

Figure EV5. OB and MEN 10207 treatment inhibit the migration of MDA-MB-231 cells in vitro and toxicity of OB and MEN 10207 is detected in zebrafish embryos and nude mice.

(**A**, **B**) Effects of OB and MEN 10207 on the cell viability of MDA-MB-231 cells at 24 h, $n = 3$. (**C**) Investigation of wound healing capabilities in MDA-MB-231 cells treated with vehicle, OB and MEN 10207. Scale bar: 200 μm. (**D**) Transwell migration assay and matrigel transwell invasion assay in MDA-MB-231 cells treated with vehicle, OB and MEN 10207. Scale bar: 200 μm. (**E**) Assessment of the wound area ratio in 24 h in comparison to baseline measurements at 0 h. Scale bar: 200 μm, $n = 5$. (**F**) Quantitative analysis of **D** showed the migratory abilities. $n = 4$. $P = 1.1 \times 10^{-8}$ (Vehicle vs. OB), $P = 1.3 \times 10^{-6}$ (Vehicle vs. MEN 10207). (**G**) Quantitative analysis of **D** showed the invasive abilities. $n = 4$. $P = 2.9 \times 10^{-7}$ (Vehicle vs. OB), $P = 5 \times 10^{-6}$ (Vehicle vs. MEN 10207). (**H**) Quantification of the proportion of surviving embryos following treatment with OB and MEN 10207 starting at 2 dpi. $n > 10$. (**I**) Quantification of the proportion of surviving nude mice (approximately 8 weeks old) following treatment with OB and MEN 10207. $n = 3$. (**J**) Body weight changed in nude mice before and after MEN 10207 treatment. $n = 3$. Data information: data are shown as mean ± SD, P values were analyzed with one-way ANOVA test (**E**, **F**, **G**) and two-way ANOVA test (**J**). ns non-significant. Source data are available online for this figure.

© The Author(s)
